# ﻿Integrating morphology, phylogeny, substrate, and distribution: clarifying the major phylogenetic framework of Pluteussect.Hispidoderma (*Agaricales*, *Pluteaceae*) and describing 18 species

**DOI:** 10.3897/imafungus.16.154329

**Published:** 2025-07-29

**Authors:** Zheng-Xiang Qi, Li-Bo Wang, Ke-Qing Qian, Li-Li Shi, Jia-Jun Hu, Yong-Lan Tuo, Gu Rao, Muharagi Samwel Jacob, Rui-Peng Liu, Ming-Hao Liu, Di-Zhe Guo, Ya-Jie Liu, Bo Zhang, Xiao Li, Yu Li

**Affiliations:** 1 Engineering Research Center of Edible and Medicinal Fungi, Ministry of Education, Jilin Agricultural University, Changchun, China; 2 College of Mycology, Jilin Agricultural University, Changchun, China; 3 Joint Laboratory of International Cooperation in Modern Agricultural Technology, Ministry of Education, Jilin Agricultural University, Changchun, China; 4 Northeast Asian Specialty Germplasm Resources Innovation Centre, Jilin Agricultural University, Changchun, China; 5 Industrial Development Institute for Plants, Animals and Fungi Integration of Biyang County, Biyang, China; 6 School of Life Science, Zhejiang Normal University, Jinhua, China; 7 College of Life Sciences, Nanjing Normal University, Nanjing, China; 8 Hebei Normal University of Science and Technology, Qinghuangdao, China

**Keywords:** *Hispidoderma*, phylogenetic, substrate, taxonomy, wood-rot fungi

## Abstract

*Pluteus* is a genus of wood-rot fungi with considerable ecological importance in forest ecosystems. Within this genus, sectionHispidoderma is distinctively characterized by a specific combination of pileipellis structures (trichoderm, hymeniderm, or trichohymeniderm) and non-metuloid hymenial cystidia, which together provide key morphological basis for section delimitation. In this study, we combined genetic data from three gene regions (large subunit ribosomal RNA [LSU], internal transcribed spacer [ITS], and translation elongation factor 1-alpha [*tef1*]) to construct the major phylogenetic framework of sectionHispidoderma. Our analysis revealed three primary clades (/*plautus* clade, /*leoninus* clade, /*umbrosus* clade) and one lineage (/*pantherinus* lineage). We subsequently identified several important morphological features and correlated them with phylogenetic relationships to reveal shared characteristics among species within each evolutionary clade. Building on this framework, we constructed phylogenetic trees using three datasets (ITS, *tef1* and a combined ITS+*tef1*) to analyze the phylogenetic structure and species relationships within each clade and lineage. By comprehensively integrating morphology, phylogenetic data, substrate preferences, and species distribution, we identified 18 species: nine new species (*P.albivillus*, *P.baishanzuensis*, *P.costatus*, *P.hinnuleus*, *P.jilinensis*, *P.piceicola*, *P.spaniophyllus*, *P.tenuipileus*, and *P.ultraputripiceae*), one new record for China (*P.ussuriensis*), seven previously known species (*P.granularis*, *P.leoninus*, *P.longistriatus*, *P.umbrosus*, *P.umbrosoides*, *P.variabilicolor*, and *P.velutinus*), and one species of uncertain taxonomic affinity (P.aff.semibulbosus). This study provides detailed documentation, including line drawings and color photographs of the 18 identified species, along with phylogenetic analyses of their evolutionary relationships. Additionally, we present a thorough identification key for the 25 species of sect.Hispidoderma found in China. By clarifying the delineation of clades and species boundaries within sect.Hispidoderma, this work significantly advances our understanding of the taxonomy of this ecologically important fungal group.

## ﻿Introduction

The genus *Pluteus*, classified under the *Basidiomycota*, *Agaricomycetes*, *Agaricales*, *Pluteaceae*, was formally defined by Fries in 1863. The genus *Pluteus* is recognized by its free lamellae, pinkish spore print, inverse hymenophoral trama, smooth globose to ellipsoidal basidiospores, few oblong and various forms of pleurocystidia, often accompanied by cheilocystidia. It primarily grows in decaying wood and is distributed worldwide (Vellinga and Schreurs 1985; Singer 1986; Justo et al. 2011a, b).

*Pluteus* has been subdivided into three sections based on the characteristics of the cystidia and pileipellis viz. sect.Pluteus, sect.Hispidoderma, and sect.Celluloderma (Lange 1917; Imai 1938; Singer 1956). Furthermore, sectionHispidoderma is characterized by a pileipellis, which is a trichoderm composed of elongated cells and thin-walled pleurocystidia. Singer (1958, 1986) traditionally identified Pluteussect.Hispidoderma as encompassing species characterized by non-metuloid pleurocystidia and a pileipellis consisting of elongated elements that form a cutis, hymeniderm, or trichoderm. However, Vellinga and Schreurs (1985) proposed a modification to this classification by dividing sect.Hispidoderma was divided into two taxonomic units based on the structure of the pileipellis. A new section, viz. Pluteussect.Villosi, was established to encompass species characterized by a cutis-like pileipellis, while the other species, which include those characterized by a trichoderm pileipellis or a hymeniderm with cylindrical to fusiform elements, were reclassified under Pluteussect.Celluloderma subsect. *Hispidodermini*.

Singer’s classification is widely recognized as the most accepted system for the infrageneric organization of *Pluteus*. Molecular data from Justo et al. (2011a, b) have supported this classification, albeit with some rearrangements. The molecular analyses conducted by Justo et al. (2011a, b) demonstrated that species exhibiting non-metuloid cystidia and a cutis-like pileipellis should be classified into sect.Celluloderma, rather than Pluteussect.Hispidoderma (Singer 1958, 1986) or sect.Villosi (Vellinga and Schreurs 1985). Vellinga (1990) and Justo et al. (2011a, b) characterized sect.Hispidoderma as having a hymenidermal or trichodermal pileipellis composed of long, elongated elements (Q > 3) that vary in shape and size. Subsequently, in their classification of sect.Hispidoderma, Kaygusuz et al. (2019, 2021) expressed a viewpoint that aligns closely with that of Justo et al. (2011a, b).

Building upon these valuable advances, some aspects of Pluteussect.Hispidoderma taxonomy still present opportunities for further refinement. Particularly, integrating morphological observations, molecular data, ecological substrate preferences, and geographic distribution patterns offers promising avenues to enhance our understanding of species boundaries within this section. To address these integration opportunities, our current study builds upon and expands the framework established by Justo et al. (2011) through four complementary objectives: (i) Refining the phylogenetic framework for sect.Hispidoderma with additional LSU+ITS+*tef1* data from previously unsampled taxa; (ii) Integration of stable morphological characteristics to analyze the relationship between morphology and phylogeny among sect.Hispidoderma species; (iii) Analysis of phylogenetic relationships within the three clades and one dataset using combined ITS+*tef1* data matrices as well as separate ITS and *tef1* data matrices; (iv) Identification of 18 species through comprehensive integration of morphological features, phylogenetic data, substrate preferences, and geographic distribution. By achieving these objectives, this study seeks to advance our understanding of these ecologically significant fungi and establish a foundation for future taxonomic and ecological studies of *Pluteus*.

## ﻿Materials and methods

### ﻿Specimen collection and morphological characterization

We went out in the field in forested areas and collected *Pluteus* specimens, with a primary focus on decaying wood substrates. Upon discovering specimens, we photographed each basidiomata in situ and recorded detailed morphological characteristics (shape, size, color, odor, and other macroscopic characteristics of the basidiomata pileus, lamellae, and stipes). Color codes are from the Munsell Soil Color Chart (Munsell 2009). Approximately 15 g of fresh context and lamellae were desiccated in a Ziplock bag containing silica gel and subsequently brought back to the laboratory for DNA extraction. Fresh basidiomata were dried at 40 °C–45 °C using a plant drying oven and were preserved in the Fungarium of Jilin Agricultural University (**FJAU**).

The examination of microstructural characteristics was based on desiccated specimens. The desiccated specimens underwent rehydration in 94% ethanol for microscopic analysis and were subsequently mounted in 3% potassium hydroxide (KOH), 1% Congo Red, and Melzer’s Reagent. This process was conducted utilizing a light microscope (ZEISS, DM1000, Oberkochen, Germany). Specifically, the following symbols were used in the description: [n/m/p] indicates that ‘n’ randomly selected basidiospores from ‘m’ basidiomata of ‘p’ collections were measured, ‘avL’ means the average length of basidiospores, except for the extreme values, ‘avW’ refers to the average width of the basidiospores, except the extreme values, ‘Q’ represents the quotient of the length and width of a single basidiospore in side view, while ‘avQ’ refers to the average Q value of all basidiospores ± standard deviation. The dimensions of the basidiospores are given as (a) b–c (d). The range of b–c contains a minimum of 90% of the measured values. Extreme values (i.e., a and b) are given in parentheses (Vellinga 1988; Qi et al. 2022; Qi et al. 2024; Zhu and Bau 2024).

### ﻿DNA extraction and PCR amplification

The total DNA from the specimens was extracted using the new plant genomic DNA extraction kit from Jiangsu Kangwei Century Biotechnology Limited Company, P.R. China. Subsequently, sequences of the internal transcribed spacer (ITS) region, the large subunit of the nuclear ribosomal RNA gene (LSU), and translation elongation factor 1-α (*tef1*) were used for phylogenetic analyses. The amplification primers used for the nr ITS: ITS1–5.8S–ITS2 regions were ITS1F and ITS4/ITS4B (White et al. 1990), for the nLSU regions were LROR and LR5 (Hopple and Vilgalys 1994) and for the *tef1* regions were EF1–983F and EF1–1567R (Rehner and Buckley 2005). The amplification reactions were carried out in a 25 µL system. The PCR mixture was composed of the following components: 13.5 µL of dd H_2_O, 5 µL of 10 × Taq Buffer, 1 µL of 10 mM dNTPs, 1 µL of 10 mM upstream primer, 1 µL of 10 mM downstream primer, 2 µL of DNA sample, 1.5 µL of 2 U/mm Taq Polymerase. The cycle parameters were as follows: 5 min at 98 °C, 30 s at 98 °C, 30 s at 55 °C, 1 min at 72 °C for 40 cycles, 7 min at 72 °C, and storage at 4 °C (Ševcíková et al. 2022). The PCR product was subjected to 1% agarose gel electrophoresis. The purified PCR products were sent to Sangon Biotech Limited Company, P.R. China, for sequencing using the Sanger method. The sequencing results were clipped with Seqman 7.1.0 (Swindell and Plasterer 1997) and submitted to GenBank (https://www.ncbi.nlm.nih.gov/genbank/).

### ﻿Sequence analysis and phylogenetic reconstruction

The species that morphologically resemble the taxa studied here, and sequences exhibiting high sequence similarity after the BLAST analysis were selected for the phylogenetic analysis (Justo et al. 2011b, 2012; Menolli et al. 2015a; Kaygusuz et al. 2019; Malysheva et al. 2020; Kaygusuz et al. 2021; Qi et al. 2022; Ševcíková et al. 2022; Malysheva et al. 2023; Justo et al. 2025) and the ITS, LSU, and *tef1* sequences of these species are shown in Table 2. The dataset included 319 representative sequences from the ITS, LSU, and *tef1* genes, exhibiting the highest similarity to *Pluteus* spp., along with two sequences of *Pluteusromellii* (Britzelm.) Lapl. as an outgroup.

Sequence alignment and phylogenetic analyses: First, for related species consisting of ITS, LSU, and *tef1* sequences, sequence alignment was initially performed for ITS, LSU, and *tef1* using the “automatic” strategy and normal alignment mode of MACSE V2.03 (Ranwez et al. 2018) and MAFFT (Katoh and Standley 2013), respectively. Subsequently, the alignments were manually adjusted in BIOEDIT v7.1.3 (Hall 1999). Subsequently, the ITS, LSU, and *tef1* sequences were aligned and combined using PHYLOSUIT V1.2.2 (Zhang et al. 2020). ModelFinder (Kalyaanamoorthy et al. 2017) was used to select the best–fit models using the Bayesian Information Criterion (BIC). In this case, the Maximum Likelihood (ML) analyses were performed in IQTREE 1.6.8 (Nguyen et al. 2015), and the Bayesian inference phylogenies were performed in MRBAYES 3.2.6 (Ronquist et al. 2012) (two parallel runs, 2,000,000 generations), in which the initial 25% of sampled data were discarded as burn-in. The above software was integrated within PHYLOSUITE 1.2.2 (Zhang et al. 2020). The ML phylogenetic tree was evaluated using the bootstrap method with a bootstrap value of 1,000 replicates. BI determined that the analysis reached smoothness with a variance of less than 0.01 and terminated the calculation. Finally, the evolutionary tree was visualized using FIGTREE v1.4.

### ﻿Genetic distance analysis

Evolutionary differentiation studies on the selected ITS and *tef1* sequence datasets were performed using MEGA X software. Phylogenetic distances were calculated based on the Maximum Composite Likelihood method (Tamura et al. 2004), which represents sequence differences averaged across all inferred species, expressed as the number of base substitutions per site. Site variation rates were modeled using a gamma distribution (shape parameter set to 1). During analysis, ambiguous positions for each sequence pair were eliminated (using the pairwise deletion option). The final ITS dataset contained 754 positions, while the tef1 dataset contained 591 positions. To obtain reliable statistical support, the bootstrap method with 1000 replicates was employed to estimate standard errors.

To facilitate comparison with studies reporting simple percentage differences, we provide a conversion between uncorrected sequence differences and our model-corrected genetic distances: 2% uncorrected difference ≈ 0.020–0.022 genetic distance; 3% ≈ 0.031–0.034; 4% ≈ 0.042–0.046; and 5% ≈ 0.053–0.058. This conversion enables meaningful comparison with published studies employing different distance calculation methods, particularly when evaluating species delimitation thresholds in fungal taxonomy.

### ﻿Abbreviations

The basidiospore measurements were recorded as length × width (L × W). The variation in the ratio of L to W among the studied specimens was denoted as Q. avQ represented the average Q value of all the basidiospores ± the standard deviation.

## ﻿Results

### ﻿Molecular phylogeny

**Figure 1.**
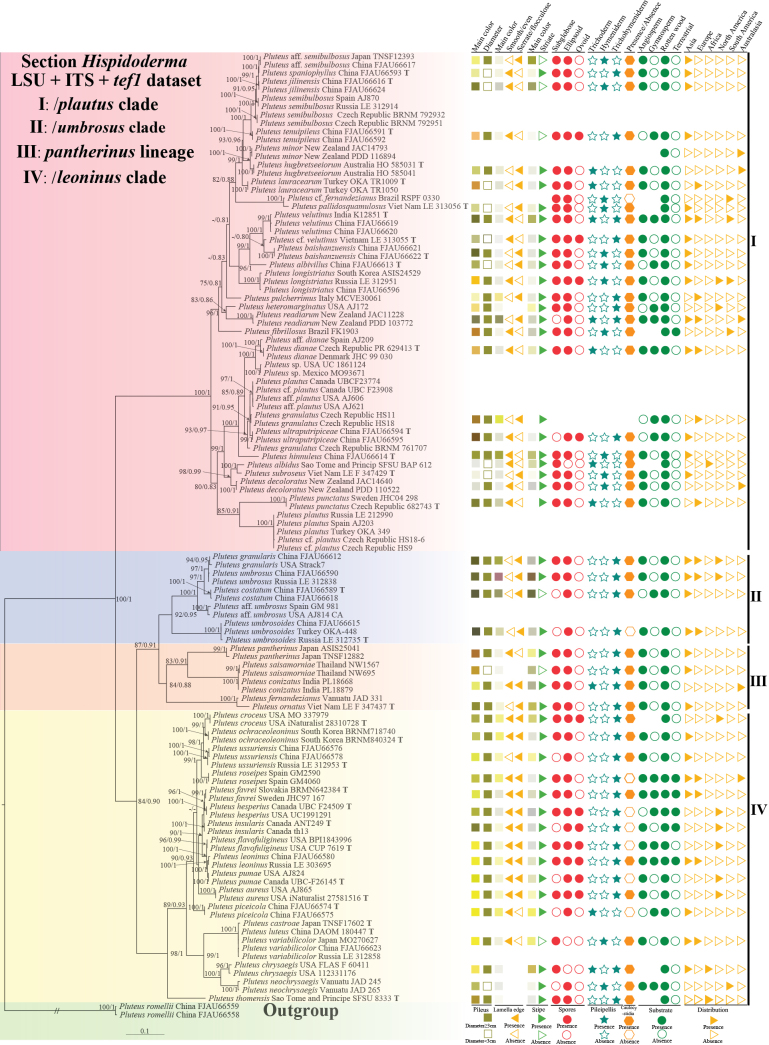
Phylogenetic tree of the sect.Hispidoderma of the genus *Pluteus*. The best tree from the ML and BI analysis of the LSU+ITS+*tef1* dataset. The two values of internal nodes respectively represent the Maximum Likelihood bootstrap (MLBP)/Bayesian posterior probability (BIPP). Nodes with a bootstrap probability (BP) ≥ 80 and a Bayesian posterior probability (PP) ≥ 0.80 are indicated in the phylogram.

The ITS dataset of this study consists of 193 sequences and 691–735 characters (gaps included). The LSU dataset comprises 57 sequences and 893 characters (gaps included). The *tef1* dataset comprises 69 sequences and 535–588 characters (gaps included). The combined dataset of LSU+ITS+*tef1* consists of 319 sequences, representing 63 species, which includes 32 type specimens and 1 outgroup. A total of 147 sequences, comprising 51 ITS, 47 LSU and 46 *tef1* sequences, were newly produced as part of this study (Table 2). The phylogram resulting from the ML analyses exhibited a topology that was largely consistent with the one obtained from BI analyses. Consequently, only the Bayesian phylogenetic tree incorporating Maximum Likelihood Bootstrap (MLB) and Bayesian Posterior Probability (BPP) values is indicated in Fig. 1. The components and best models for all the dataset matrices are listed in Table 1. The accession numbers for the newly generated ITS, LSU, and *tef1* sequences, along with those of other sequences sourced from GenBank and UNITE that were employed in the phylogenetic analysis, are detailed in Table 2.

**Table 1.** Information of matrixes used in phylogenetic analyses from all the Dataset.

Datasets	Matrixes component	ITS		*tef1*		ITS+ *tef1*	
		ML	Mrbayes	ML	Mrbayes	ML	Mrbayes
/*platus* clade	ITS: 711 bp; *tef1*: 535 bp;	TIM2+I+G4	HYK	TIM2+I+G4	HYK	TIM2+I+G	JC
/*leoninus* clade	ITS: 735 bp; *tef1*: 588 bp;	TIM2e+G4	SYM	TIM2e+G	SYM	TIM2+I+G	JC
/*umbrosus* clade	ITS: 691 bp; *tef1*:555 bp;	HKY85+I	K80 (K2P)	TN (TN93)	K80 (K2P)	HKY+I/GTR+G	JC
SectionHispidoderma	LSU:893 bp; ITS: 691 bp; *tef1*: 535 bp;	TIM2e+I+G4	HYK	/	/	/	/

**Table 2.** Names, collection numbers, reported countries, and corresponding GenBank accession numbers for the taxa used in this study. Bold fonts are the sequences to be determined in this study.

Species	Collection	Country	GenBank No.			Reference
			ITS	LSU	*tef1*	
P.aff.dianae	AJ209	Spain	HM562055	-	-	Justo et al. 2011b
P.aff.leoninus	SF21	USA	HM562190	-	-	Justo et al. 2011b
P.aff.leoninus	SF19	USA	HM562188	-	-	Kaygusuz et al. 2019
P.aff.plautus	AJ621	USA	KR022010	-	-	Malysheva et al. 2016
P.aff.plautus	AJ606	USA	KR022011	-	-	Malysheva et al. 2016
P.aff.plautus	AJ226	Spain	KR022025	-	-	Menolli et al. 2015a
P.aff.plautus	ACaballero 783	Spain	KR022026	-	-	Menolli et al. 2015a
P.aff.semibulbosus	TNSF12393	Japan	HM562090	-	-	Justo et al. 2011b
**P.aff.semibulbosus**	**FJAU66617**	**China**	**PQ810766**	**PQ810744**	**PQ811050**	**This study**
P.aff.umbrosus	AJ814	USA	PP950752	-	-	Justo et al. 2025
P.aff.umbrosus	GM 981	Spain	PP950751	-	-	Justo et al. 2025
*P.albidus*	SFSU:BAP 612	Sao Tome and Principe	MG968798	-	-	Desjardin and Perry 2018
***P.albivillus***	**FJAU66613 T**	**China**	**PQ810759**	**PQ810736**	**PQ811046**	**This study**
*P.aureus*	AJ865	USA	PP950743	-	PP950354	Justo et al. 2025
*P.aureus*	iNaturalist 27581516 T	USA	ON006979	-	-	Justo et al. 2025
***P.baishanzuensis***	**FJAU66621**	**China**	**PQ810762**	**PQ810739**	**PQ811049**	**This study**
***P.baishanzuensis***	**FJAU66622 T**	**China**	**PQ810761**	**PQ810738**	**PQ811048**	**This study**
P.cf.fernandezianus	RSPF 0330	Brazil	JQ065028	-	-	Menolli et al. 2015a
P.cf.plautus	HS9	Czech Republic	MH656436	-		Ševcíková et al. 2020
P.cf.plautus	HS18	Czech Republic	MH656437	-		Ševcíková et al. 2020
P.cf.plautus	UBC F23908	Canada	KJ146724	-	-	Kaygusuz et al. 2019
P.cf.velutinus	LE 313055 T	Vietnam	MT611241	-		Kaygusuz et al. 2021
*P.chrysaegis*	LE F-347436	Viet Nam	OQ732741	-	-	Malysheva et al. 2023
*P.chrysaegis*	GDGM:42376	China	MH059514	-	-	Hosen et al. 2018
*P.chrysaegis*	FLAS-F-60411	USA	MF153092	-	-	Hosen et al. 2018
*P.chrysaegis*	iNaturalist 112331176	USA	PP950745	-	PP950356	Justo et al. 2025
*P.chrysaegis*	GDGM 42376	China	MH059514	-	-	Justo et al. 2025
*P.conizatus*	JAD 245	Vanuatu	OM060369	-	-	Kaygusuz et al. 2019
*P.conizatus*	JAD 244	Vanuatu	OM060368	-	-	Kaygusuz et al. 2019
*P.conizatus*	PL18879	India	PP950753	-	-	Justo et al. 2025
*P.conizatus*	PL18668	India	PP950754	-	-	Justo et al. 2025
***P.costatus***	**FJAU66589 T**	**China**	**PP516603**	**PP919365**	**PP551591**	**This study**
***P.costatus***	**FJAU66618**	**China**	**PQ810769**	**PQ810757**	**PQ811052**	**This study**
*P.croceus*	iNaturalist 28310728 T	USA	ON006974	-	PP950345	Justo et al. 2025
*P.decoloratus*	JAC14640	New Zealand	MN738654	-	-	Horak 2008
*P.decoloratus*	PDD:110522	New Zealand	MN738677	MN738609	-	Horak 2008
*P.dianae*	LE 312950	Russia	MH656430	-	-	Ševcíková et al. 2020
*P.dianae*	PR 629413 T	Czech Republic	MH656433	-	-	Ševcíková et al. 2020
*P.dianae*	LE 303485	Russia	MH656431	-	-	Ševcíková et al. 2020
*P.dianae*	312978 (LE)	Russia	MH656429	-	-	Ševcíková et al. 2020
*P.dianae*	213024 (LE)	Russia	FJ774076	-	-	Ševcíková et al. 2020
*P.dianae*	296318 (LE)	Russia	MH656432	-	-	Ševcíková et al. 2020
*P.dianae*	JHC99-030	Denmark	MH656481	-	-	Ševcíková et al. 2020
*P.favrei*	BRNM840323	Italy	PP950723	-	PP950335	Justo et al. 2025
*P.favrei*	JHC97 167	Sweden	PP950720	-	PP950334	Justo et al. 2025
*P.favrei*	BRMN642384 T	Slovakia	PP950718	-	-	Justo et al. 2025
*P.femandezianus*	JAD 331	Vanuatu	OM060370	-	-	Kaygusuz et al. 2019
*P.fibrillosus*	FK1903	Brazil	KR022018	-	-	Menolli et al. 2015a
*P.flavofuligineus*	CUP 7619 T	USA	PP950699	-	-	Justo et al. 2025
*P.flavofuligineus*	DLF95 24	USA	PP950704	-	PP950321	Justo et al. 2025
*P.granularis*	SF20	USA	HM562189	-	-	Justo et al. 2011b
*P.granularis*	strack7	USA	HM562069	-	-	Justo et al. 2011b
***P.granularis***	**FJAU66612**	**China**	**PQ810758**	**PQ810735**	**PQ811045**	**This study**
*P.granulatus*	BRNM 761707	Czech Republic	-	-	LR745705	Justo et al. 2025
*P.heperius*	iNat 43028505	USA	PP950729	-	PP950340	Justo et al. 2025
*P.heperius*	UBC F24509 T	Canada	PP836189	-	PP950341	Justo et al. 2025
*P.heperius*	UBC F28945	Canada	PP836190	-	PP950342	Justo et al. 2025
*P.heperius*	UC1991291	USA	PP950728	-	PP950339	Justo et al. 2025
*P.heteromarginatus*	AJ172	USA	HM562058	HM562249	-	Justo et al. 2011b
***P.hinnuleus***	**FJAU66614 T**	**China**	**PQ810768**	**PQ810746**	**PQ811064**	**This study**
*P.hubregtseorum*	X311 T	Australia	MN918531	-	LR757898	Ševcíková et al. 2021
*P.hubregtseorum*	X313	Australia	MN918532	-	-	Ševcíková et al. 2021
*P.insularis*	ANT249 T	Canada	MN992489	-	PP950330	Justo et al. 2025
*P.insularis*	th13	Canada	PP950717	-	PP950332	Justo et al. 2025
*P.insularis*	ANT264	Canada	MN992490	-	PP950331	Justo et al. 2025
***P.jilinensis***	**FJAU66616 T**	**China**	**PQ810767**	**PQ810745**	**PQ811051**	**This study**
***P.jilinensis***	**FJAU66624**	**China**	**PQ814290**	**PQ814291**	-	**This study**
*P.lauracearum*	OKA-146	Turkey	MG544922	-	-	Kaygusuz et al. 2021
*P.lauracearum*	OKA-TR1009 T	Turkey	MW600395	-	-	Kaygusuz et al. 2021
*P.lauracearum*	oKA-TR1050	Turkey	MW600396	-	-	Kaygusuz et al. 2021
*P.leoninus*	Josserand s.n.	France	HM562077	-	-	Wannathes et al. 2022
*P.leoninus*	LE 303695	Russia	KX216311	-	-	Malysheva et al. 2016
*P.leoninus*	Champ-40	France	KX449446	-	-	Malysheva et al. 2016
*P.leoninus*	AJ352	USA	JQ065027	-	-	Malysheva et al. 2016
*P.leoninus*	SF17	USA	HM562187	-	-	Justo et al. 2011b
*P.leoninus*	Halling6546	USA	HM562071	-	-	Justo et al. 2011b
*P.leoninus*	OKA-TR10	Turkey	MK123348	-	-	Justo et al. 2011b
***P.leoninus***	**FJAU66582**	**China**	**PP516610**	**PP516660**	**PP551598**	**This study**
***P.leoninus***	**FJAU66581**	**China**	**PP516611**	**PP516661**	**PP551597**	**This study**
***P.leoninus***	**FJAU66580**	**China**	**PP516612**	**PP516662**	**PP551596**	**This study**
*P.longistriatus*	LE 312951	Russia	KX216355	-	-	Malysheva et al. 2016
*P.longistriatus*	KA17-0331	South Korea	MN294886	-	-	Malysheva et al. 2016
*P.longistriatus*	ASIS24529	South Korea	KM052568	-	-	Malysheva et al. 2016
***P.longistriatus***	**FJAU66596**	**China**	**PP516605**	**PP516655**	-	**This study**
*P.luteus*	DAOM 180447 T	China	MT080030	-	-	Justo et al. 2025
*P.minor*	PDD 116894	New Zealand	MN738682	-	-	Malysheva et al. 2023
*P.minor*	JAC14793	New Zealand	MN738660	-	-	Malysheva et al. 2023
*P.minor*	JAC14348	New Zealand	MN738650	-	-	Malysheva et al. 2023
*P.neochrysaegis*	JAD 265	Vanuatu	OM060372	-	-	Kaygusuz et al. 2019
*P.ochraceoleoninus*	BRNM840324 T	South Korea	PP950741	-	PP950350	Justo et al. 2025
*P.ochraceoleoninus*	TNSF11908	Japan	HM562139	-	-	Justo et al. 2025
*P.ochraceoleoninus*	BRNM718740	South Korea	PP950740	-	-	Justo et al. 2025
*P.ornatus*	LE F-347437 T	Vietnam	OQ732738	-	-	Malysheva et al. 2023
*P.pallidosquamulosus*	LE 313056 T	Vietnam	MT611240	-	-	Malysheva et al. 2020
*P.pantherinus*	TNSF12882	Japan	HM562089	-	-	Wannathes et al. 2022
*P.pantherinus*	ASIS25041	Japan	KF692077	-	-	Wannathes et al. 2022
***P.piceicola***	**FJAU66572**	**China**	**PP516597**	**PP516649**	**PP551587**	**This study**
***P.piceicola***	**FJAU66573**	**China**	**PP516598**	**PP516650**	**PP551588**	**This study**
***P.piceicola***	**FJAU66574 T**	**China**	**PP516599**	**PP516651**	**PP551589**	**This study**
***P.piceicola***	**FJAU66575**	**China**	**PP516600**	**PP516652**	**PP551590**	**This study**
***P.piceicola***	**FJAU66570**	**China**	**PP516595**	**PP516647**	**PP551585**	**This study**
***P.piceicola***	**FJAU66571**	**China**	**PP516596**	**PP516648**	**PP551586**	**This study**
***P.piceicola***	**FJAU66569**	**China**	**PP516594**	**PP516646**	**PP551584**	**This study**
***P.piceicola***	**FJAU66601**	**China**	**PQ810770**	**PQ810747**	**PQ811053**	**This study**
***P.piceicola***	**FJAU66602**	**China**	**PQ810771**	**PQ810748**	**PQ811054**	**This study**
***P.piceicola***	**FJAU66603**	**China**	**PQ810772**	**PQ810749**	**PQ811055**	**This study**
***P.piceicola***	**FJAU66604**	**China**	**PQ810773**	**PQ810750**	**PQ811056**	**This study**
***P.piceicola***	**FJAU66605**	**China**	**PQ810774**	**PQ810751**	**PQ811057**	**This study**
***P.piceicola***	**FJAU66606**	**China**	**PQ810775**	**PQ810752**	**PQ811058**	**This study**
***P.piceicola***	**FJAU66607**	**China**	**PQ810776**	**PQ810753**	**PQ811059**	**This study**
***P.piceicola***	**FJAU66608**	**China**	**PQ810777**	**PQ810754**	**PQ811060**	**This study**
***P.piceicola***	**FJAU66609**	**China**	**PQ810778**	**PQ810755**	**PQ811061**	**This study**
***P.piceicola***	**FJAU66610**	**China**	**PQ810779**	**PQ810756**	**PQ811062**	**This study**
*P.plautus*	LE 212990	Russia	FJ774086	-	-	Justo et al. 2011b
*P.plautus*	HS18 2	Czech Republic	MH656435	-	-	Justo et al. 2011b
*P.plautus*	HS11	Czech Republic	MH656434	-	-	Justo et al. 2011b
*P.plautus*	OKA-349	Turkey	MG544916	-	-	Justo et al. 2011b
*P.plautus*	AJ203	Spain	HM562048	-	-	Justo et al. 2011b
*P.plautus*	UBCF23774	Canada	KC581304	-	-	Justo et al. 2011b
*P.pulcherrimus*	MCVE30061 T	Italy	MK446327	-	-	Kaygusuz et al. 2019
*P.pumae*	AJ824	USA	PP950714	-	PP950325	Justo et al. 2025
*P.pumae*	UC199304	USA	PP950715	-	PP950327	Justo et al. 2025
*P.pumae*	UBC-F26145 T	Canada	PP831609	-	-	Justo et al. 2025
*P.pumae*	UC1998535	USA	KF306003	-	PP950326	Justo et al. 2025
*P.punctatus*	JHC04 298	Sweden	MH656480	-	-	Ševcíková et al. 2020
*P.punctatus*	PRM 682743 T	Czech Republic	MH656438	-	-	Ševcíková et al. 2020
*P.readiarum*	PDD:103772	New Zealand	MN738644	-	-	Kaygusuz et al. 2019
*P.readiarum*	JAC11228	New Zealand	MN738638	-	-	Kaygusuz et al. 2019
*P.romellii*	FJAU 66558	China	OR994057	-	PP062827	Qi et al. 2024
*P.romellii*	FJAU 66559	China	OR994061	-	PP062828	Qi et al. 2024
*P.romellii*	FJAU66559	China	OR994061	-	PP062828	Qi et al. 2024
*P.romellii*	FJAU66558	China	OR994057	-	PP062827	Qi et al. 2024
*P.roseipes*	UC 1861251	USA	KC147681	-	-	Kaygusuz et al. 2019
*P.roseipes*	UC 1861249	USA	KC147679	-	-	Kaygusuz et al. 2019
*P.roseipes*	P27	USA	KF306003	-	-	Kaygusuz et al. 2019
*P.roseipes*	OKA-440	Turkey	MG544912	-	-	Kaygusuz et al. 2019
*P.roseipes*	OKA-488	Turkey	MG544913	-	-	Kaygusuz et al. 2019
*P.roseipes*	OKA-TR12	Turkey	MK123350	-	-	Kaygusuz et al. 2019
*P.saisamorniae*	BKF10213	Thailand	MN492646	-	-	Wannathes et al. 2022
*P.saisamorniae*	NW1567	Thailand	OM257410	-	-	Wannathes et al. 2022
*P.semibulbosus*	OkA-430	Turkey	MG544920	-	-	Kaygusuz et al. 2019
*P.semibulbosus*	LE 312914	Russia	KX216353	-	-	Kaygusuz et al. 2019
*P.semibulbosus*	GM2551	Spain	KR022023	-	-	Kaygusuz et al. 2019
*P.semibulbosus*	AJ870	Spain	KR022021	-	-	Kaygusuz et al. 2019
*Pluteus* sp.	UC 1861124	USA	KC147672	-	-	Kaygusuz et al. 2019
*Pluteus* sp.	M093671	USA	KR022009	-	-	Kaygusuz et al. 2019
*Pluteus* sp.	AJ470	USA	KR022028	-	-	Kaygusuz et al. 2019
*Pluteus* sp.	UC 1861124	USA	KC147672	-	-	Ševčíková et al. 2020
*Pluteus* sp.	MO93671	Mexico	KR022009	-	-	Ševčíková et al. 2020
***P.spaniophylluss***	**FJAU66593 T**	**China**	**PP516619**	**PP516669**	**PP551605**	**This study**
*P.subroseus*	LE F-347429 T	Vietnam	OQ732739	-	-	Malysheva et al. 2023
***P.tenuipileus***	**FJAU66591 T**	**China**	**PP516620**	**PP516670**	**PP551607**	**This study**
***P.tenuipileus***	**FJAU66592**	**China**	**PP516621**	**PP516671**	**PP551606**	**This study**
*P.thomensis*	SFSU:DED 8333 T	Sao Tome and Principe	MG968800	-	-	Desjardin and Perry 2018
*P.umbrosoides*	LE 312920	Russia	KX216349	-	-	Malysheva et al. 2016
*P.umbrosoides*	GDGM:29469	China	MH059512	-	-	Malysheva et al. 2016
*P.umbrosoides*	OKA-448	Turkey	MG544907	-	-	Malysheva et al. 2016
***P.umbrosoides***	**FJAU66615**	**China**	**PQ810780**	**PQ810741**		**This study**
*P.umbrosoides*	LE 312735 T	Russia	KX216321	-	-	Kaygusuz et al. 2021
*P.umbrosus*	12187	Italy	JF908622	-	-	Kaygusuz et al. 2019
*P.umbrosus*	LE 312838	Russia	KX216343	-	-	Kaygusuz et al. 2019
*P.umbrosus*	OKA-TR15	Turkey	MK123353	-	-	Kaygusuz et al. 2019
***P.umbrosus***	**FJAU66590**	**China**	**PP516604**	-	**PP928982**	**This study**
***P.ussuriensis***	**FJAU66579**	**China**	**PP516609**	**PP516659**	**PP551595**	**This study**
***P.ussuriensis***	**FJAU66578**	**China**	**PP516607**	**PP516658**	**PP551594**	**This study**
***P.ussuriensis***	**FJAU66577**	**China**	**PP516608**	**PP516657**	**PP551593**	**This study**
***P.ussuriensis***	**FJAU66576**	**China**	**PP516606**	**PP516656**	**PP551592**	**This study**
*P.ussuriensis*	LE F-312953 T	Russia	PP950739	-	PP950349	Justo et al. 2025
*P.variabilicolor*	LE 216873	Russia	FJ774077	-	-	Lezzi et al. 2014
*P.variabilicolor*	TNSF17602	Japan	HM562092	-	-	Lezzi et al. 2014
*P.variabilicolor*	OKA-TR13	Turkey	MK123351	-	-	Lezzi et al. 2014
*P.variabilicolor*	BP-FN 56936 T	Hungary	KP192912	-	-	Hosen et al. 2018
***P.variabilicolor***	**FJAU66583**	**China**	**PP516613**	**PP516663**	**PP551599**	**This study**
***P.variabilicolor***	**FJAU66588**	**China**	**PP516618**	**PP516668**	**PP551604**	**This study**
***P.variabilicolor***	**FJAU66584**	**China**	**PP516614**	**PP516664**	**PP551600**	**This study**
***P.variabilicolor***	**FJAU66585**	**China**	**PP516615**	**PP516665**	**PP551601**	**This study**
***P.variabilicolor***	**FJAU66586**	**China**	**PP516616**	**PP516666**	**PP551602**	**This study**
***P.variabilicolor***	**FJAU66587**	**China**	**PP516617**	**PP516667**	**PP551603**	**This study**
***P.variabilicolor***	**FJAU66623**	**China**	**PQ810763**	**PQ810740**	**PQ811063**	**This study**
*P.velutinus*	TNSF12372	Japan	HM562127	-	-	Ferisin and Dovana 2018
*P.velutinus*	LE 312915	Russia	KX216352	-	-	Ferisin and Dovana 2018
*P.velutinus*	LE 303693	Russia	KX216340	-	-	Ferisin and Dovana 2018
*P.velutinus*	FK1889	Brasile	KR022027	-	-	Ferisin and Dovana 2018
*P.velutinus*	K12851 T	India	JN603205	-	-	Ferisin and Dovana 2018
*P.velutinus*	MCVE29375	Italy	MG576118	-	-	Menolli et al. 2015a
*P.velutinus*	LE 312913	Russia	KX216351	-	-	Menolli et al. 2015a
*P.velutinus*	MCVE29376	Italy	MG574947	-	-	Menolli et al. 2015a
***P.velutinus***	**FJAU66619**	**China**	**PQ810764**	**PQ810742**	-	**This study**
***P.velutinus***	**FJAU66620**	**China**	**PQ810765**	**PQ810743**	-	**This study**
***P.ultraputripiceae***	**FJAU66594 T**	**China**	**PP516601**	**PP516653**	**PP551608**	**This study**
***P.ultraputripiceae***	**FJAU66595**	**China**	**PP516602**	**PP516654**	**PP551609**	**This study**
***P.ultraputripiceae***	**FJAU66611**	**China**	**PQ810760**	**PQ810737**	**PQ811047**	**This study**

**Figure 2.**
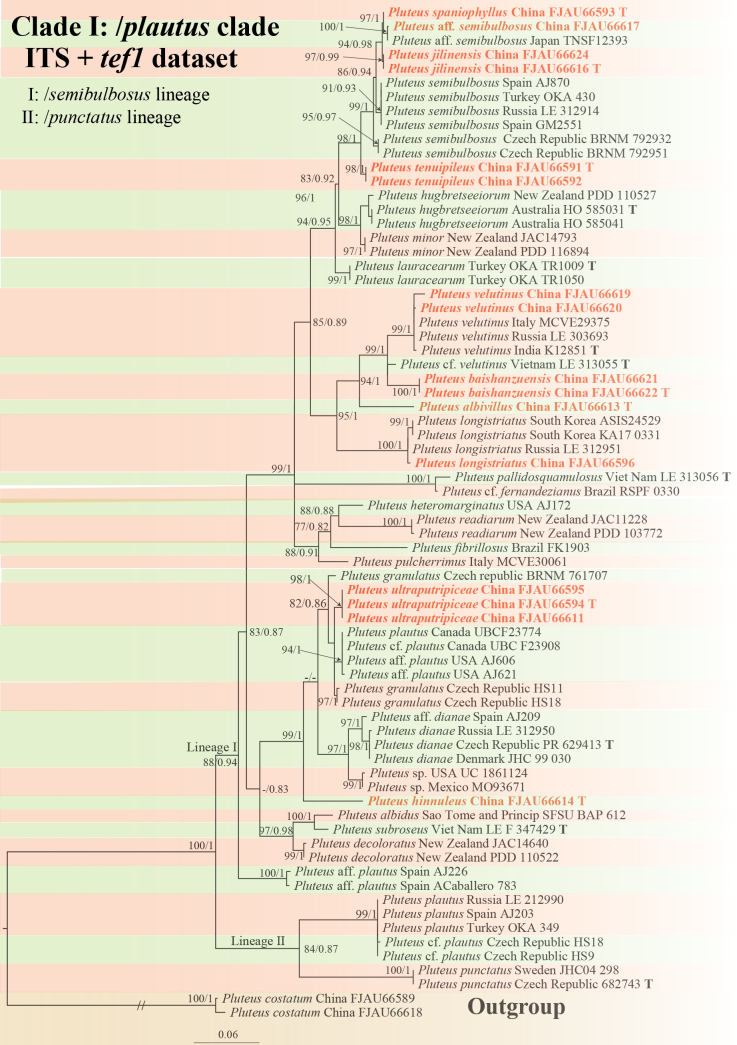
Phylogenetic tree of the /*plautus* clade of the sect.Hispidoderma. The best tree from the ML and BI analysis of the ITS+*tef1* dataset. The two values of internal nodes respectively represent the Maximum Likelihood bootstrap (MLBP)/Bayesian posterior probability (BIPP). Nodes with a bootstrap probability (BP) ≥ 80 and a Bayesian posterior probability (PP) ≥ 0.80 are indicated in the phylogram. This study species is in bold and red font.

**Figure 3.**
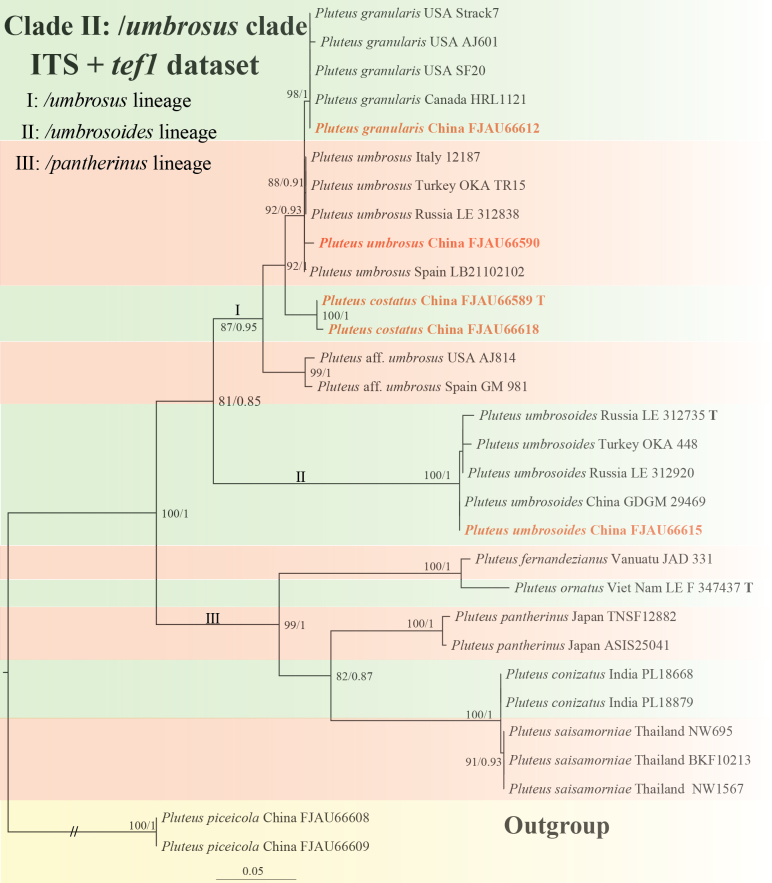
Phylogenetic tree of the /*umbrosus* clade and /*pantherinus* lineage of the sect.Hispidoderma. The best tree from the ML and BI analysis of the ITS+*tef1* dataset. The two values of internal nodes respectively represent the Maximum Likelihood bootstrap (MLBP)/Bayesian posterior probability (BIPP). Nodes with a bootstrap probability (BP) ≥ 80 and a Bayesian posterior probability (PP) ≥ 0.80 are indicated in the phylogram. This study species is in bold and red font.

**Figure 4.**
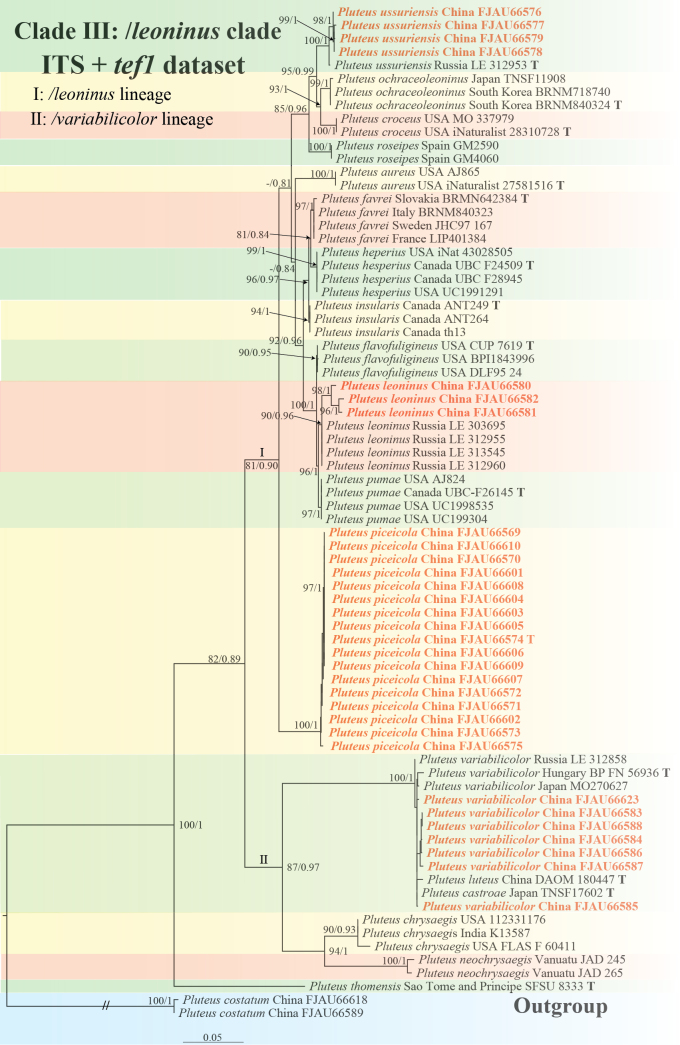
Phylogenetic tree of the /*leoninus* clade of the sect.Hispidoderma. The best tree from the ML and BI analysis of the ITS+*tef1* dataset. The two values of internal nodes respectively represent the Maximum Likelihood bootstrap (MLBP)/Bayesian posterior probability (BIPP). Nodes with a bootstrap probability (BP) ≥ 80 and a Bayesian posterior probability (PP) ≥ 0.80 are indicated in the phylogram. This study species is in bold and red font.

Pluteussect.Hispidoderma was first proposed by Fayod (1889) and subsequently further delimited by Singer (1958, 1986). The general topology of the phylogenetic tree constructed is consistent with the above-mentioned taxonomic and molecular concepts. In our study, species including *P.granularis*, *P.leoninus*, *P.longistriatus*, *P.umbrosoides*, *P.umbrosus*, *P.ussuriensis*, *P.variabilicolor* and *P.velutinus* have characteristics consistent with previous descriptions of sect.Hispidoderma by Singer (1958, 1986), Yang (2011), Xu (2016), Kaygusuz et al. (2019), Justo et al. (2025) (Figs 1–4).

According to previous studies (Singer 1986; Menolli et al. 2010, 2015a; Justo et al. 2011a, b; Pradeep et al. 2012; Lezzi et al. 2014; Malysheva et al. 2016; Ferisin and Dovana 2018; Kaygusuz et al. 2019), sect.Hispidoderma has been divided into three primary clades and one smaller lineage: the /*plautus* clade, the /*leoninus* clade, the /*umbrosus* clade and /*pantherinus* lineage. We retained the use of these three clades and one lineage in this study, as our LSU+ITS+*tef1* dataset-based phylogenetic tree supports this classification. Furthermore, the ITS+*tef1* combined dataset provided a higher resolution of species relationships within each of these evolutionary groups.

The /*plautus* clade contains two major lineages (Fig. 2):

The /*semibulbosus* lineage: Includes seven newly described species from China: *P.albivillus*, *P.baishanzuensis*, *P.hinnuleus*, *P.jilinensis*, *P.spaniophyllus*, *P.tenuipileus*, and *P.ultraputripiceae*. This lineage also contains previously known species: *P.semibulbosus* (P.aff.semibulbosus), *P.hubregtseorum*, *P.minor*, *P.lauracearum*, *P.velutinus* (P.cf.velutinus), *P.longistriatus*, P.cf.fernandezianus, *P.pallidosquamulosus*, *P.heteromarginatus*, *P.fibrillosus*, *P.readiarum*, *P.pulcherrimus*, *P.dianae* (P.aff.dianae), *Pluteus* sp., *P.granulatus*, *P.plautus* (P.cf.plautus, P.aff.plautus), *P.albidus*, *P.subroseus*, and *P.decoloratus*.

The /*punctatus* lineage: Includes *P.punctatus* and *P.plautus* (P.cf.plautus).

Separate analyses of ITS and *tef1* datasets revealed similar topologies, confirming the stability of these lineages. In the ITS phylogeny, *P.semibulbosus* and *P.plautus* in /*semibulbosus* lineage remained unresolved (Suppl. material 1: fig. S1). The *tef1* phylogeny, despite having fewer available sequences, maintained an overall topology consistent with the combined ITS+*tef1* dataset (Suppl. material 1: fig. S2).

The /*umbrosus* clade comprises two major lineages (Fig. 3):

The /*umbrosus* lineage: Includes the newly described *P.costatus* from China and the clade that we interpret to represent *P.umbrosus*, *P.granularis*, *P.umbrosoides*, and P.aff.umbrosus.

The /*umbrosoides* lineage: consists solely of *P.umbrosoides*.

Both ITS and *tef1* individual gene trees showed consistent branching patterns and topology with the combined dataset for this clade (Suppl. material 1: figs S3, S4).

The /*leoninus* clade is divided into two major lineages (Fig. 4):

The /*leoninus* lineage: Includes the newly described *P.piceicola* from China, and a clade consisting of *P.roseipes*, *P.leoninus* (P.aff.leoninus), *P.castroae*, *P.thomensis*, *P.aureus*, *P.croceus*, *P.favrei*, *P.flavofuligineus*, *P.hesperius*, *P.insularis*, *P.ochraceoleoninus*, *P.pumae*, and *P.ussuriensis*.

The /*variabilicolor* lineage: Includes *P.variabilicolor*, *P.chrysaegis*, and *P.neochrysaegis*.

Both ITS and *tef1* individual gene trees showed consistent branching patterns and topology with the combined dataset for this clade (Suppl. material 1: figs S5, S6).

Beyond the three main clades, we included sequences representing the /*pantherinus* lineage, comprising *P.fernandezianus*, *P.ornatus*, *P.saisamorniae*, *P.pantherinus*, and *P.conizatus*.

Both ITS and *tef1* phylogenies for this lineage were consistent with the combined dataset analysis (Suppl. material 1: figs S3, S4).

### ﻿Genetic distance estimation

We calculated genetic distances for 18 species of sect.Hispidoderma, including closely related and morphologically similar species. The genetic distance matrices ranged from 0.005 to 0.325 for ITS and from 0.002 to 0.297 for *tef1* (expressed as proportions, not percentages).

In the ITS dataset, the smallest genetic distances were observed between several species pairs: *P.flavofuligineus* and *P.insularis* (0.005, SE = 0.003), *P.flavofuligineus* and *P.leoninus* (0.005, SE = 0.003), and *P.granularis* and *P.umbrosus* (0.005, SE = 0.003). These low values, while indicating close relationships, still represent sufficient genetic differentiation when considered alongside morphological differences. The largest genetic distance was observed between *P.roseipes* and *P.velutinus* (0.325, SE = 0.037) (Suppl. material 1: table S1). In the *tef1* dataset, the smallest genetic distance was between *P.favrei* and *P.insularis* (0.002, SE = 0.002), while the largest was between *P.hinnuleus* and *P.roseipes* (0.297, SE = 0.037) (Suppl. material 1: table S2). The LSU region was not used for genetic distance calculations due to its highly conserved nature and limited number of available sequences, resulting in minimal variation among the studied species. The combined genetic distance analyses confirm that the nine newly described species are genetically distinct from previously described species of sect.Hispidoderma, corroborating their recognition as new taxa.

### ﻿Correlation between morphology and phylogenetics

Our morphological analyses revealed several distinctive features that correlate with the phylogenetic groupings (Fig. 1). We identified stable diagnostic characters for each clade, including pileus main color and size, lamellae edge coloration, stipe main color, basidiospore shape, pileipellis structure, presence of caulocystidia, and substrate preferences.

The /*plautus* clade exhibits considerable variation in pileus color and size, while consistently showing more subglobose to ellipsoid basidiospores. Species within this clade typically have hymenidermal to trichohymenidermal pileipellis, and most lack darkly pigmented lamellae edges. The /*umbrosus* clade is characterized by dark brown pileus with scales, distinctly dark-pigmented lamellae edges (except *P.granularis*), and consistent presence of caulocystidia. The pileipellis is predominantly trichohymeniderm with elongated elements. The /*leoninus* clade is distinguished by predominantly yellow-toned pileus and stipes, a variable presence of caulocystidia. The pileipellis structure in this clade is primarily hymenidermal or trichohymenidermal.

These morphological features, combined with substrate preferences and geographic distribution, provide robust diagnostic criteria for species identification within sect.Hispidoderma and support the phylogenetic framework established through molecular analyses. Detailed descriptions of all species, including the nine new taxa, are provided in the Taxonomy section.

### ﻿Taxonomy

#### ﻿/*plautus* clade

(Figs 5–17)

**Figure 5.**
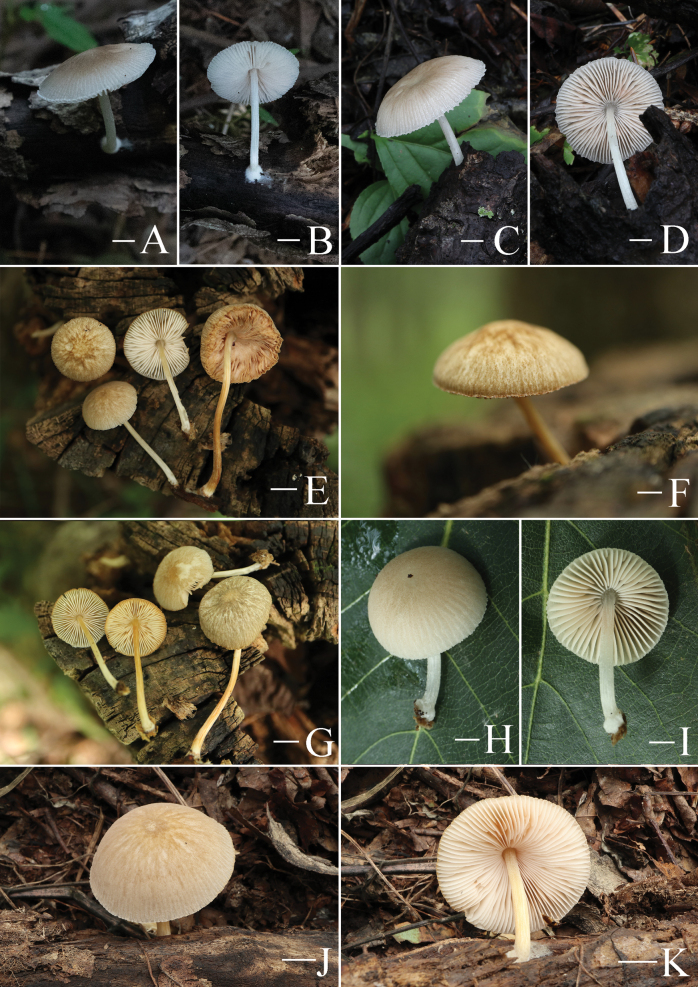
Basidiomata features. A–D. *Pluteustenuipileus* (A, B. FJAU66591, C, D. FJAU66592). E–G. *P.jilinensis* (FJAU66616). H–I. *P.spaniophyllus* (FJAU66593). J, K. P.aff.semibulbosus (FJAU66617). A–K. Photos by Gu Rao. Scale bars: 1 cm.

**Figure 6.**
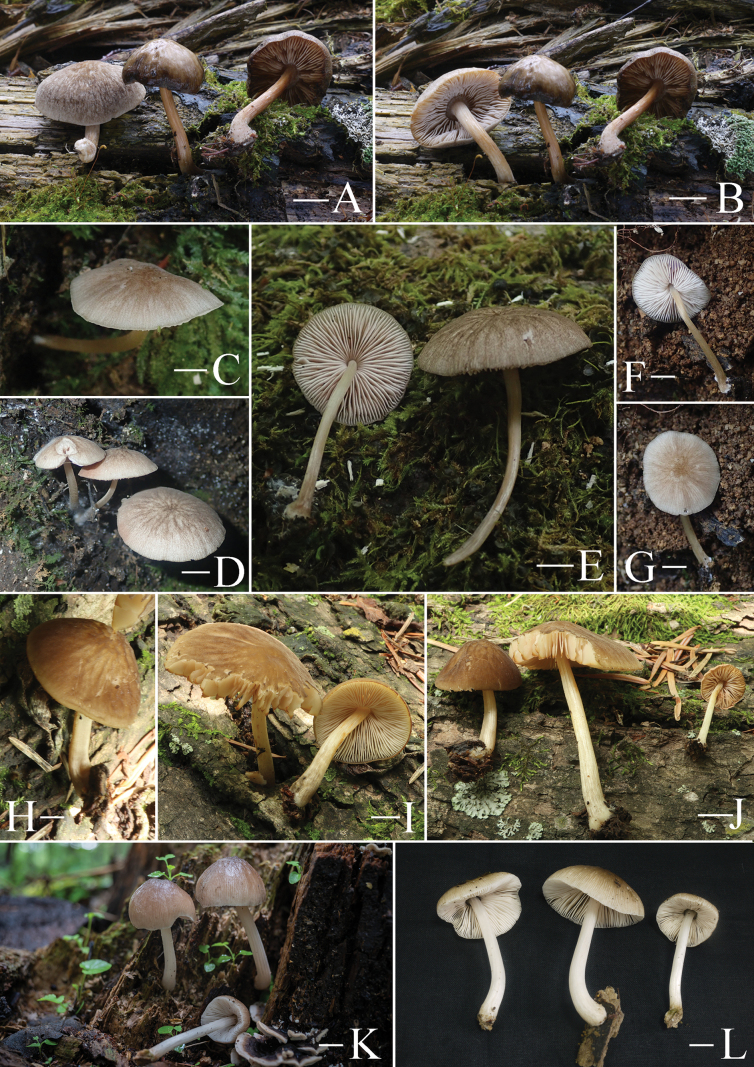
Basidiomata features. A, B. *Pluteusalbivillus* (FJAU66613). C–G. *P.baishanzuensis* (C, D. FJAU66621, E–G. FJAU66622). H–J. *P.velutinus* (H. FJAU66619, I, J. FJAU66620). K, L. *P.longistriatus* (FJAU66596). A, B, K, L. Photos by Zheng-Xiang Qi. C–G. Photos by Rui-Peng Liu. H–J. Photos by Gu Rao. Scale bars: 1 cm.

**Figure 7.**
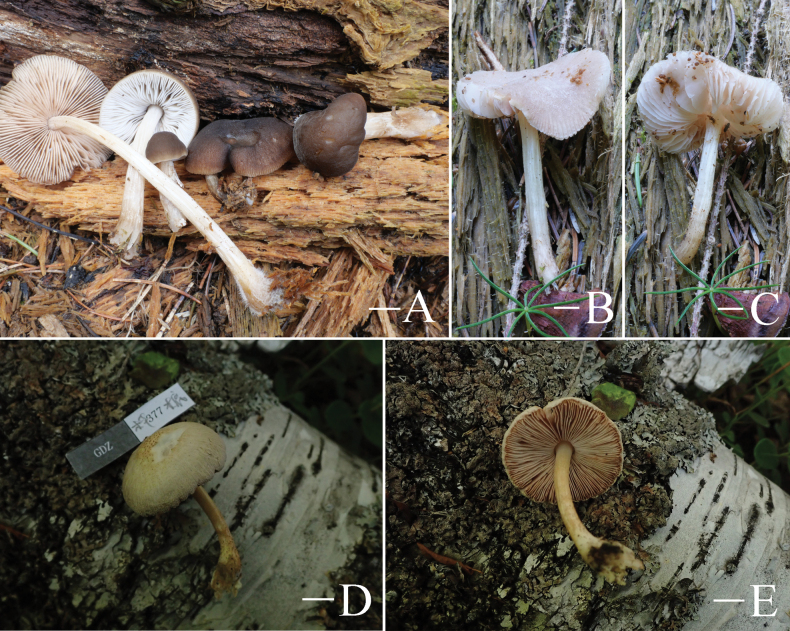
Basidiomata features. A–C. *Pluteusultraputripiceae* (A. FJAU66611, B, C. FJAU66594). D, E. *P.hinnuleus* (FJAU66614). A–C. Photos by Zheng-Xiang Qi. D, E. Photos by Di-Zhe Guo. Scale bars: 1 cm.

##### ﻿ Pluteus tenuipileus

Taxon classificationFungiAgaricalesPluteaceae

Z.X. QI, B. Zhang & Y. Li sp. nov.

174711C0-FCBB-5BF0-83A1-81F42DBFEA0D

MycoBank No: 853188

Figs 5A–D , 8

###### Etymology.

The species epithet “tenuipileus” (Latin) refers to the thin, flake-like pileus.

###### Diagnosis.

Morphologically similar to *P.lauracearum*, differing by its smooth and thin pileus, distinct ITS sequences (genetic distance = 0.021, SE = 0.006), and distribution in East Asia (China).

###### Holotype.

CHINA • Jilin Province, Cold Jungle National Nature Reserve; 11 August 2021, G. Rao, FJAU66591 (ITS: PP516621, LSU: PP516671, *tef1*: PP551607) (Collection no.: Rao 1325).

###### Description.

Basidiomata medium-sized. Pileus 17–33 mm diam; plano-convex, sometimes slightly depressed at the center; light grayish brown (2.5YR 7/2) with white margins (2.5YR 9/2); surface smooth to rarely white pruinose (2.5YR 9/2), radial striate from the middle to the margin; margin pilcate, with umbrella-like teeth. Lamellae cream to flesh-pink (2.5R 9/2–2.5R 9/6), free, crowded, thick, unequal, ventricose, 3–5 mm wide, with even edges; lamellar edge white. Stipe 20–25 × 2–3 mm, cylindrical, white (2.5R 9/2), base bulbous, fibrous and brittle bony, surface with white pruinose (2.5R 9/2). Odorless. Spore prints pink.

Basidiospores [120, 3, 2] 6.0–7.0 × 5.0–6.0(–6.5) μm, avL × avW = 6.4–6.6 × 5.5–5.7 µm, Q = 1.07–1.40 μm, avQ = 1.16–1.20 μm, subglobose to broadly ellipsoid or ovoid, slightly pinkish, smooth, thin-walled. Basidia 21–28 × 7–8 µm, clavate, thin-walled, 4-sterigmate, hyaline. Pleurocystidia 53–93 × 24–32 μm, abundant, scattered, fusiform or subfusiform, apical portion with mucronate, rostrate with up to 2–6 μm long, thin-walled, smooth, hyaline. Lamellar edge sterile. Cheilocystidia 36–68 × 14–26 μm, abundant, clustered, narrowly clavate to clavate, or long clavate, obtusely rounded apically, short basally, thin-walled, hyaline. Pileipellis a trichohymeniderm, with terminal elements 58–95 × 15–30 μm, broadly clavate or fusiform, thin-walled, with brown intracellular pigment. Stipitipellis a cutis, hyphae 3–12 µm diam, cylindrical, hyaline, thin-walled. Caulocystidia 24–53 × 7–17 μm, numerous, occurring in clusters, cylindrical to broadly clavate to broadly fusiform, or narrowly utriform, hyaline, thin-walled. Clamp connections absent in all tissues.

###### Habitat.

Solitary on decaying wood in coniferous forests.

###### World distribution.

China.

###### China distribution.

Jilin Province.

###### Additional specimens examined.

CHINA • Jilin Province, Cold Jungle National Nature Reserve; Solitary on rotting wood in mixed forests; 2 August 2020, G. Rao, FJAU66592 (Collection no.: Rao 927) (ITS: PP516620, LSU: PP516670, *tef1*: PP551606).

###### Notes.

*Pluteustenuipileus* is characterized by a pileus thinly, with a furrowed stripe along the margin. The fruiting bodies are brittle, and the pleurocystidia are apically mucronate or rostrate.

Morphologically, *P.tenuipileus* shares features with *P.lauracearum*, *P.boudieri*, and *P.semibulbosus*, but can be distinguished from each based on several characteristics. *P.tenuipileus* differs from *P.lauracearum* by its smooth pileus and smaller basidiospores (avL × avW = 6.4–6.6 × 5.5–5.7 µm), whereas *P.lauracearum* exhibits a pruinose or distinctly granulose pileus surface with larger basidiospores (avL × avW = 7.3 × 6.0 µm). Additionally, *P.tenuipileus* is distributed in East Asia (China), while *P.lauracearum* occurs in Eurasia (Turkey and Portugal) (Kaygusuz et al. 2019), with an ITS genetic distance of 0.021 (SE = 0.006). *P.tenuipileus* can be separated from *P.boudieri* by pileipellis structure: trichohymeniderm in the former versus monostratous hymeniderm in the latter (Vellinga and Schreurs 1985; Orton 1986). *P.semibulbosus* was originally described as a small whitish species characterized by a softly atomate, sulcate pileus and pubescent stipe with a distinctly bulbous base (Fries 1838). Lasch (in Fries 1838) did not provide basidioma dimensions, although Saccardo (1887: 674) documented a pileus 13 mm in width and stipe 25 mm in length. Despite numerous descriptions of this species in contemporary literature (Yang 2011; Xu 2016; Kaygusuz et al. 2019; Malysheva et al. 2023), the absence of corresponding type specimens and the lack of clear phylogenetic placement has necessitated the adoption of a broad concept of *P.semibulbosus*. Ševčíková (2020) and Justo et al. have indicated that this broad morphological species concept corresponds to a polyphyletic group (unpublished data) and that further studies, including the designation of a type specimen for *P.semibulbosus*, are required. Even within this broad conceptual framework, *P.semibulbosus* can be distinguished from *P.tenuipileus* primarily based on the quantity and morphology of pleurocystidia, while the exceptionally thin pileus serves as a more diagnostic feature of *P.tenuipileus* (Singer 1986).

When comparing *P.tenuipileus* with taxa that produce notably smaller basidiomata, such as *P.candidus* and *P.stylobates*, several distinctive morphological features become taxonomically significant. *P.tenuipileus* differs from *P.candidus* in having a wider pileus (17–33 mm) with radiating stripes, whereas *P.candidus* has smaller basidiomata (10 mm) lacking striations (Saccardo 1887). Similarly, *P.tenuipileus* can be differentiated from *P.stylobates* by its smooth to rarely white- pruinose surface, wider pileus (17–33 mm), and bulbous stipe base, in contrast to the smaller pileus (≤10–15 mm) with dark squamules on the surface and distinctive discoid stipe base characteristic of *P.stylobates* (Velenovský 1921).

**Figure 8.**
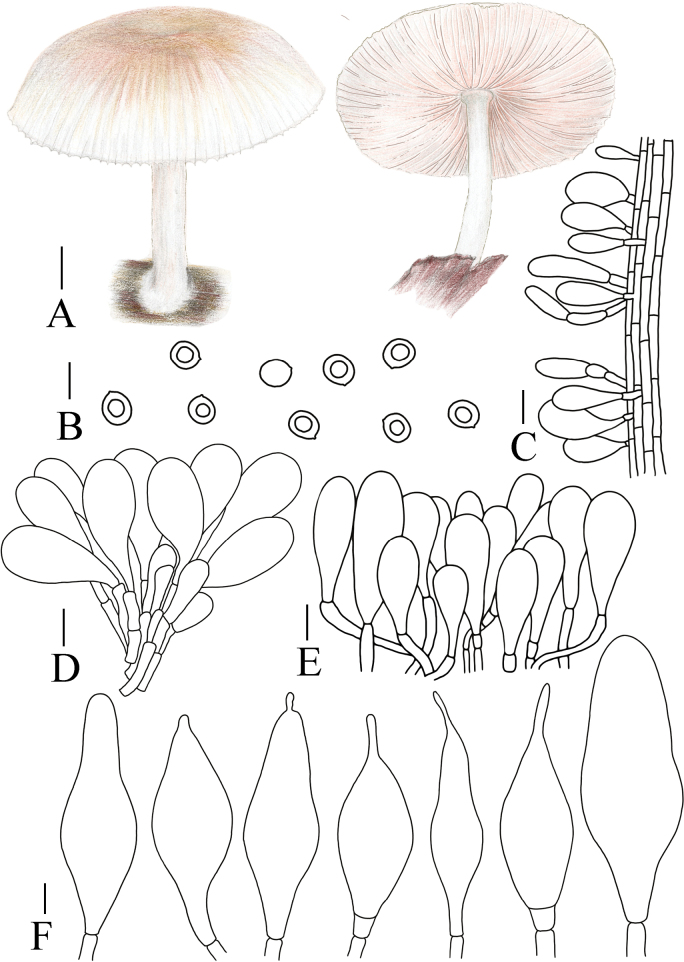
A. Macroscopic characteristics of *Pluteustenuipileus*. B. Basidiospores. C. Caulocystidia. D. Cheilocystidia. E. Pileipellis elements. F. Pleurocystidia. Scale bars: 0.5 cm (A); 10 µm (B, F); 20 µm (C, D, E).

##### ﻿ Pluteus spaniophyllus

Taxon classificationFungiAgaricalesPluteaceae

Z.X. QI, B. Zhang & Y. Li sp. nov.

B9E5F9BF-D43B-59F8-986A-3CE5B4CC9C66

MycoBank No: 857275

Figs 5H, I , 9

###### Etymology.

The species epithet “spaniophyllus” (Latin) refers to the sparseness of the lamellae.

###### Diagnosis.

*Pluteusspaniophyllus* differs from *P.jilinensis* by its velvety pileus, sparse lamellae, smaller basidiospores, and their ITS genetic distance is 0.008 (SE = 0.004) and *tef1* genetic distance is 0.008 (SE = 0.004).

###### Holotype.

CHINA • Jilin Province, Cold Jungle National Nature Reserve; Solitary on rotting wood in broad-leaved forests; 7 August 2021, G. Rao, FJAU66593 (Collection no.: Rao 1292) (ITS: PP516619, LSU: PP516669, *tef1*: PP551605).

###### Description.

Basidiomata small-sized. Pileus 17 mm diam; hemispherical; white to light yellow (2.5Y 9/10), slightly with brown velvety or pruinose on the surface of the center (7.5YR 8/6); margin radial translucently striate. Lamellae pale flesh–pink (5.0R 9/6), free, relatively sparse, thick, unequal, slightly ventricose, 4–7 mm wide, with even edges; lamellar edge white. Stipe 14 × 3 mm, cylindrical, with a bulbous base, fibrous, surface with white pruinose (2.5Y 9/12). Odorless. Spore prints pink.

Basidiospores [60, 1, 1] (–6.5)7.0–7.5(–8.0) × 6.0–6.5(–7.0) μm, avL × avW = 7.0–7.3 × 6.2–6.5 µm, Q = 1.00–1.33 μm, avQ = 1.07–1.10 μm, globose, subglobose to broadly ellipsoid, pale pink, smooth, thin-walled. Basidia 23–30 × 8–11 μm, clavate to broadly clavate, thin-walled, 4-sterigmate, hyaline. Pleurocystidia 53–91 × 18–31 μm, scattered, fusiform to subfusiform, apically obtuse, thin-walled, smooth, hyaline. Lamellar edge sterile. Cheilocystidia 25–70 × 11–27 μm, abundant, clustered, similar in form to pleurocystidia, narrowly to broadly fusiform, broadly subfusiform, some apical with small irregular horns or mucronate, thin-walled, hyaline. Pileipellis a trichohymeniderm, with terminal elements 51–92 × 12–28 μm, broadly clavate or fusiform, thin-walled, with brown intracellular pigment. Stipitipellis a cutis, hyphae 3–12 µm diam, cylindrical, hyaline, thin-walled. Caulocystidia 28–47 × 9–18 μm, numerous, occurring in clusters, cylindrical to broadly clavate to broadly fusiform, or narrowly utriform, hyaline, thin-walled. Clamp connections absent in all tissues.

###### Habitat.

Solitary on rotting wood in broad-leaved forests.

###### World distribution.

China.

###### China distribution.

Jilin Province.

###### Notes.

*Pluteusspaniophyllus* is characterized by its small basidiomata, hemispherical pileus ranging from white to light yellow, with brown velvety on the surface, margin radial translucently striate, sparse lamellae, and a white pruinose on the stipe base, which is inflated and nearly bulbous.

Morphologically, *P.spaniophyllus* shares greatest similarity with *P.hubreg­tseorum* and *P.boudieri*, but can be distinguished from each by several key features. *P.spaniophyllus* differs from *P.hubregtseorum* by its fleshy brown pileus, in contrast to the yellow-to-brownish-gold pileus of the latter. Additionally, *P.spaniophyllus* produces pale flesh-pink and sparsely arranged lamellae, whereas those of *P.hubregtseorum* are pale pink to flesh pink and crowded. The species also differ in their geographic distribution, with *P.spaniophyllus* occurring in East Asia (China) and *P.hubregtseorum* in Australia (Ševcíková et al. 2021), further supported by an ITS genetic distance of 0.023 (SE = 0.006).

*P.spaniophyllus* bears a closer resemblance to the white-capped *P.boudieri*, from which it is primarily distinguished by pileipellis morphology. *P.boudieri* exhibits thin, filamentous, light-brown pileipellis hyphae with generally cylindrical or subfusiform terminal elements (Vellinga and Schreurs 1985; Orton 1986). In comparison with *P.atriavellaneus*, *P.spaniophyllus* has a white to light yellow pileus, grows on decaying wood, and is distributed in East Asia (China), whereas *P.atriavellaneus* is characterized by a dark fuliginous to avellaneous, hygrophanous and finely pubescent pileus, growth on humus, and distribution in North America (USA) (Murrill 1917). Finally, *P.spaniophyllus* is distinguished from *P.avellaneus* by its central light yellow pileus with white margins and radial striate (distributed in East Asia, China), in contrast to the centrally paler, hygrophanous pileus with non-striped margins of *P.avellaneus* (distributed in North America, USA) (Murrill 1917).

In the phylogenetic analysis, *P.spaniophyllus* (specimen FJAU66593 from China) is strongly supported as a sister taxon to P.aff.semibulbosus (MLB = 98, BPP = 1, Fig. 2). These species are further distinguished by several morphological features: *P.spaniophyllus* produces smaller basidiomata (17 mm) with sparser lamellae and cheilocystidia bearing small irregular apical horns, whereas P.aff.semibulbosus forms larger basidiomata (43 mm) with denser lamellae and cheilocystidia lacking irregular apical horns (Table 3). Molecular evidence further supports their separation, with an ITS genetic distance of 0.009 (SE = 0.004) and *tef1* genetic distance of 0.004 (SE = 0.003).

**Figure 9.**
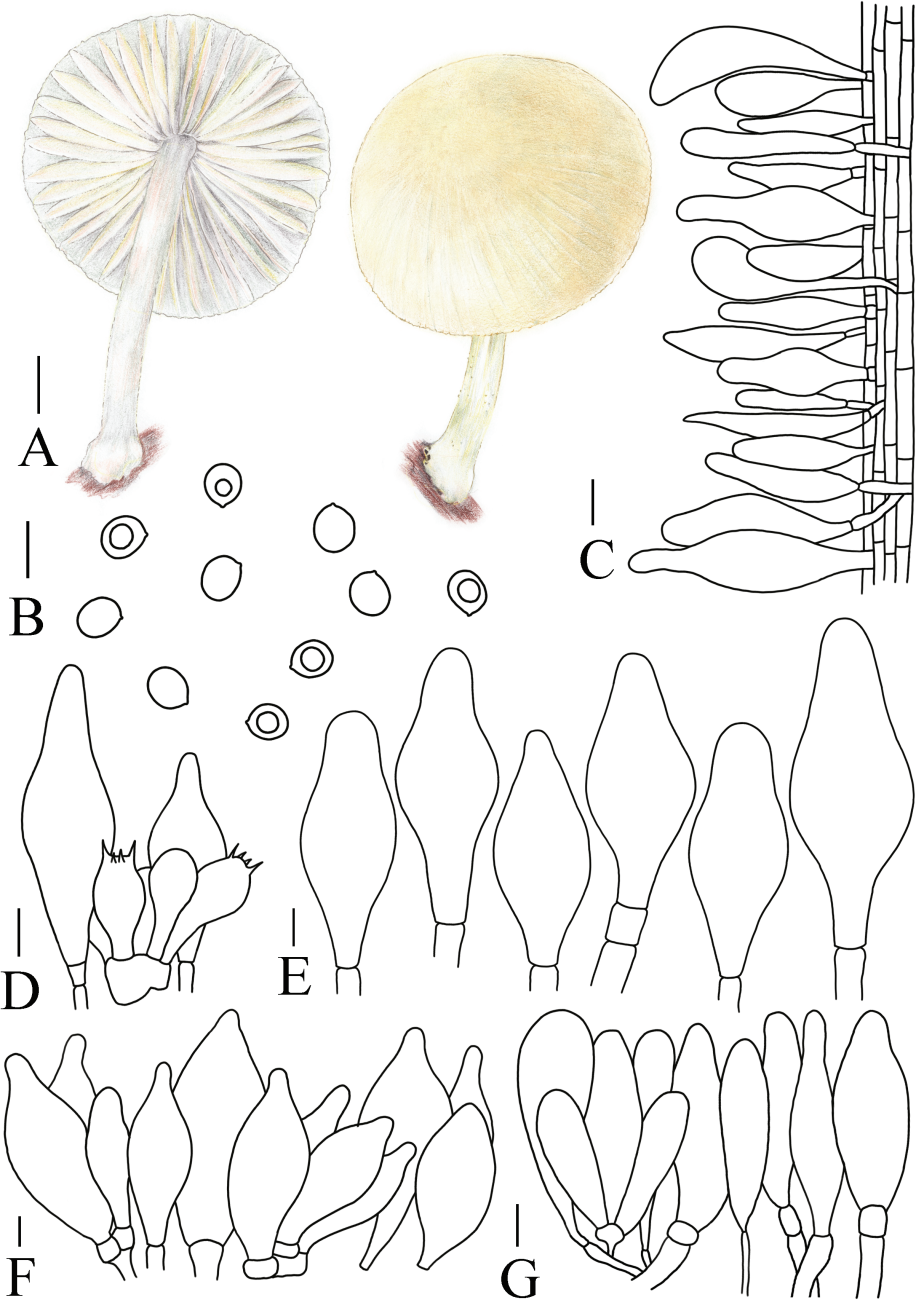
A. Macroscopic characteristics of *Pluteusspaniophyllus*. B. Basidiospores. C. Caulocystidia. D. Basidia and Pleurocystidia. E. Pleurocystidia. F. Cheilocystidia. G. Pileipellis elements. Scale bars: 0.5 cm (A); 10 µm (B–F); 20 µm (G).

**Table 3.** Diagnostic characters for *Pluteusspaniophyllus*, *P.jilinensis* and P.aff.semibulbosus.

Character	*Pluteusspaniophyllus*	*Pluteusjilinensis*	Pluteusaff.semibulbosus
Pileus main color	light yellow	burnt yellow to gray-brown	charred yellow to pale yellow
Pileus surface	velvety	squamules	velvety
Pileus diam	17 mm	15–21 mm	43 mm
Pileus margin striate	translucently striate	no striate	no striate
Lamellar	relatively sparse	crowded	crowded
Stipe main color	white	brown	pale yellow
Stipe base	bulbous base	slightly bulbous base	slightly bulbous base
Basidiospores shape	globose, subglobose to broadly ellipsoid	subglobose to broadly ellipsoid	globose to subglobose, rarely broadly ellipsoid
Basidiospores size (avl × avw)	7.0–7.3 × 6.2–6.5 µm	7.5–7.8 × 6.4–6.8 µm	7.4–7.6 × 6.2–6.5 µm
The shape of pileipellis elements	broadly clavate or fusiform	subcylindric or subfusiform	broadly subcylindric or subfusiform
The shape of caulocystidia	cylindrical, broadly clavate, broadly fusiform, or narrowly utriform	vesicles, subfusiform	clavate, fusiform, or broadly fusiform

##### ﻿ Pluteus jilinensis

Taxon classificationFungiAgaricalesPluteaceae

Z.X. QI, B. Zhang & Y. Li sp. nov.

DDE8ACF8-2F01-5C81-8DA2-5E92F33098C6

MycoBank No: 857276

Figs 5E–G , 10

###### Etymology.

jilin, for the geographic origin of the type collection.

###### Diagnosis.

*Pluteusjilinensis* is separated from *P.spaniophyllus* by its burnt yellow pileus, with distinct squamules around the center, dense lamellae, and their ITS genetic distance is 0.008 (SE = 0.004), *tef1* genetic distance is 0.008 (SE = 0.004).

###### Holotype.

CHINA • Jilin Province, Cold Onion Ridge Forest Park; Latitude and longitude: 43°02'2.06"N, 127°58'44.69"E; Scattered on very rotten decaying wood in broad-leaved forests (*Q.mongolica*); 9 August 2019, G. Rao, FJAU66616 (Collection no.: Rao 1314) (ITS: PQ810767, LSU: PQ810745, *tef1*: PQ811051).

###### Description.

Basidiomata small-sized. Pileus 15–21 mm diam; convex or plano-convex, often with a low, broad umbo; burnt yellow (5YR 8/6) to gray-brown (10YR 8/2), transitioning to light brown (2.5Y 8/2) toward the margin; surface rough, with distinct squamules or rugose-venose around the center; margin crenulate, with small rounded or blunt teeth. Context yellowish (2.5Y 9/2). Lamellae free, 3–5 mm wide, crowded, initially white (2.5YR 9/2), pink at maturity (2.5R 9/6), unequal, moderately thick, ventricose, edges even; lamellar edge concolorous to the sides. Stipe 21–45 × 2–4 mm, hollow, fibrous, clavate, slightly expanded at the base, transparent to whitish (7.5RP 9/2) when young, light brown (5YR 5/2) to brown (7.5YR 5/4) at maturity. Spore print unknown.

Basidiospores [60/5/2] 7.0–8.0(–8.5) × 6.0–7.0(–7.5) µm, avL × avW = 7.5–7.8 × 6.4–6.8 µm, Q = 1.07–1.25 µm, avQ = 1.15–1.20 µm, subglobose to broadly ellipsoid, smooth, slightly pinkish, thin-walled. Basidia 20–25 × 12–18 µm, broadly clavate, thin-walled, 4-sterigmate, and hyaline. Pleurocystidia 55–83 × 15–23 μm, rare, scattered, fusiform to broadly fusiform, apically obtuse, thin-walled, smooth, partly containing brown intracellular pigment. Lamellar edge sterile. Cheilocystidia 37–63 × 16–21 μm, abundant, clustered, clavate, subfusiform to broadly fusiform, apically obtuse, with or without mucronate, thin-walled, smooth, hyaline. Pileipellis a hymeniderm, with terminal elements 75–98 × 17–28 μm, subcylindric or subfusiform, some apices with finger-like projections, thin-walled, with brown intracellular pigment. Stipitipellis a cutis, hyphae 5–8 μm diam, cylindrical, hyaline, thin-walled. Caulocystidia 36–55 × 13–19 μm, clustered, more numerous, composed of vesicles, subfusiform, bluntly rounded apically, partly containing brownish intracellular pigment, smooth, thin-walled. Clamp connections absent in all tissues.

###### Habitat.

Scattered on very rotten decaying wood in broad-leaved forests (*Q.mongolica*).

###### World distribution.

China.

###### China distribution.

Jilin Province.

###### Additional specimens examined.

CHINA • Jilin Province, Quanyang Township, Beigang; 43°02'2.06"N, 127°58'44.69"E; Scattered on very rotten decaying wood in broad-leaved forests (*Q.mongolica*); 23 August 2021, Z.X. Qi, FJAU66624 (Collection no.: Qi 401) (ITS: PQ814290, LSU: PQ814291).

###### Notes.

*Pluteusjilinensis* is characterized by its smaller basidiomata, burnt yellow pileus, and pleurocystidia with bluntly rounded apices.

Morphologically, *P.jilinensis* may be confused with *P.spaniophyllus* and *P.depauperatus*. *P.jilinensis* is distinguished from *P.spaniophyllus* by its denser lamellae and fibrous, harder stipe, in contrast to the sparser lamellae and crumbly, fragile stipe of *P.spaniophyllus* (Table 3). This morphological distinction is further supported by molecular data, with an ITS genetic distance of 0.008 (SE = 0.004) and *tef1* genetic distance of 0.008 (SE = 0.004) between these taxa. *P.jilinensis* differs from *P.depauperatus* in several respects: *P.jilinensis* produces smaller basidiomata (pileus 15–21 mm), with a predominantly burnt yellow, unstriped pileus having a rough surface, and larger basidiospores (avL × avW = 7.5–7.8 × 6.4–6.8 µm). In contrast, *P.depauperatus* exhibits larger basidiomata (pileus 18–50 mm) with predominantly brown coloration (pale brown, grey-brown, or saffron-brown), distinctly striped margins, sometimes granular center, smaller basidiospores (avL × avW = 6.33 × 5.43 µm), and a distinctive odor (Kühner and Romagnesi 1956).

Phylogenetically, *P.jilinensis* and P.aff.semibulbosus form strongly supported sisters. Although both species share similarly sized basidiospores, fusiform pleurocystidia, and subfusiform cheilocystidia, they can be differentiated by several features: *P.jilinensis* produces smaller basidiomata (≤21 mm in diameter) with a more spreading pileus and pileipellis composed of clavate and cylindrical elements, while P.aff.semibulbosus forms larger basidiomata (up to 43 mm in diameter) with a hemispherical pileus and pileipellis consisting of inflated clavate cells. Additionally, the caulocystidia in *P.jilinensis* have bluntly rounded apices, whereas those in P.aff.semibulbosus possess a papillate apical projection (Table 3). These morphological distinctions are reinforced by genetic distances of 0.013 (SE = 0.007) for ITS and 0.004 (SE = 0.003) for *tef1*.

**Figure 10.**
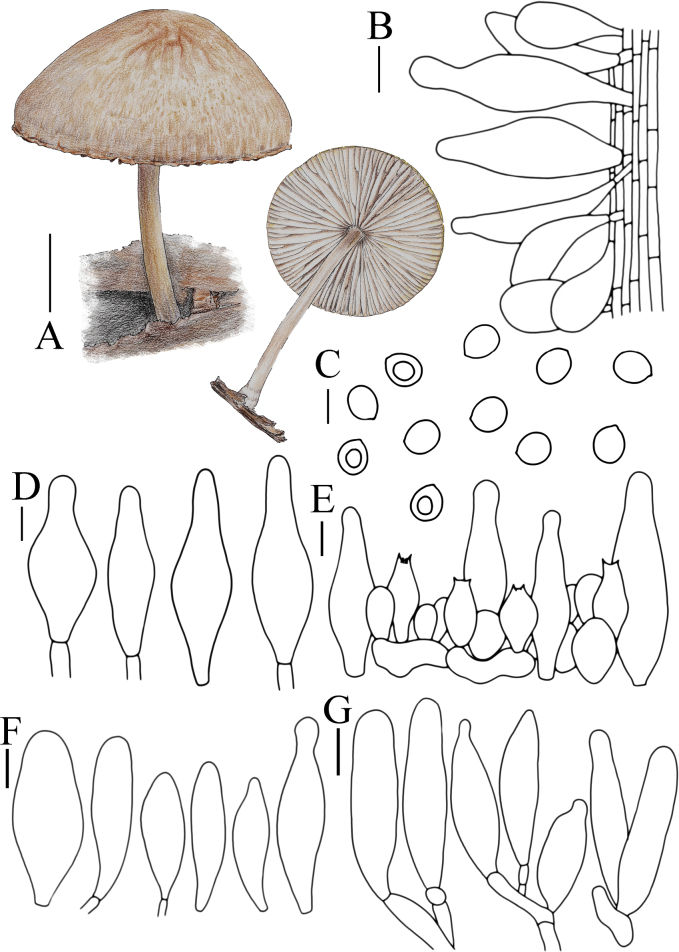
A. Macroscopic characteristics of *Pluteusjilinensis*. B. Caulocystidia. C. Basidiospores. D. Pleurocystidia. E. Basidia and Pleurocystidia. F. Cheilocystidia. G. Pileipellis elements. Scale bars: 1 cm (A); 5 µm (C); 10 µm (B, D–F); 20 µm (G).

##### ﻿ Pluteus aff. semibulbosus

Taxon classificationFungiAgaricalesPluteaceae

AE2047D2-58B6-5C19-B2D8-5A8C69EA7448

Figs 5J, K , 11

###### Description.

Basidiomata medium-sized. Pileus 43 mm diam; hemispherical; charred yellow in the center, transitioning to light yellow (10YR 9/2) to white (10P 9/2) toward the margin; surface dry, or dehisce, slightly velvety; margin shorter grooves striate, and straight. Context pale yellow (2.5Y 9/2). Lamellae free, 4–8 mm wide, crowded, white (10P 9/2) to yellowish (2.5Y 9/2), unequal, thin, ventricose, edges even or flocculose; lamellar edge white. Stipe 39 × 4 mm, fibrous, hollow, clavate, slightly curved at the base and inflated bulbous, with white mycelium close to the rotting wood, overall pale yellow (10YR 9/2) with white (10P 9/2) glandular dots on the surface. Spore print unknown.

Basidiospores [40/1/1] 7.0–8.0(–8.5) × 6.0–6.5(–7.5) µm, avL × avW = 7.4–7.6 × 6.2–6.5 µm, Q = 1.07–1.21 µm, avQ = 1.10–1.15 µm, globose to subglobose, rarely broadly ellipsoid, smooth, slightly pinkish, thin-walled. Basidia 24–33 × 9–11 µm, clavate, thin-walled, 4-sterigmate, hyaline. Pleurocystidia 53–80 × 15–35 μm, rare, scattered, fusiform to broadly subfusiform, or broadly clavate, apically obtuse, thin-walled, smooth, hyaline. Lamellar edge sterile. Cheilocystidia 33–61 × 12–18 μm, abundant, clustered, clavate or subfusiform, apically obtuse, thin-walled, hyaline. Pileipellis a hymeniderm, with terminal elements 70–105 × 18–29 μm, broadly subcylindric or subfusiform, thin-walled, with pale brown intracellular pigment. Stipitipellis a cutis, hyphae 4–6 μm diam, cylindrical, hyaline, thin-walled. Caulocystidia 43–73 × 12–22 μm, scattered to clustered, abundant, composed of clavate, fusiform, and broadly fusiform, most with bluntly rounded apices, a few with small apical projections, some containing brownish intracellular pigment, thin-walled, smooth. Clamp connections absent in all tissues.

###### Habitat.

Solitary on very rotten decaying wood in broad-leaved forests (*Q.mongolica*).

###### World distribution.

China.

###### China distribution.

Jilin Province.

###### Additional specimens examined.

CHINA • Jilin Province, Cold Onion Ridge Forest Park; 43°02'2.09"N, 127°58'77.59"E; Scattered on very rotten decaying wood in broad-leaved forests (*Q.mongolica*); 5 August 2021, G. Rao, FJAU66617 (Collection no.: Rao 1285) (ITS: PQ810766, LSU: PQ810744, *tef1*: PQ811050).

###### Notes.

The Chinese specimens described here share morphological similarities with *P.semibulbosus* sensu lato, including the bulbous stipe base, subglobose spores, and fusiform pleurocystidia. However, *P.semibulbosus* lacks a clear taxonomic delimitation, as noted by Ševčíková (2020) and Justo et al. (unpublished), who indicated that this taxon represents a polyphyletic group requiring further study.

In our phylogenetic analysis, the Chinese specimens formed a strongly supported clade (MLB = 100, BPP = 1, Fig. 2) with the Japanese specimen TNSF12393 (labeled as P.aff.semibulbosus). Although TNSF12393 lacks morphological description, the molecular evidence suggests these specimens represent the same taxonomic entity. As shown in Table 3, our material can be distinguished from the closely related *P.jilinensis* and *P.spaniophyllus* by its larger basidiomata (pileus 43 mm), hemispherical pileus shape, and distinctive caulocystidia with small apical projections.

Given the uncertain status of *P.semibulbosus* sensu lato and pending a comprehensive revision of this species complex, we provisionally treat our specimens as P.aff.semibulbosus until further taxonomic resolution is achieved.

**Figure 11.**
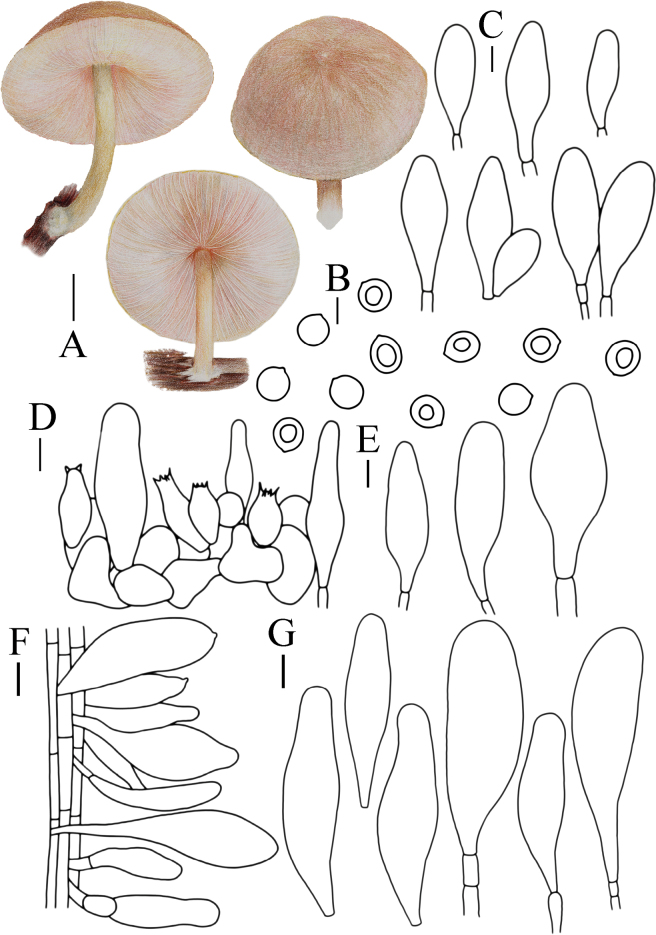
A. Macroscopic characteristics of Pluteusaff.semibulbosus. B. Basidiospores. C. Cheilocystidia. D. Basidia and Pleurocystidia. E. Pleurocystidia. F. Caulocystidia. G. Pileipellis elements. Scale bars: 1 cm (A); 5 µm (B); 10 µm (C–G).

##### ﻿ Pluteus ultraputripiceae

Taxon classificationFungiAgaricalesPluteaceae

Z.X. QI, B. Zhang & Y. Li sp. nov.

8F3C0926-20B7-5652-B691-8F3DE7084A52

MycoBank No: 853189

Figs 7A–C , 12

###### Etymology.

Derived from Latin ‘ultra’ (extremely), ‘putri’ (rotten), and ‘piceae’ (of spruce), the species epithet “ultraputripiceae” characterizes the fungus’s distinctive ecological niche on severely decomposed spruce wood substrates.

###### Diagnosis.

*Pluteusultraputripiceae* is characterized by its brown to light flesh-brown pileus, broadly ellipsoid to ellipsoid basidiospores (avL × avW = 7.7–8.0 × 6.4–6.8 µm), pleurocystidia lacking apical excrescences. It differs from *P.dianae* by the absence of small apical excrescence structures at the top of pleurocystidia, substrate preference for highly decayed trunks, and distribution in East Asia (China). The ITS genetic distance is 0.040 (SE = 0.008).

###### Holotype.

CHINA • Xinjiang Uygur Autonomous Region, Ili Kazakh Autonomous Prefecture, Tekes County, Jongkushtai village, 42°91'78.66"N, 82°12'56.22"E, alt. 2175 m, 10 July 2023, Z.X. Qi, FJAU66594 (ITS: PP516601, LSU: PP516653, *tef1*: PP551608) (Collection no.: Qi 1324)

###### Description.

Basidiomata medium-sized. Pileus 27–47 mm diam; convex to slightly hemispherical when young, brown to dark brown (7.5YR 5/4-10YR 4/6); plano-convex to applanate at maturity, light flesh–brown (2.5YR 9/6) to cinnamon-colored (2.5YR 8/4) and smooth, with slightly translucently striate at the margin, crenulate. Lamellae creamy white (2.5YR 8/6), free, crowded, thick, unequal, slightly ventricose, 6–9 mm wide, edges even; lamellar edge white. Stipe 31–55 × 5–7 mm, cylindrical, fibrous, with white longitudinal striate on the surface (2.5YR 9/4). Odorless. Spore prints pink.

Basidiospores [100, 7, 3] 7.0–8.0(–8.5) × 6.0–7.0(–7.5) μm, avL × avW = 7.7–8.0 × 6.4–6.8 µm, Q = 1.10–1.41 μm, avQ = 1.14–1.20 μm, broadly ellipsoid to ellipsoid, or ovoid, pale pink, smooth, thin-walled. Basidia 21–29 × 6–10 µm, clavate, thin-walled, 4-sterigmate, hyaline. Pleurocystidia 54–102 × 20–36 μm, few, scattered, clavate to broadly clavate, or fusiform, apically obtusely rounded, thin-walled, smooth, hyaline. Lamellar edge sterile. Cheilocystidia 41–79 × 18–29 μm, abundant, clustered, fusiform to narrowly clavate to clavate, or broadly clavate, bluntly rounded apically, thin-walled, smooth, hyaline. Pileipellis a trichohymeniderm, with terminal elements 35–64 × 11–25 μm broadly clavate or fusiform, thin-walled, with brown intracellular pigment. Stipitipellis a cutis, hyphae 3–12 µm diam, cylindrical, hyaline, thin-walled. Caulocystidia 30–61 × 10–21 μm, numerous, occurring in clusters, cylindrical to broadly clavate to broadly fusiform, or narrowly utriform, apically obtusely rounded, smooth, thin-walled, with brown intracellular pigment. Clamp connections absent in all tissues.

###### Habitat.

Solitary to scattered on highly decayed trunks in spruce forests (*P.schrenkiana*).

###### World distribution.

China.

###### China distribution.

Xinjiang Uygur Autonomous Region.

###### Additional specimens examined.

CHINA • Xinjiang Uygur Autonomous Region, Ili Kazakh Autonomous Prefecture, Tekes County, Jongkushtai village, solitary on rotting wood in spruce forests (*P.schrenkiana*), 42°17'46.35"N, 82°77'54.24"E, alt. 2055 m, 13 July 2023, Z.X. Qi, FJAU66595 (Collection no.: Qi 1329) (ITS: PP516602, LSU: PP516654, *tef1*: PP551609). CHINA • Xinjiang Uygur Autonomous Region, Ili Kazakh Autonomous Prefecture, Tekes County, Jongkushtai village. Scattered on rotting wood in spruce forests (*P.schrenkiana*). 42°97'26.51"N, 82°12'27.25"E, alt. 2192 m, 30 August 2024, Z.X. Qi, FJAU66611 (Collection no.: Qi 4887) (ITS: PQ810760, LSU: PQ810737, *tef1*: PQ811047).

###### Notes.

*Pluteusultraputripiceae* is distinguished by its dark brown to pinkish-brown pileus, relatively large basidiospores, and caulocystidia with brown intracellular pigment.

Morphologically, *P.ultraputripiceae* shares similarities with *P.dianae* and *P.plautus* but can be differentiated based on several key features. *P.ultraputripiceae* is distinguished from *P.dianae* by its larger basidiospores (avL × avW = 7.7–8.0 × 6.4–6.8 µm), pleurocystidia lacking excrescences, substrate preference for highly decayed trunks (*Piceaschrenkiana*), and distribution in East Asia (China). In contrast, *P.dianae* produces smaller basidiospores (avL × avW = 7.2 × 5.5 µm), pleurocystidia with excrescences, and primarily inhabits decayed broadleaf trunks in Central and Eastern Europe (Czechia, Denmark, and Russia) (Ševčíková et al. 2020). This distinction is further supported by an ITS genetic distance of 0.040 (SE = 0.008) between these taxa. *P.ultraputripiceae* is separated from *P.plautus* primarily by basidiomata coloration: *P.ultraputripiceae* exhibits a dark brown to pinkish-brown pileus with a white stipe, whereas *P.plautus* features an alutaceous to fuligineous pileus and a velvety brown to blackish-brown stipe with similarly pigmented context (Weinmann 1836).

The phylogenetic analysis strongly supports the monophyly of *P.ultraputripiceae*, with three specimens forming a well-supported (MLB = 98, BPP = 1, Fig. 2) sister to *P.granulatus*. These species can be differentiated by basidiomata morphology and geographic distribution: *P.ultraputripiceae* possesses a light flesh-brown to cinnamon-colored, smooth pileus and occurs in East Asia (China), while *P.granulatus* is characterized by a pileus with reddish tinge and distinct granules, occurring in Europe (Czechia and Italy). The ITS genetic distance between these taxa is 0.008 (SE = 0.003).

**Figure 12.**
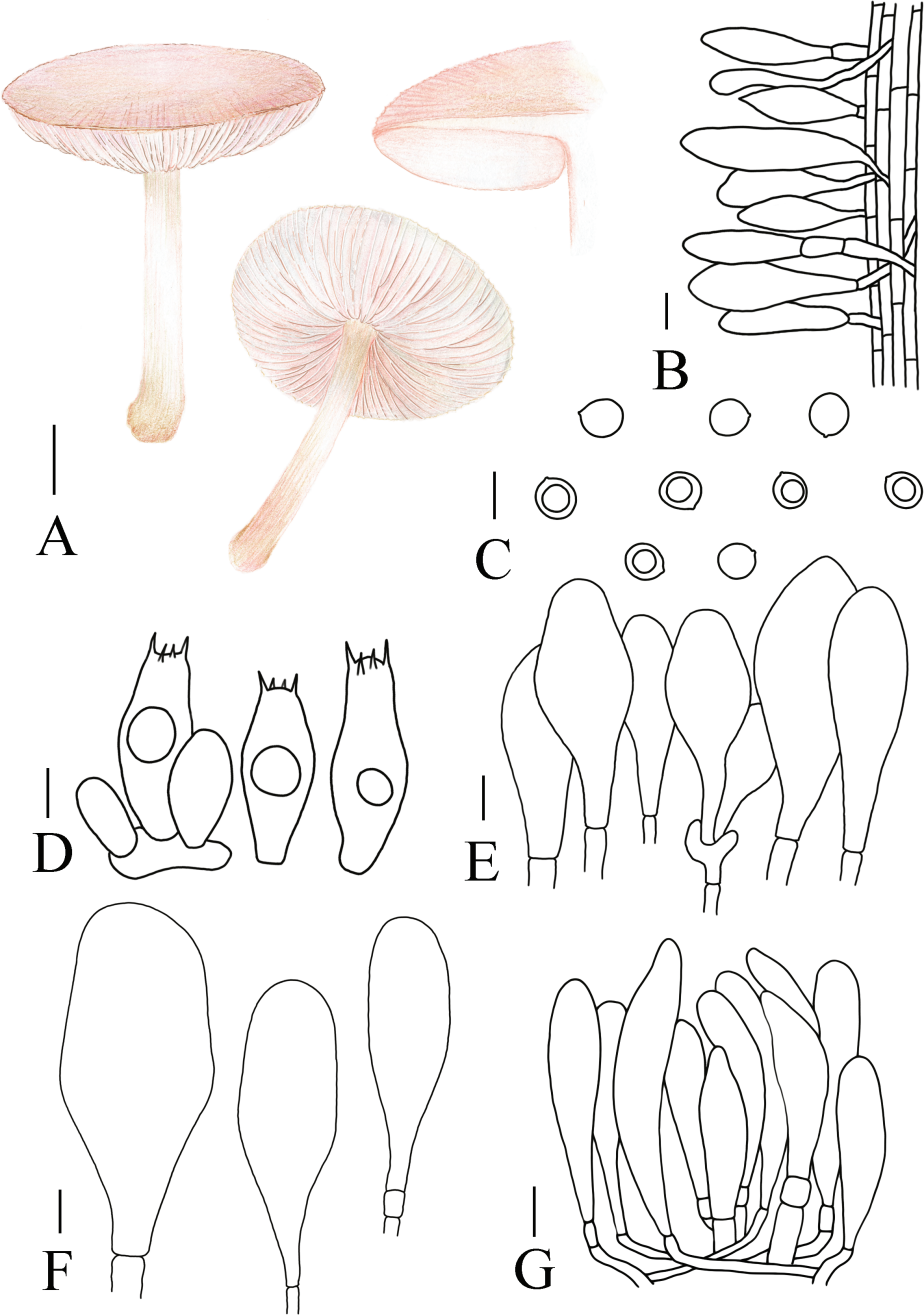
A. Macroscopic characteristics of *Pluteusultraputripiceae*. B. Caulocystidia. C. Basidiospores. D. Basidia. E. Cheilocystidia. F. Pleurocystidia. G. Pileipellis elements. Scale bars: 1 cm (A); 10 µm (B–G).

##### ﻿ Pluteus hinnuleus

Taxon classificationFungiAgaricalesPluteaceae

Z.X. QI, B. Zhang & Y. Li sp. nov.

FD3B3022-FFBF-5139-99B7-E02A4B4293CA

MycoBank No: 857277

Figs 7D, E , 13

###### Etymology.

The species epithet “hinnuleus” (Latin): refers to the clay-buff to earthy yellow pileus color.

###### Diagnosis.

*Pluteushinnuleus* differs from *P.albivillus* by its clay-buff to earthy yellow pileus color, small basidiospores, pleurocystidia with 1–7 μm finger-like projections on the apex. It grows preferentially in birch forests (*Betula*) on decaying wood branches and is distributed in Northeast China. The ITS genetic distance is 0.125 (SE = 0.017).

###### Holotype.

CHINA • Heilongjiang Province, Shuanghe National Nature Reserve, 12 July 2019, D.Z. Guo, FJAU66614 (ITS: PQ810768, LSU: PQ810746, *tef1*: PQ811064) (Collection no.: Guo 377).

###### Description.

Basidiomata medium-sized. Pileus 34 mm diam; convex to slightly hemispherical; clay-buff to earthy yellow (2.5Y 8/6), dry, the surface with recurved squamules, the central scales color dark brown (10YR 4/6), the margin slightly inflexed. Lamellae free, 3–5 mm wide, moderately crowded, thick, pink (2.5YR 7/4), unequal, slightly ventricose, edges even or flocculose; lamellar edge concolorous to the sides. Stipe 43 × 4–7 mm, fibrous, solid, earthy yellow (2.5Y 8/6), with brown glandular dots on the surface (7.5YR 4/2), slightly inflated at the base. Spore print pink.

Basidiospores [50/1/1] 6.5–7.5(–8.0) × (–5.0)5.5–6.5 (–7.0) µm, avL × avW = 6.8–7.2 × 5.7–6.0 µm, Q = 1.16–1.33 µm, avQ = 1.20–1.25 µm, mostly subglobose, broadly ellipsoid to ellipsoid, smooth, slightly pinkish, thin-walled. Basidia 25–34 × 9–11 μm, clavate, thin-walled, 4-sterigmate, hyaline. Pleurocystidia 60–93 × 20–35 μm, abundant, scattered, two forms, one broadly fusiform with bluntly rounded apices, the other narrowly fusiform with a longer neck and unequal apical rostrate, long projection 3–7 μm long, thin-walled, smooth, hyaline. Lamellar edge sterile. Cheilocystidia 43–78 × 15–24 μm, clustered, numerous, broadly clavate to clavate, apically obtuse, partly with brown intracellular pigment, thin-walled, smooth. Pileipellis a hymeniderm, with terminal elements 81–155 × 19–33 μm, broadly clavate or broadly cylindrical, thin-walled, with brown intracellular pigment. Stipitipellis a cutis, hyphae 4–6 μm diam, cylindrical, hyaline, thin-walled. Caulocystidia 31–51 × 15–25 μm, clustered, long clavate, apically obtusely rounded, with brownish intracellular pigment, smooth, thick-walled. Clamp connections absent in all tissues.

###### Habitat.

Solitary on decaying wood in birch forests (*Betulaplatyphylla* Sukaczev).

###### World distribution.

China.

###### China distribution.

Heilongjiang Province.

###### Notes.

*Pluteushinnuleus* is primarily characterized by its dry pileus surface bearing recurved squamules, clay-buff to earthy yellow coloration, and ecological preference for rotting wood in *B.platyphylla* forests.

Morphologically, *P.hinnuleus* resembles *P.albivillus*, with both species exhibiting hemispherical pileus and firm, fibrous stipes. However, these taxa can be differentiated by basidiospore dimensions, pleurocystidia morphology, and substrate ecology. *P.hinnuleus* produces smaller basidiospores (avL × avW = 6.8–7.2 × 5.7–6.0 µm) with pleurocystidia featuring 1–7 μm finger-like apical projections, and occurs on decaying wood in *Betulaplatyphylla* forests. In contrast, *P.albivillus* forms larger basidiospores (avL × avW = 7.3–7.6 × 5.8–6.0 µm) with pleurocystidia lacking apical projections, and is associated with decaying wood in *Larixsibirica* forests.

Phylogenetically, *P.hinnuleus* is closely related to *P.dianae* and P.aff.dianae, though morphologically distinct from both. *P.hinnuleus* differs from *P.dianae* by its slightly smaller basidiospores (avL × avW = 6.8–7.2 × 5.7–6.0 µm versus avL × avW = 7.2 × 6.3 µm in *P.dianae*), with an ITS genetic distance of 0.041 (SE = 0.009) supporting this separation. Similarly, *P.hinnuleus* is distinguished from P.aff.dianae by its larger basidiospores (avL × avW = 6.8–7.2 × 5.7–6.0 µm versus avL × avW = 6.6 × 5.3 µm in P.aff.dianae) and geographic distribution in East Asia (China) rather than Europe (Spain). This distinction is further supported by an ITS genetic distance of 0.047 (SE = 0.010).

**Figure 13.**
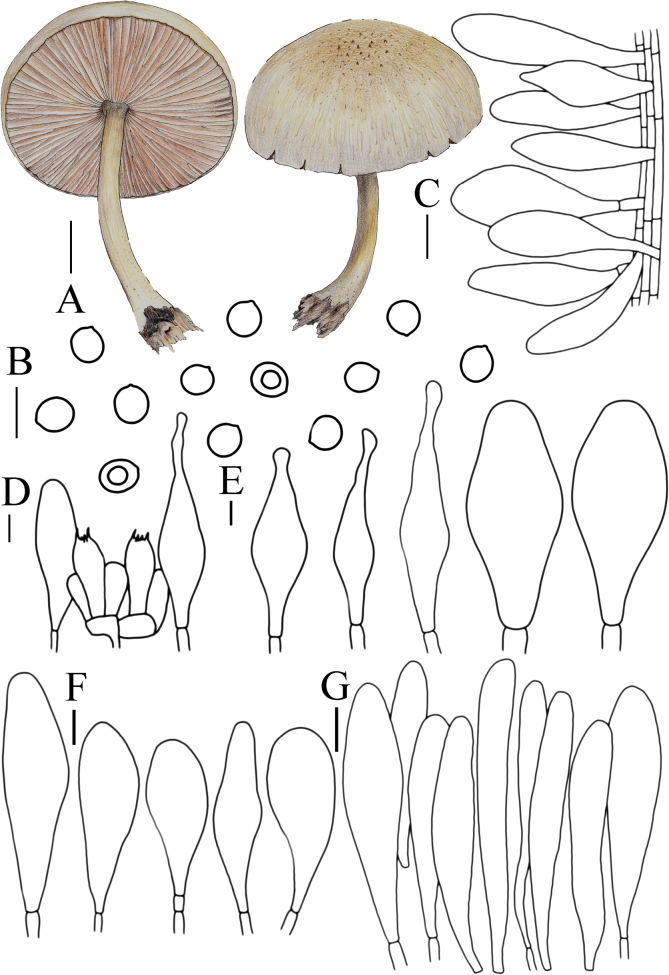
A. Macroscopic characteristics of *Pluteushinnuleus*. B. Basidiospores. C. Caulocystidia. D. Basidia and Pleurocystidia. E. Pleurocystidia. F. Cheilocystidia. G. Pileipellis elements. Scale bars: 1 cm (A); 10 µm (B–F); 20 µm (G).

##### ﻿ Pluteus baishanzuensis

Taxon classificationFungiAgaricalesPluteaceae

Z.X. QI, B. Zhang & Y. Li sp. nov.

7904921A-C946-57A2-8A85-58B6A405800F

MycoBank No: 857278

Figs 6C–G , 14

###### Etymology.

baishanzu, for the geographic origin of the type collection.

###### Diagnosis.

*Pluteusbaishanzuensis* differs from *P.lauracearum* by its brown glandular dots on the surface of the pileus, exceeding lamellae on the margin of the pileus, small basidiospores, and thin-walled pleurocystidia. The ITS genetic distance is 0.073 (SE = 0.014).

###### Holotype.

CHINA • Zhejiang Province, Baishanzu National Forest Park, 27°56'26.52"N, 119°18'79.45"E, alt. 1525 m. 2 August 2024, R.P. Liu, FJAU66622 (Collection no.: Liu 191) (ITS: PQ810761, LSU: PQ810738, *tef1*: PQ811048).

###### Description.

Basidiomata medium-sized. Pileus 23–32 mm diam; convex or plano-convex; surface white (2.5Y 9/2), central part covered with brown (5YR 5/2) to light brown (2.5Y 5/2) glandular dots or finely pruinose, forming dark brown (2.5YR 5/2) vein-like striate, extend to the margin; smooth, hygrophanous; margin exceeding lamellae. Context white (2.5P 9/2). Lamellae free, 3–7 mm wide, moderately crowded, initially white (10P 9/2) to later flesh-pink (5R 9/2), unequal, thicker, slightly ventricose, edges flocculose, and white or slightly pink. Stipe 36–46 × 1–3 mm, fibrous, hollow, clavate, base expanded into a bulbous, the upper part of the stipe hyaline (2.5P 9/2) to white (10P 9/2), transitioning downward to brown (5YR 5/2) to dark brown (2.5YR 5/2), the surface covered with white finely pruinose (10P 9/2). Spore print pink.

Basidiospores [120/5/2] 6.0–6.5(–7.0) × 5.0–6.0(–6.5) µm, avL × avW = 6.2–6.5 × 5.3–5.6 µm, Q = 1.08–1.20 µm, avQ = 1.13–1.18 µm, subglobose to broadly ellipsoid, smooth, slightly pinkish, thin-walled. Basidia 24–31 × 8–12 μm, clavate to broadly clavate, thin-walled, 4-sterigmate. Pleurocystidia 47–95 × 16–31 μm, rare, scattered, fusiform or broadly subfusiform, bluntly rounded apically, thin-walled, smooth, hyaline. Lamellar edge sterile. Cheilocystidia 35–66 × 12–21 μm, abundant, clustered, mostly clavate to narrowly clavate, a few subfusiform, bluntly rounded apically, thin-walled, smooth, hyaline. Pileipellis a hymeniderm, with terminal elements 66–133 × 19–25 μm, long clavate, narrowly clavate, subcylindric or subfusiform, thin-walled, with brown intracellular pigment. Stipitipellis a cutis, hyphae 4–7 μm diam, cylindrical, hyaline, thin-walled. Caulocystidia 31–53 × 15–28 μm, clustered, narrowly clavate to clavate or broadly clavate, apically obtusely rounded, with brownish intracellular pigment, smooth, thick-walled. Clamp connections absent in all tissues.

###### Habitat.

Solitary to scattered on decaying wood of broad-leaved trees (*Quercusmyrsinifolia* Blume).

###### World distribution.

China.

###### China distribution.

Zhejiang Province.

###### Additional specimens examined.

CHINA • Zhejiang Province, Baishanzu National Forest Park, 27°76'5.02"N, 119°18'79.54"E, alt. 1535 m. 1 August 2024, R.P. Liu, FJAU66621 (Collection no.: Liu 178) (ITS: PQ810762, LSU: PQ810739, *tef1*: PQ811049).

###### Notes.

*Pluteusbaishanzuensis* is distinguished by its thin pileus with brownish glandular dots on the surface, moderately crowded lamellae, and distribution restricted to East Asia (China).

Morphologically, *P.baishanzuensis* shares similarities with *P.lauracearum* but can be differentiated by several key features: *P.baishanzuensis* possesses an unstriate pileus margin and smaller basidiospores (avL × avW = 6.2–6.5 × 5.3–5.6 µm), while *P.lauracearum* exhibits striate pileus margin and larger basidiospores (avL × avW = 7.3 × 6.0 µm). These taxa also differ in their geographic distribution, with *P.baishanzuensis* occurring in East Asia (China) and *P.lauracearum* in Central Asia and Europe (Turkey and Portugal) (Kaygusuz et al. 2021). This distinction is further supported by an ITS genetic distance of 0.073 (SE = 0.014) between these species.

Phylogenetically, *P.baishanzuensis* is closely related to P.cf.velutinus, although these taxa can be readily distinguished by both macroscopic and microscopic characters. Macroscopically, *P.baishanzuensis* exhibits dark brown vein-like striate on the pileus surface, whereas P.cf.velutinus has an entirely granulose-pruinose or slightly velvety pileus. Microscopically, *P.baishanzuensis* produces predominantly fusiform pleurocystidia with bluntly rounded apices and cheilocystidia lacking elongated necks. In contrast, P.cf.velutinus is characterized by polymorphic pleurocystidia with partly finger-like apices and cheilocystidia that are partially fusiform with longer, irregular necks (Malysheva et al. 2020; Malysheva et al. 2023). The ITS genetic distance between these taxa is 0.018 (SE = 0.006).

**Figure 14.**
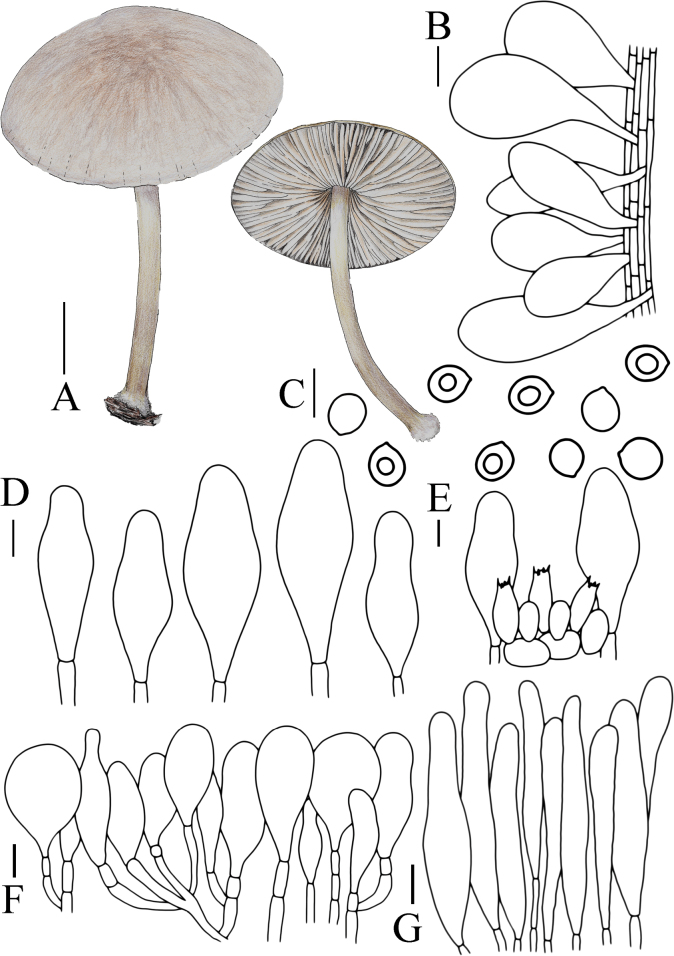
A. Macroscopic characteristics of *Pluteusbaishanzuensis*. B. Caulocystidia. C. Basidiospores. D. Pleurocystidia. E. Basidia and Pleurocystidia. F. Cheilocystidia. G. Pileipellis elements. Scale bars: 1 cm (A); 10 µm (B–F); 20 µm (G).

##### ﻿ Pluteus albivillus

Taxon classificationFungiAgaricalesPluteaceae

Z.X. QI, B. Zhang & Y. Li sp. nov.

AE556490-7A7C-52A8-B1C1-F60DE0039565

MycoBank No: 857279

Figs 6A, B , 15

###### Etymology.

Derived from Latin ‘albus’ (white) and ‘villus’ (downy), referring to the white downy hairs on the pileus surface.

###### Diagnosis.

*Pluteusalbivillus* differs from *P.velutinus* by its pileus surface with white villi, flocculose lamellae edges, fibrous stipe, and two forms of pleurocystidia (fusiform and narrowly clavate to clavate). The ITS genetic distance is 0.058 (SE = 0.010), *tef1* genetic distance is 0.106 (SE = 0.031).

###### Holotype.

CHINA • Xinjiang Uygur Autonomous Region, Altay Kanas National Forest Park. 48°41'26.33"N, 87°02'33.09"E, alt. 1287 m. Scattered on decaying wood of Xinjiang larch (*L.sibirica*). 21 August 2022, Z.X. Qi, FJAU66613 (Collection no.: Qi 1117) (ITS: PQ810759, LSU: PQ810736, *tef1*: PQ811046).

###### Description.

Basidiomata medium-sized. Pileus 24–29 mm diam; hemispherical when young, brown (10R 5/2); expanding to convex, moderate reddish brown (10R 5/2) to weak reddish brown (2.5R 5/2); surface covered with fine villous covering when dry, hygrophanous when damp, smooth, margin inflexed. Lamellae free, 3–5 mm wide, crowded, initially white (10P 9/2) to later pink (5R 9/2), unequal, thicker, with flocculose edges; lamellar edge concolorous to the sides. Stipe 30–35 × 3–5 mm, clavate, slightly expanded at the base, fibrous, surface with longitudinal striate, hyaline to white (10P 9/2) in the upper part, transitioning downward to brown (10R 5/2) to dark brown (2.5R 5/2). Spore print unknown.

Basidiospores [90/3/1] (–6.5)7.0–8.0(–8.5) × 5.5–6.5(–7.0) µm, avL × avW = 7.3–7.6 × 5.8–6.0 µm, Q = 1.07–1.34 µm, avQ = 1.20–1.25 µm, subglobose, broadly ellipsoid to ellipsoid, smooth, slightly pinkish, thin-walled. Basidia 26–32 × 7–10 μm, clavate, thin-walled, 4-sterigmate, hyaline. Pleurocystidia 49–86 × 20–36 μm, few, scattered, two forms, one narrowly clavate to clavate, bluntly rounded apically, the other long fusiform, pointed apically, thin-walled, smooth, hyaline. Lamellar edge sterile. Cheilocystidia 35–66 × 12–21 μm, abundant, clustered, clavate, pyriform to narrowly clavate, smooth, thin-walled, hyaline. Pileipellis a hymeniderm formed of variable and often mixed elements, from short and rounded to clavate, subfusiform or cylindrical terminal elements, 57–130 × 15–36 μm, with pale brown intracellular pigment, thin-walled. Stipitipellis a cutis, hyphae 3–6 μm diam, cylindrical, hyaline, thin-walled. Caulocystidia 33–67 × 11–20 μm, clustered, narrowly clavate to clavate, apically obtusely rounded, smooth, thin-walled, hyaline. Clamp connections absent in all tissues.

###### Habitat.

Scattered on decaying wood of Xinjiang larch (*L.sibirica*).

###### World distribution.

China.

###### China distribution.

Xinjiang Uygur Autonomous Region.

###### Notes.

*Pluteusalbivillus* is primarily characterized by fine white villi on the pileus surface (which appear smooth when wet), flocculose lamellae edges, dimorphic pleurocystidia (one form narrowly clavate to clavate, the other long-fusiform), and ecological association with decaying *Larixsibirica* wood.

Morphologically, *P.albivillus* shares similarities with *P.velutinus*, both producing basidiomata and basidiospores of comparable dimensions (Malysheva et al. 2016; Ferisin and Dovana 2018). However, these taxa can be distinguished by both macroscopic and microscopic features. Macroscopically, *P.albivillus* exhibits flocculose lamellae edges and a harder, more fibrous stipe, whereas *P.velutinus* has flush lamellae edges and a brittle, bony stipe. Microscopically, *P.albivillus* produces dimorphic pleurocystidia (narrowly clavate to clavate and fusiform) lacking apical projections, and narrowly clavate cheilocystidia. In contrast, *P.velutinus* forms uniformly fusiform pleurocystidia, some bearing 1–2 unequal apical projections, and lageniform to narrowly utriform cheilocystidia with elongated necks (Malysheva et al. 2016; Ferisin and Dovana 2018).

*P.albivillus* might also be confused with *P.punctipes* due to the brownish pileus tones and minute fibrous scales on the surface of both species. These taxa can be distinguished primarily by pleurocystidia dimensions, with *P.albivillus* producing shorter elements (≤86 µm) compared to the longer pleurocystidia (≤100 µm) of *P.punctipes* (Orton 1960).

Phylogenetically, *P.albivillus* forms a distinct, well-supported branch (MLB = 97, BPP = 0.99, Fig. 1), paraphyletic with *P.baishanzuensis*. *P.albivillus* is distinguished from *P.baishanzuensis* by its pileus with fine white villi, larger basidiospores (avL × avW = 7.3–7.6 × 5.8–6.0 µm), and preference for gymnosperm substrates in northwestern China (Xinjiang). In contrast, *P.baishanzuensis* exhibits a pileus with brown glandular dots, smaller basidiospores (avL × avW = 6.2–6.5 × 5.3–5.6 µm), and a preference for angiosperm substrates in southeastern China (Zhejiang). These distinctions are further supported by genetic distances of 0.037 (SE = 0.008) for ITS and 0.105 (SE = 0.017) for *tef1*.

**Figure 15.**
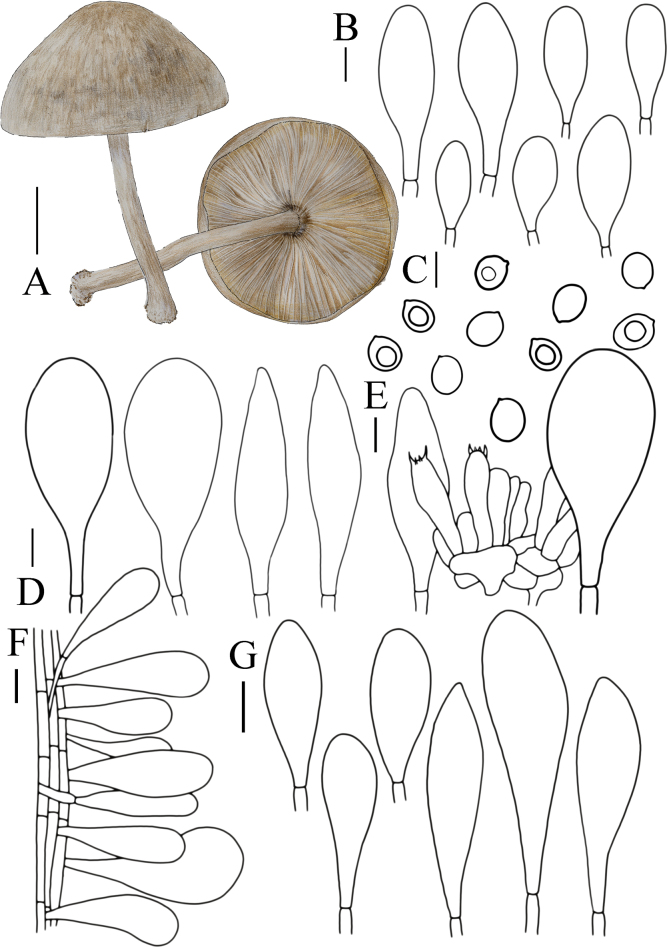
A. Macroscopic characteristics of *Pluteusalbivillus*. B. Cheilocystidia. C. Basidiospores. D. Pleurocystidia. E. Basidia and Pleurocystidia. F. Caulocystidia. G. Pileipellis elements. Scale bars: 1 cm (A); 5 µm (C); 10 µm (B, D–F); 20 µm (G).

##### ﻿ Pluteus velutinus

Taxon classificationFungiAgaricalesPluteaceae

C.K. Pradeep, Justo & K.B. Vrinda, Mycol. Progr. 11(4): 871 (2012)

9E5C6E2B-B94B-5853-83EC-D6706F0E8163

Figs 6H–J , 16

###### Description.

Basidiomata medium-sized. Pileus 11–37 mm diam; hemispherical to plano-convex, with obtuse umbo; surface fleshy brown (5YR 6/4), slightly vein-like projections, margin indistinctly striped, inflexed. Context yellowish (7.5P 9/2). Lamellae free, 3–6 mm wide, moderately crowded, flesh pink (5R 9/2), unequal, with transvenose, thicker, slightly ventricose, edges even. Stipe 15–43 × 2–5 mm, rod-shaped, base expanded and slightly curved, fibrous, hollow, white (10P 9/2) to light fleshy brown (7.5YR 6/4). Spore print pink.

Basidiospores [120/4/2] 5.5–6.5 × 5.0–5.5(–6.0) µm, avL × avW = 6.0–6.2 × 5.3–5.5 µm, Q = 1.09–1.30 µm, avQ = 1.15–1.20 µm, subglobose to broadly ellipsoid, smooth, slightly pinkish, thin-walled. Basidia 25–31 × 9–10 μm, clavate, thin-walled, 4-sterigmate, hyaline. Pleurocystidia 55–78 × 14–25 μm, few, scattered, long fusiform to subfusiform, with a long neck and a conspicuous transverse septum, one part apically mucronate, the other bluntly rounded, thin-walled, smooth, a few containing brown intracellular pigment. Lamellar edge sterile. Cheilocystidia 33–61 × 12–18 μm, abundant, clustered, two forms, one long fusiform with a long neck (7–13 μm), the other apically expanded fusiform, thin-walled, smooth, hyaline. Pileipellis a trichoderm or trichohymeniderm, with terminal elements 71–145 × 21–26 μm, long clavate, subcylindric or subfusiform, thin-walled, with brown intracellular pigment. Stipitipellis a cutis, hyphae 4–7 μm diam, cylindrical, hyaline, thin-walled. Caulocystidia 29–49 × 12–21 μm, clustered, composed of irregular clavate, subcylindrical or sublageniform, partly containing brownish intracellular pigment, thin-walled, smooth. Clamp connections absent in all tissues.

###### Habitat.

Solitary to scattered on decaying wood in mixed forests (*Pinus* sp. and *Quercus* sp.).

###### World distribution.

India, Brazil, Mongolia, Russia, Italy (Malysheva et al. 2016; Ferisin and Dovana 2018), China.

###### China distribution.

Hubei Province (Wang JF 2019), Jilin Province.

###### Additional specimens examined.

CHINA • Jilin Province, Hongye Valley Forest Park. 43°36'8.85"N, 126°58'22.19"E, 2 August 2021, G. Rao, FJAU66619 (Collection no.: Rao 936) (ITS: PQ810764, LSU: PQ810742). CHINA • Jilin Province, Cold Onion Ridge Forest Park; 43°02'2.06"N, 127°58'44.69"E, Solitary to scattered on decaying wood in mixed forests (*Pinus* sp. and *Quercus* sp.); 1 August 2021, G. Rao, FJAU66620 (Collection no.: Rao 1261) (ITS: PQ810765, LSU: PQ810743).

###### Notes.

*Pluteusvelutinus* was found in India and, in subsequent years, in Brazil, Mongolia, Russia and Italy (Malysheva et al. 2016; Ferisin and Dovana 2018). The original description reported basidiospores 5.5–9.5 × 5–7.0 µm, Q = 1.17–1.50 µm. Our measurements are within the range of the original description and are closer to the data from the Russia (Siberian) collection: 5.7–6.5(–7.5) × 5.4–6.0(–6.3) µm, Q = 1.00–1.16 (–1.20) µm (Malysheva et al. 2016).

In the phylogenetic tree, the sequences of the two specimens taken from China clustered in the same branch with the type specimen with high support (MLB = 99, BPP = 1, Fig. 2). The results of the morphology and phylogenetic tree identified it as *P.velutinus*, and here we treat this species as a common species.

**Figure 16.**
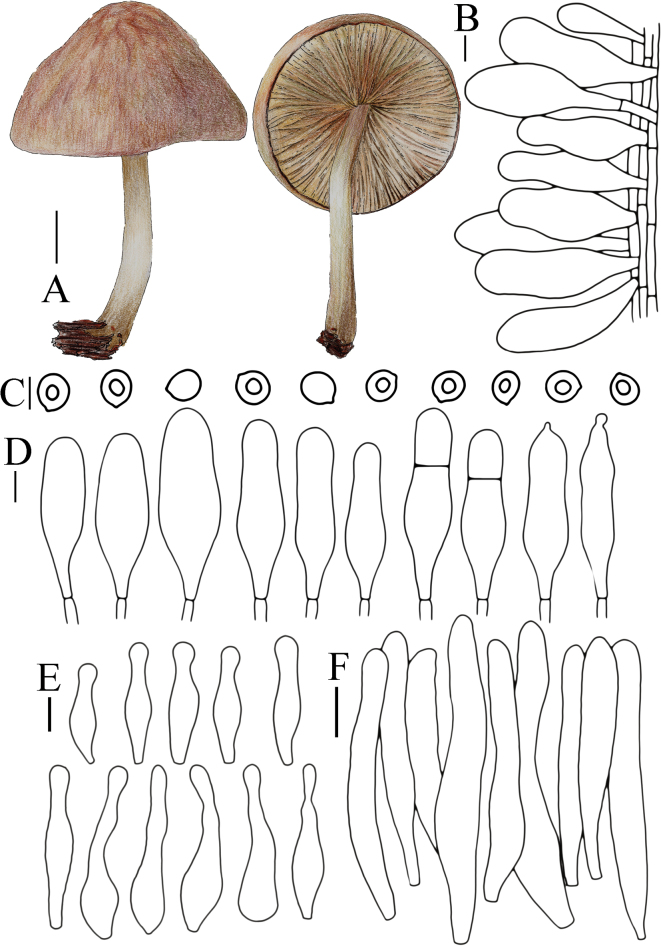
A. Macroscopic characteristics of *Pluteusvelutinus*. B. Caulocystidia. C. Basidiospores. D. Pleurocystidia. E. Cheilocystidia. F. Pileipellis elements. Scale bars: 1 cm (A); 5 µm (C); 10 µm (B, D, E); 20 µm (F).

##### ﻿ Pluteus longistriatus

Taxon classificationFungiAgaricalesPluteaceae

(Peck) Peck, Ann. Rep. N.Y. St. Mus. nat. Hist. 38: 137 (1885)

A75D4DD2-4403-538C-86B0-E16639CA1164

Figs 6K, L , 17

###### Description.

Basidiomata medium-sized. Pileus 31–35 mm diam; hemispherical to plano-convex, smooth, surface brownish-brown (5.0YR 6/10), darker in the center (5.0YR 4/10), with brown longitudinal striate extending from the middle to the margin. Lamellae dirty white (5.0YR 9/4), free, relatively crowded, thick, unequal, slightly ventricose, 2–4 mm wide, with even edges. Stipe 26–31 × 3–5 mm, cylindrical, slightly thicker at the base, fibrous, with white longitudinal fibrils on the surface (5.0YR 9/2). Odorless. Spore prints pink.

Basidiospores [60, 3, 1] (–6.0)6.5–7.0(–7.5) × 5.0–6.0(–6.5) μm, avL × avW = 6.7–7.0 × 5.8–6.1 µm, Q = 1.03–1.25 μm, avQ = 1.06–1.15 μm, globose to subglobose, or broadly ellipsoid, slightly pinkish, smooth, thin-walled or slightly thick. Basidia 25–31 × 9–11 μm, broadly rod-shaped or clavate, thin-walled, 4-sterigmate, hyaline. Pleurocystidia 55–88 × 16–27 μm, numerous, scattered, fusiform or narrowly utriform, apically obtusely rounded, thin-walled, smooth, hyaline. Lamellar edge sterile. Cheilocystidia 41–76 × 18–25 μm, abundant, clustered, narrowly clavate to clavate or long clavate, thin-walled, hyaline. Pileipellis a hymeniderm, with terminal elements 60–114 × 19–28 μm, broadly clavate or fusiform, thin-walled, with brown intracellular pigment. Stipitipellis a cutis, hyphae 4–11 µm diam, cylindrical, hyaline, thin-walled. Caulocystidia 29–72 × 12–26 μm, numerous, occurring in clusters, clavate to oblong-clavate, or fusiform, apically obtuse or mucronate, hyaline, thin-walled. Clamp connections absent in all tissues.

###### Habitat.

Scattered on rotting wood in poplar forests (*Populustalassica*).

###### World distribution.

USA, Paraguay, Brazil, Argentina (Justo et al. 2011a; Ferisin and Dovana 2016; Campi et al. 2019), China (Xu 2016).

###### China distribution.

Yunnan Province, Jilin Province (Xu 2016).

###### Additional specimens examined.

CHINA • Jilin Province, Changchun City, Jilin Agricultural University. Scattered on rotting wood in poplar forests (*Populustalassica*); 42°17'66.65"N, 82°47'94.24"E, alt. 253 m, 10 July 2021, Z.X. Qi, Z.H. Zhang, FJAU66596 (Collection no.: Qi 302) (ITS: PP516605, LSU: PP516655).

###### Notes.

*Pluteuslongistriatus* is mainly characterized by a brown, smooth pileus with a distinctly brown and long radial stripe on the surface, extending from the center to the margin (Ferisin and Dovana 2016; Campi et al. 2019).

*P.longistriatus* is similar to *P.heteromarginatus* in macromorphology, differing only in the shape of the pleuro, cheilo, and caulocystidia. *P.longistriatus* has pleurocystidia fusiform or narrowly utriform, apically obtusely rounded, cheilocystidia narrowly clavate to clavate or long clavate, and clavate to oblong-clavate, or fusiform caulocystidia. In contrast, *P.heteromarginatus* contains pleurocystidia with elongated apexes or an apical flexuous excrescence (5–10 µm long), cheilocystidia (narrowly) clavate, narrowly utriform, or more rarely obovoid, and fusiform or lageniform caulocystidia (Justo et al. 2011a). *P.longistriatus* is confused with *P.atriavellaneus* Murrill, which is characterized by the presence of brown pileus, with striped margins. On the other hand, the basidiospores of *P.atriavellaneus* are subglobose, slightly larger, and 7.0–8.0 μm in diameter (Campi et al. 2019).

According to the phylogenetic analyses, *P.longistriatus* specimen FJAU66596 from China clustered with specimens from Russia and South Korea with high support (MLB = 100, BPP = 1, Fig. 2).

**Figure 17.**
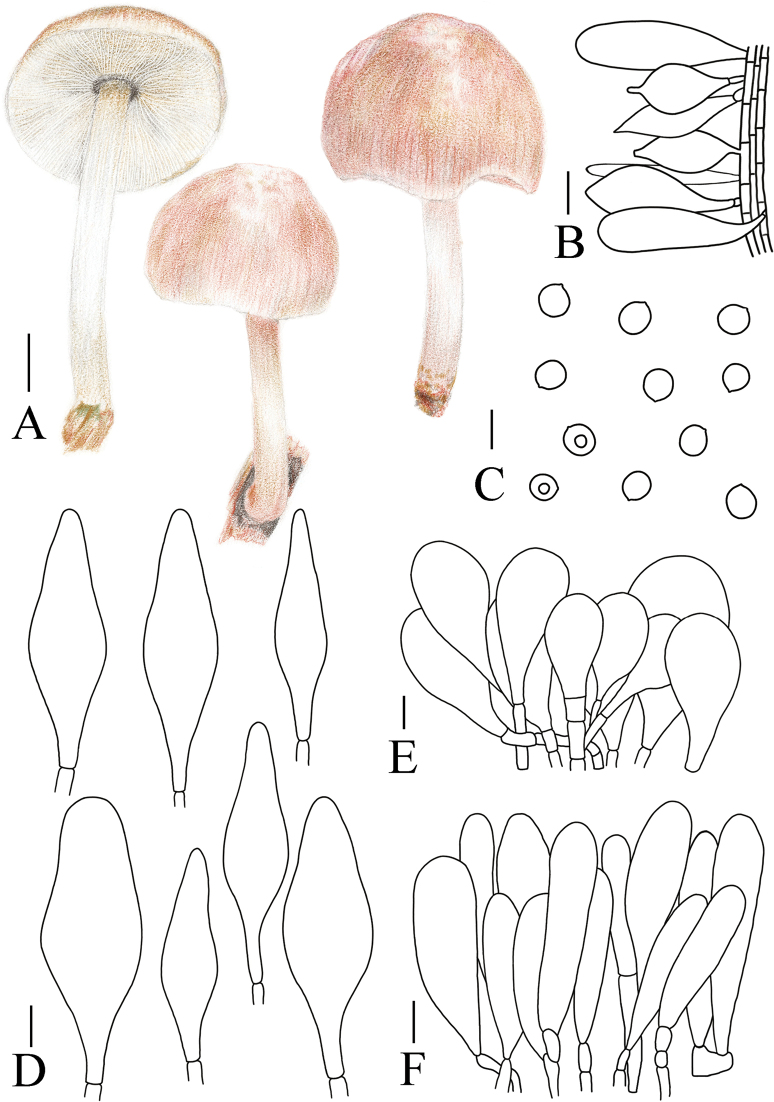
A. Macroscopic characteristics of *Pluteuslongistriatus*. B. Caulocystidia. C. Basidiospores. D. Pleurocystidia. E. Cheilocystidia. F. Pileipellis elements. Scale bars: 1 cm (A); 10 µm (C–E); 20 µm (B, F).

#### ﻿/*umbrosus*/*granularis* clade

(Figs 18–22)

**Figure 18.**
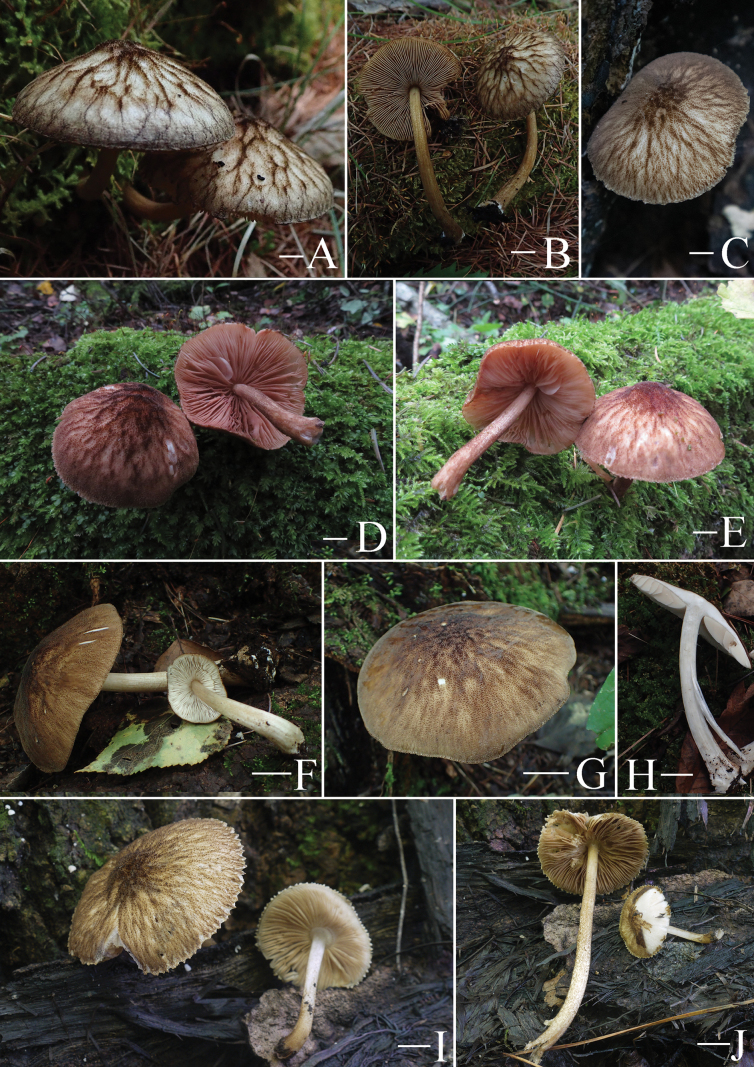
Basidiomata features. A–C. *Pluteuscostatus* (A, B. FJAU66589, C. FJAU66618). D, E. *P.umbrosus* (FJAU66590). F–H. *P.umbrosoides* (FJAU66615). I, J. *P.granularis* (FJAU66612). A–C. Photos by Di-Zhe Guo. D, E. Photos by Jia-Jun Hu. C, F–H. Photos by Gu Rao. I, J. Photos by Zheng-Xiang Qi. Scale bars: 1 cm.

**Figure 19.**
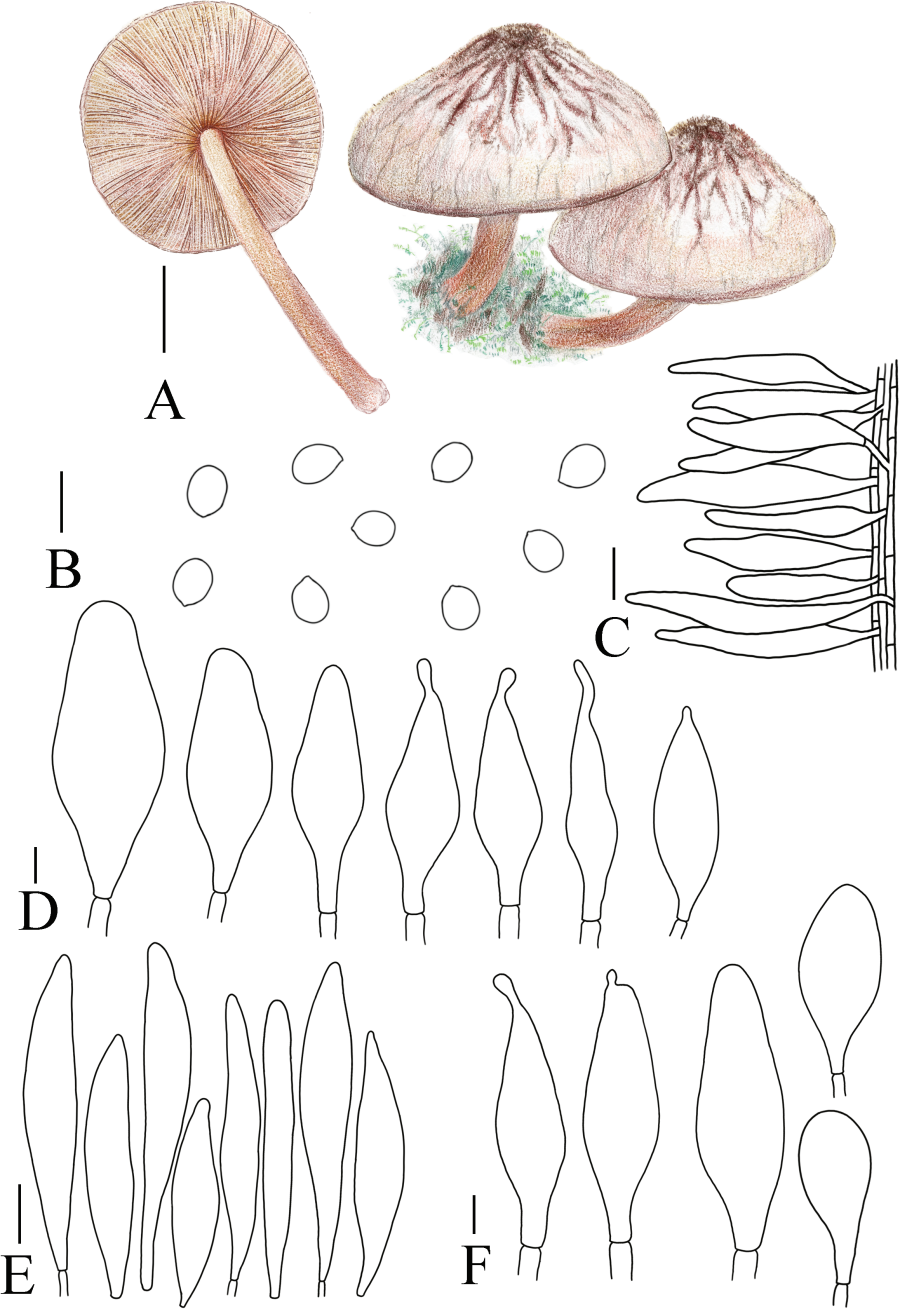
A. Macroscopic characteristics of *Pluteuscostatus*. B. Basidiospores. C. Caulocystidia. D. Pleurocystidia. E. Pileipellis elements. F. Cheilocystidia. Scale bars: 1 cm (A); 10 µm (B, D, F); 20 µm (C, E).

**Figure 20.**
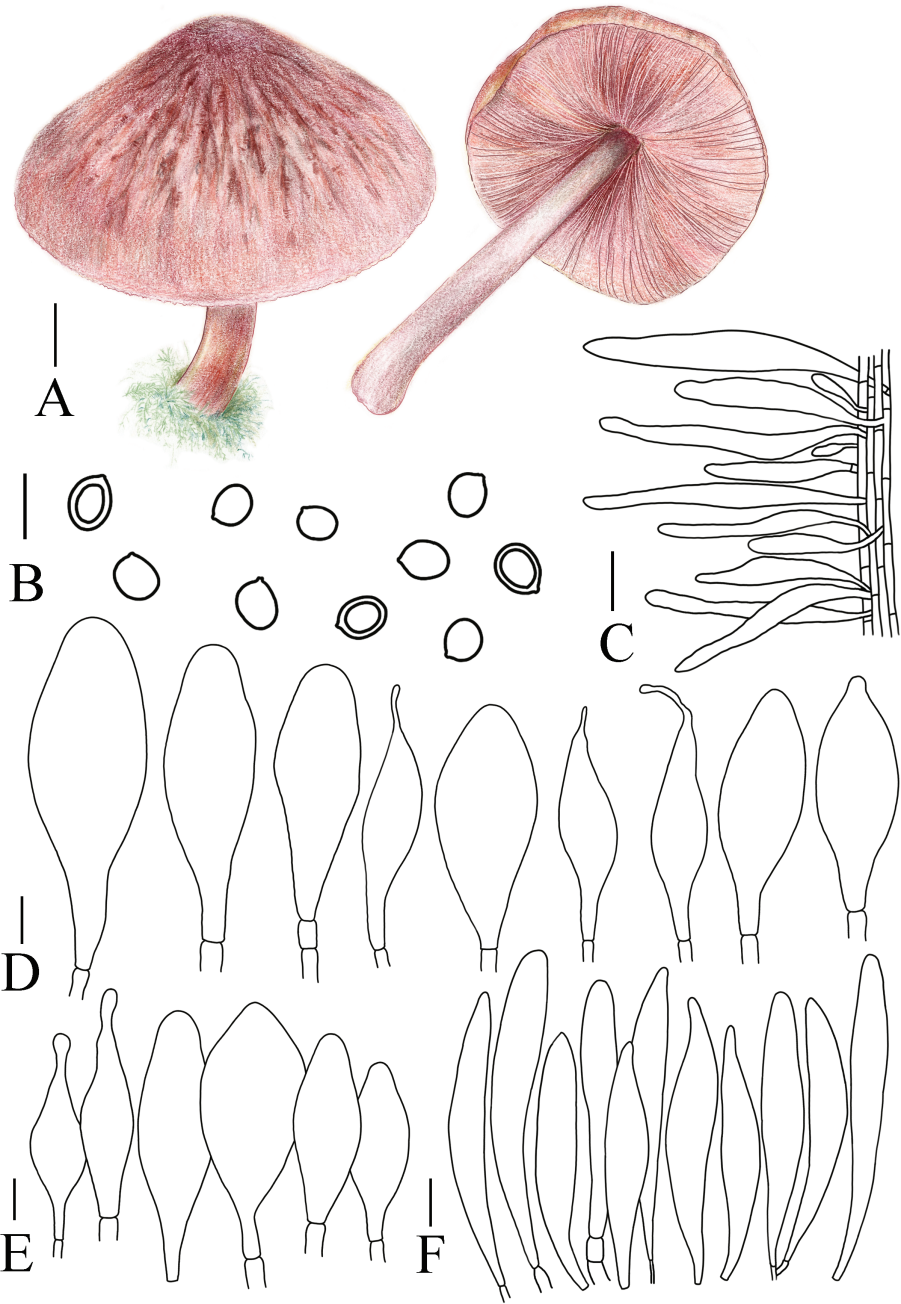
A. Macroscopic characteristics of *Pluteusumbrosus*. B. Basidiospores. C. Caulocystidia. D. Pleurocystidia. E. Cheilocystidia. F. Pileipellis elements. Scale bars: 1 cm (A); 10 µm (C, D, E); 20 µm (B, F).

**Figure 21.**
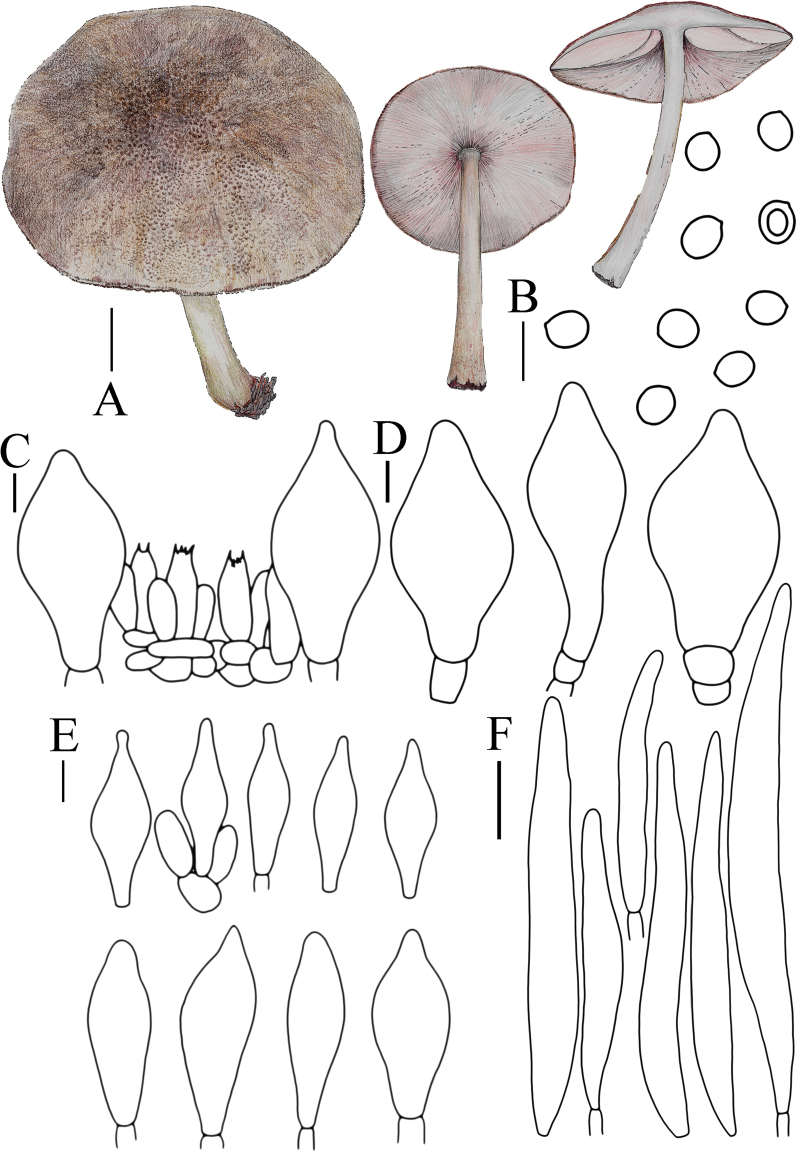
A. Macroscopic characteristics of *Pluteusumbrosoides*. B. Basidiospores. C. Basidia and Pleurocystidia. D. Pleurocystidia. E. Cheilocystidia. F. Pileipellis elements. Scale bars: 1 cm (A); 10 µm (B–E); 20 µm (F).

**Figure 22.**
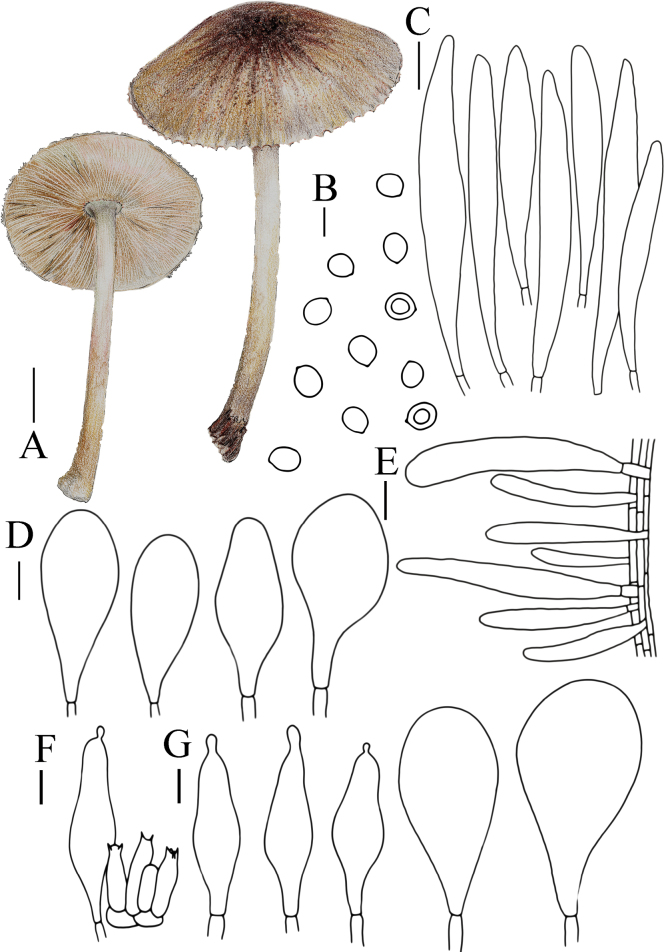
A. Macroscopic characteristics of *Pluteusgranularis*. B. Basidiospores. C. Pileipellis elements. D. Cheilocystidia. E. Caulocystidia. F. Basidia and Pleurocystidia. G. Pleurocystidia. Scale bars: 1 cm (A); 5 µm (B); 10 µm (D–G); 20 µm (C).

##### ﻿ Pluteus costatus

Taxon classificationFungiAgaricalesPluteaceae

Z.X. QI, B. Zhang & Y. Li sp. nov.

4D372574-7602-5EE0-861B-126C6A929FE6

MycoBank No: 853187

Figs 18A–C , 19

###### Etymology.

The species epithet “costatus” (Lat.) refers to the ribbed (costatus) pileus, characterized by prominent veins, with a distinctly pronounced middle vein.

###### Diagnosis.

Morphologically similar to *P.ornatus*, differing in its unstriped pileus margins, smaller basidiospores avL × avW = 6.2–6.5 × 5.2–5.5 µm, and their ITS genetic distance is 0.029 (SE = 0.007). It grows in coniferous forests with decaying wood branches and is distributed in East Asia (China). Phylogenetically close to *P.umbrosus* and *P.granularis*, differing in the lamellae with brown edges.

###### Holotype.

CHINA • Heilongjiang Province, Shuanghe National Nature Reserve, 13 July 2019, D.Z. Guo, FJAU66589 (ITS: PP516603, LSU: PP919365, *tef1*: PP551591) (Collection no.: Guo 380).

###### Description.

Basidiomata medium-sized. Pileus 32–35 mm diam; convex or plano-convex, often with a low, broad umbo; overall silvery gray (5.0YR 6/2), with brown granulose-squamulose or micaceous on the surface, forming a black-brown vein-like stripe (5.0YR 4/10), very dense in the center; veins extended to the margin, gradually less; margin striaght. Lamellae white to light pink (2.5YR 9/2–2.5R 9/6), free, crowded, thick, unequal, 3–5 mm wide, with white to light brown and flocculose edges. Stipe 61–66 × 5–8 mm, cylindrical, hollow, fibrous, brown (5.0YR 6/8), slightly thicker at the base. Odorless. Spore prints pink.

Basidiospores [80, 3, 2] 6.0–6.5(–7.0) × 5.0–5.5(–6.0) μm, avL × avW = 6.2–6.5 × 5.2–5.5 µm, Q = 1.16–1.40 μm, avQ = 1.20–1.26 μm, broadly ellipsoid to ellipsoid, slightly pinkish, smooth, thin-walled. Basidia 20–28 × 7–9 μm, clavate, thin-walled, 4-sterigmate, and hyaline. Pleurocystidia 49–95 × 22–33 μm, abundant, scattered, fusiform to flask-shaped, apically obtusely rounded or partially with 1–2 digitiform excrescences at apex, thinly walled, smooth, hyaline. Lamellar edge sterile. Cheilocystidia 41–70 × 16–25 μm, numerous, fusiform to narrowly clavate, or clavate, apically with a mucronate or rostrate, thin-walled, mostly with brown intracellular pigment. Pileipellis a trichohymeniderm composed of narrowed fusiform and clavate elements, terminal elements expanded to a spindle to clavate 62–140 long, 27–36 μm wide, thin-walled, with brown intracellular pigment. Stipitipellis a cutis, hyphae 5–10 µm diam, cylindrical, hyaline, thin-walled. Caulocystidia 53–108 × 11–22 μm, numerous, usually in clusters, spindle, or cylindrical to narrowly lageniform, rounded at apex, with brown to pale brown intracellular pigment, slightly thick and smooth walls. Clamp connections absent in all tissues.

###### Habitat.

Scattered on decaying wood in coniferous forests (*P.koraiensis*).

###### World distribution.

China.

###### China distribution.

Heilongjiang Province, Jilin Province.

###### Additional specimens examined.

CHINA • Jilin Province, Cold Jungle National Nature Reserve; Solitary on rotting wood in coniferous forests; 2 August 2020, G. Rao, FJAU66618 (Collection no.: Rao 938) (ITS: PQ810769, LSU: PQ810757, *tef1*: PQ811052).

###### Notes.

*Pluteuscostatus* is primarily characterized by its pileus with prominent veins, particularly a distinctive pronounced middle vein, and cheilocystidia with mucronate apices.

This species shares morphological similarities with *P.ornatus* and *P.umbrosus*, but detailed comparative analyses reveal several distinguishing features. *P.costatus* is differentiated from *P.ornatus* by its unstriped pileus margins, smaller basidiospores (avL × avW = 6.2–6.5 × 5.2–5.5 µm), substrate preference for decaying gymnosperm wood (*Pinus*), and distribution in East Asia. In contrast, *P.ornatus* exhibits striate pileus margins, larger basidiospores (avL × avW = 7.7 × 6.3 µm), preference for decaying angiosperm wood (associated with *Fagaceae*, *Dilleniaceae*, *Theaceae*, with the significant presence of *bamboos*, *Calamus*, and *Rubus*), and distribution in Vietnam. This distinction is further supported by an ITS genetic distance of 0.029 (SE = 0.007) between these taxa (Malysheva et al. 2023).

Phylogenetically, our analyses demonstrate that the two specimens of *P.costatus* form a well-supported monophyletic branch (MLB = 91, BPP = 1, Fig. 3) sister to *P.umbrosus* and *P.granularis*. *P.costatus* can be distinguished from these sister taxa by lamellae edge pigmented: white to light brown in *P.costatus*, distinctly brown in *P.umbrosus*, and not pigmented in *P.granularis* (Kaygusuz et al. 2019).

##### ﻿ Pluteus umbrosus

Taxon classificationFungiAgaricalesPluteaceae

(Pers.) P. Kumm., Führ. Pilzk. (Zerbst) (1871: 98)

B6C6604D-B203-507D-818D-D2DA1D675417

Figs 18D, E , 20

###### Description.

Basidiomata large-sized. Pileus 50–52 mm diam; convex to hemispherical, with a low, broad umbo; surface reddish-brown (7.5YR 7/8), distributed with granular tan (7.5YR 5/10), dense and dark brown in the center (7.5YR 5/14), forming vein-like striate; margin crenulate, with small rounded or blunt teeth. Lamellae reddish-brown (2.5R 5/6), free, crowded, thick, unequal, slightly ventricose, 5–7 mm wide, with brown and even to flocculose edges; lamellar edge commonly with brown pigment. Stipe 43–48 × 5–8 mm, cylindrical, gradually thickening toward the base, fibrous, surface reddish-brown with tan granules (2.5YR 6/10). Odorless. Spore prints pink.

Basidiospores [200, 4, 1] 5.0–6.0(–7.0) × 4.5–5.0 μm, avL × avW = 5.8–6.0 × 5.3–5.6 µm, Q = 1.05–1.33 μm, avQ = 1.10–1.16 μm, subglobose to broadly ellipsoid, slightly pinkish, smooth, thin-walled. Basidia 26–30 × 6–10 μm, clavate, thin-walled, 4-sterigmate, and hyaline. Pleurocystidia 55–90 × 22–37 μm, abundant, scattered, fusiform to lageniform, often with tapering apex, or mucronate, 5–17 μm long, thin-walled, smooth, hyaline. Lamellar edge sterile. Cheilocystidia 41–75 × 14–20 μm, numerous, clustered, subfusiform to fusiform, or ventricose, with small irregular apical horns, 4–13 μm long, thin-walled, generally with brown intracellular pigment. Pileipellis a trichohymeniderm composed of narrowed fusiform and clavate elements, with terminal elements (51–)62–170(–190) × 9–27 μm, often grouped in clusters, pointed towards the apex, with brown intracellular pigment, thin and smooth. Stipitipellis a cutis, hyphae 5–13 µm diam, cylindrical, hyaline, thin-walled. Caulocystidia 53–125 × 10–24 μm, numerous, usually in clusters, cylindrical to slightly narrowly lageniform, rounded at apex, with brown to pale brown intracellular pigment, slightly thick-walled. Clamp connections absent in all tissues.

###### Habitat.

Scattered on fallen wood with *Sphagnum* growing in broad-leaved forests.

###### World distribution.

Denmark, Finland, Norway, Sweden (Heilmann-Clausen 2012), Germany (Krieglsteiner 2003; Ludwig 2007), Britain and Ireland (Orton 1986; Legon et al. 2005), Italy (Boccardo et al. 2008), Switzerland (Breitenbach and Kränzlin 1995), Netherlands (Vellinga 1990), Spain (Justo et al. 2011a), Russia (Malysheva et al. 2016), Turkey (Kaygusuz et al. 2019), China (Xu 2016).

###### China distribution.

Northeast, North, Northwest, Southwest China.

###### Additional specimens examined.

CHINA • Jilin Province, Changbai Mountain Old Mountain Gate; Scattered on fallen wood with Sphagnum growing in broad-leaved forests; 6 September 2019, J.J. Hu, FJAU66590 (Collection no.: Hu 5140) (ITS: PP516604, *tef1*: PP928982).

###### Notes.

*Pluteusumbrosus* is characterized by a texture on the pileus and stipe that is granular like fine flour or velvety with dark brown radial fibrils, a pileus center with a generally dark veined pattern, a brown flocculose stipe, and lamellae with a distinct brown edge (Singer 1956; Orton 1986; Vellinga 1990; Kaygusuz et al. 2019).

The general morphological characteristics are similar to those of *P.umbrosoides* and *P.granularis*. However, morphologically, *P.umbrosus* has a brown lamellae edge, while the mature *P.umbrosoides* and *P.granularis* do not have a pigmented lamella edge (Kaygusuz et al. 2019). Microscopically, *P.umbrosus* differs from *P.granularis* in having a slightly wider basidiospore size of 5.8–6.0 × 5.3–5.6 μm and very long pileipellis elements reaching over 270 μm. *P.umbrosus* has a pleurocystidia with 1–2 horns without apical mucilage, while *P.granularis* has a pleurocystidia with 1–3 horns with distinctly apical mucilage. Additionally, *P.umbrosus* can be distinguished from *P.granularis* by its caulocystidia, which have a broadly rounded apex (up to 27 μm in width) (Kaygusuz et al. 2019).

The phylogeny shows that *P.umbrosus* from China clustered in the same branch as *P.umbrosus* from Italy, Russia, and Turkey with high support (MLB = 93, BPP = 0.92, Fig. 3). *P.umbrosus* is most closely related to *P.granularis*, they are sister groups, and their ITS genetic distance is 0.005 (SE = 0.003), *tef1* genetic distance is 0.006 (SE = 0.003).

##### ﻿ Pluteus umbrosoides

Taxon classificationFungiAgaricalesPluteaceae

E.F. Malysheva, Mycol. Progr. 15: 880 (2016)

FD56B704-813F-5616-A2FE-299299C369EB

Figs 18F–H , 21

###### Description.

Basidiomata medium-sized. Pileus 15–41 mm diam; plano-convex to convex; earthy yellow (2.5Y 8/6), surface with black-brown (7.5YR 3/2) recurved conical scales, denser in the middle forming a vein, becoming sparse toward the margin, margin straight and slightly crenulate. Context white (10P 9/2), 3–6 mm thick. Lamellae free, 5–7 mm wide, crowded, white (10P 9/2) to pink (7.5RP 8/4), unequal, moderately thick, ventricose, edges even to flocculose and white. Stipe 21–76 × 5–9 mm, fibrous, solid, clavate, slightly expanded at the base, whitish overall (10P 9/2), with brown (7.5YR 4/4) glandular dots on the surface. Spore print unknown.

Basidiospores [40/2/1] 5.5–6.5(–7.0) × 5.0–6.0 µm, avL × avW = 6.1–6.5 × 5.5–5.9 µm, Q = 1.18–1.30 µm, avQ = 1.20–1.25 µm, broadly ellipsoid to ellipsoid, smooth, slightly pinkish, thick-walled. Basidia 24–28 × 7–9 μm, clavate, thin-walled, 4-sterigmate, hyaline. Pleurocystidia 58–82 × 21–35 μm, rare, scattered, fusiform to broadly fusiform, with shorter necks, thin-walled, smooth, hyaline. Lamellar edge sterile. Cheilocystidia 37–63 × 16–21 μm, abundant, clustered, similar to Pleurocystidia, fusiform, commonly mucronate, thin-walled, smooth, generally with brown intracellular pigment. Pileipellis a trichohymeniderm composed of narrowed fusiform and clavate elements, with terminal elements 71–155(–174) × 17–25 μm, often grouped in clusters, pointed towards the apex, with brown intracellular pigment, thin- or slightly thick-walled. Stipitipellis a cutis, hyphae 4–7 μm diam, cylindrical, hyaline, thin-walled. Without caulocystidia. Clamp connections absent in all tissues.

###### Habitat.

Scattered on very rotten decaying wood in broad-leaved forests (*Q.mongolica*).

###### World distribution.

Russia (Malysheva et al. 2016), China (Hosen et al. 2018), Turkey (Kaygusuz et al. 2019).

###### China distribution.

Jilin Province.

###### Additional specimens examined.

CHINA • Jilin Province, Cold Onion Ridge Forest Park. Scattered on very rotten decaying wood in broad-leaved forests (*Q.mongolica*); 25 August 2019, G. Rao, FJAU66615 (Collection no.:Rao 614) (ITS: PQ810780, LSU: PQ810741).

###### Notes.

Malysheva et al. reported that *P.umbrosoides* is characterized by pink lamellae without brown margin, a densely squamose pileus which is veined around the center, a smooth stipe without brown squamules, ellipsoid basidiospores, pleurocystidia commonly capitate in shape, a trichohymeniderm pileipellis, and narrowly fusiform caulocystidia (Malysheva et al. 2016).

Morphologically, the specimens from China are very similar to the type. Alternatively, we identified one major difference among specimens from China which is the absence of caulocystidia. Even though we observed the upper, middle, and basal parts of the stipe, exhibiting similarity with Hosen et al. (2018) specimens taken from Changbai Mountain, China, neither of which observed caulocystidia.

In the molecular phylogenetic tree, the Chinese specimens clustered in the same branch with *P.umbrosoides* from Russia, China, and Turkey and had high support (MLB = 100, BPP = 1, Fig. 3).

##### ﻿ Pluteus granularis

Taxon classificationFungiAgaricalesPluteaceae

Peck, Ann. Rep. N.Y. St. Mus. Nat. Hist. (1885: 135)

AB5DA5C2-4939-54F1-9A57-87FE1FC22032

Figs 18I, J , 22

###### Description.

Basidiomata medium to large-sized. Pileus 30–51 mm diam; plano-convex to convex, slightly obtuse raised in the middle; yellow (10YR 6/6) to yellow-brown (10YR 5/6), with brown (5YR 3/6) warped scales attached to the surface, the middle scales densely forming a vein-like, gradually becoming sparser toward the margin, with triangular scales toward the margin. Context white (10P 9/2) and odorless. Lamellae free, 4–6 mm wide, crowded, yellowish-brown (2.5YR 4/6), unequal, thin, ventricose, white and even to flocculose edges. Stipe 32–95 × 3–8 mm, fibrous, clavate, hollow, slightly expanded at the base, white (10P 9/2) overall, with denser brown (10YR 3/6) cilia on the middle to lower portion. Spore print unknown.

Basidiospores [60/2/1] 5.5–6.0(–6.5) × 4.5–5.0 µm, avL × avW = 5.7–6.0 × 4.6–4.8 µm, Q = 1.10–1.30 µm, avQ = 1.20 µm, subglobose, broadly ellipsoid to ellipsoid, smooth, slightly pinkish, thick-walled. Basidia 23–28 × 8–11 μm, broadly clavate, thin-walled, 4-sterigmate, and hyaline. Pleurocystidia 55–83 × 15–33 μm, scattered, rare, two forms, one fusiform, with apical finger-like projections, 3–6 μm long, the other narrowly clavate to clavate, with an obtusely rounded apical portion, thin-walled, smooth, partly with brown intracellular pigment. Lamellar edge sterile. Cheilocystidia 34–65 × 13–22 μm, abundant, clustered, broadly clavate to clavate, apically obtuse, thin-walled, smooth, hyaline. Pileipellis a trichohymeniderm composed of narrowed fusiform and clavate elements, with terminal elements 130–216 × 17–31 μm, often aggregated into bundles, pointed towards the apex, with brown intracellular pigment, thin-walled, smooth. Stipitipellis a cutis, hyphae 4–6 μm diam, cylindrical, hyaline, thin-walled. Caulocystidia 36–55 × 13–19 μm, scattered, composed of long clavate to clavate vesicles, partly containing brownish pigment, thin-walled, smooth. Clamp connections absent in all tissues.

###### Habitat.

Scattered on very rotten decaying wood in broad-leaved forests (*Q.mongolica*).

###### World distribution.

USA (Peck 1885, 1899; Murrill 1917; Kauffman 1918; Bessette et al. 1997; Justo et al. 2011b), Turkey (Kaygusuz et al. 2019), China (Yang 2011).

###### China distribution.

Jilin Province, Sichuan Province, Yunnan Province, Tibet Autonomous Region (Yang 2011).

###### Additional specimens examined.

CHINA • Jilin Province, Quanyang Township, Beigang, latitude and longitude: 43°02'2.06"N, 127°58'44.69"E; Scattered on very rotten decaying wood in broad-leaved forests (*Q.mongolica*); 22 August 2021, Z.X. Qi, FJAU66612 (Collection no.: Qi 395) (ITS: PQ810758, LSU: PQ810735, *tef1*: PQ811045).

###### Notes.

Peck reported *P.granularis* in 1885, and the species has been reported by several people since (Peck 1885, 1899; Murrill 1917; Kauffman 1918; Bessette et al. 1997; Justo et al. 2011b; Xu 2016; Kaygusuz et al. 2019). Summarizing their description of *P.granularis*, it can be found that it is characterized by an intense dark brown granular stipe and predominantly with conspicuous rugose-wrinkled, granulose pileus, pleurocystidia with apical mucilage. Our specimens are overall similar to their descriptions, both having an intense dark brown granular stipe and predominantly with conspicuous rugose-wrinkled, granulose pileus, but there is a slight difference. The latter pleurocystidia vesicles frequently form 1–3 small irregular horns apically and, if hornless, usually have mucilage apically. In the former, only 1 small irregular horn is observed and there is no mucilage.

Morphologically, *P.granularis* is very similar to *P.umbrosus*, as discussed in detail in the notes on *P.umbrosus*. The phylogeny shows that *P.granularis* from China clustered in the same branch as *P.granularis* from the USA with high support (MLB = 98, BPP = 1, Fig. 3). Here we report *P.granularis* from China, a common species.

#### ﻿/*leoninus* clade

(Figs 23–30)

**Figure 23.**
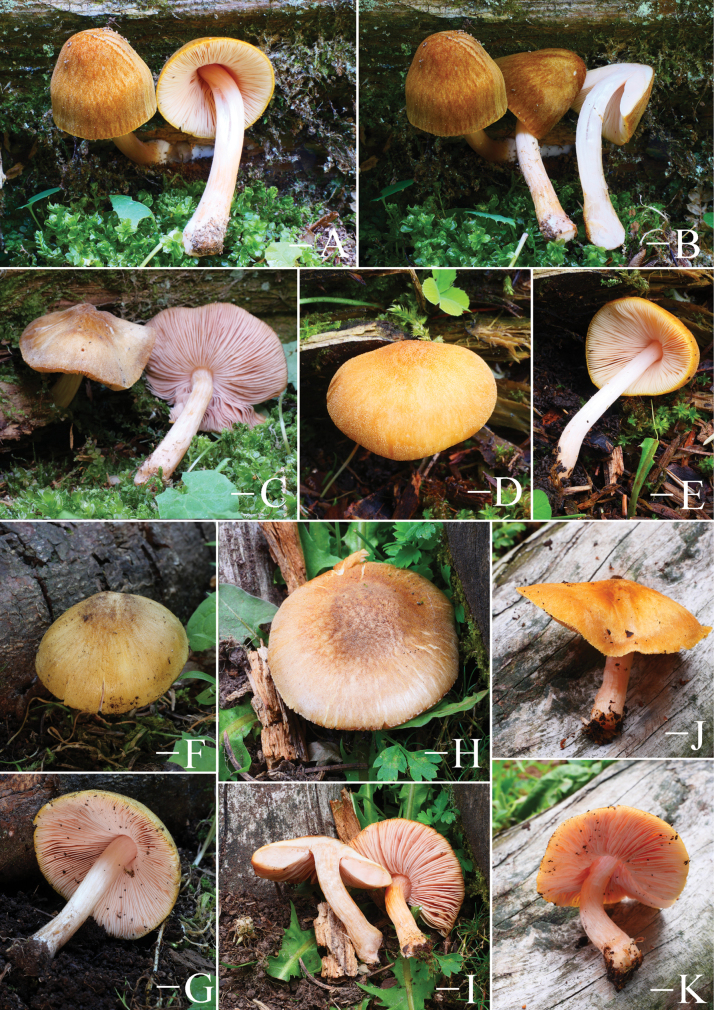
Basidiomata features. A–K. *Pluteuspiceicola* (A, B. FJAU66574, C. FJAU66569, D, E. FJAU66575, F, G. FJAU66571, H, I. FJAU66573, J, K. FJAU66572). A–K. Photos by Zheng-Xiang Qi. Scale bars: 1 cm.

**Figure 24.**
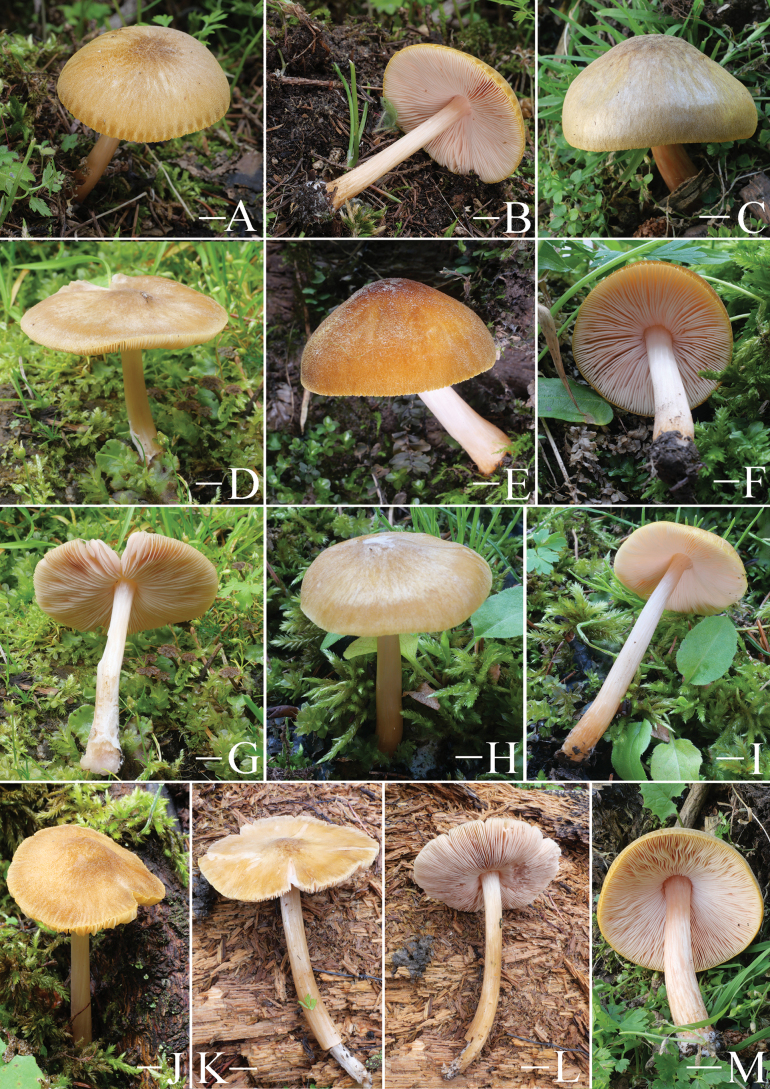
Basidiomata features. A–M. *Pluteuspiceicola* (A, B. FJAU66603, C, M. FJAU66607, D, G. FJAU66605, E, F. FJAU66606, H, I. FJAU66608, J. FJAU66610, K, L. FJAU66609). A–M. Photos by Zheng-Xiang Qi. Scale bars: 1 cm.

**Figure 25.**
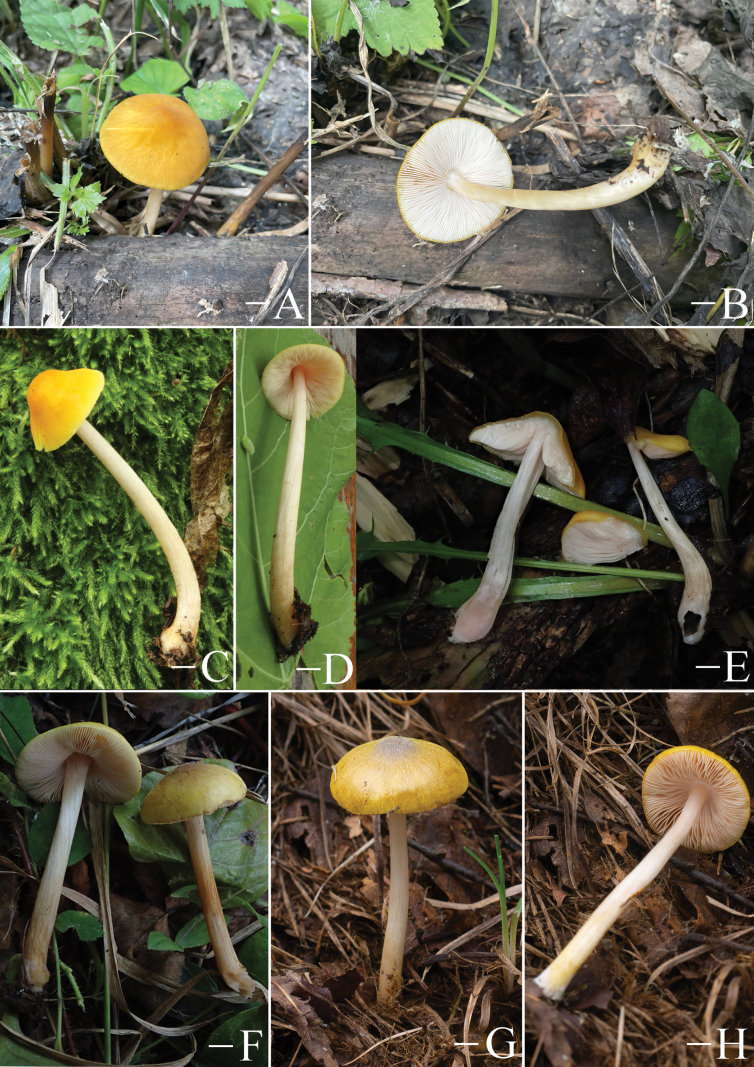
Basidiomata features. A–E. *Pluteusussuriensis* (A, B. FJAU66576, C, D. FJAU66577, E. FJAU66578). F–H. *P.leoninus* (F. FJAU66581, G, H. FJAU66580). A–D. Photos by Gu Rao. E. Photos by Ming-Hao Liu. F. Photos by Di-Zhe Guo. G, H. Photos by Zheng-Xiang Qi. Scale bars: 1 cm.

**Figure 26.**
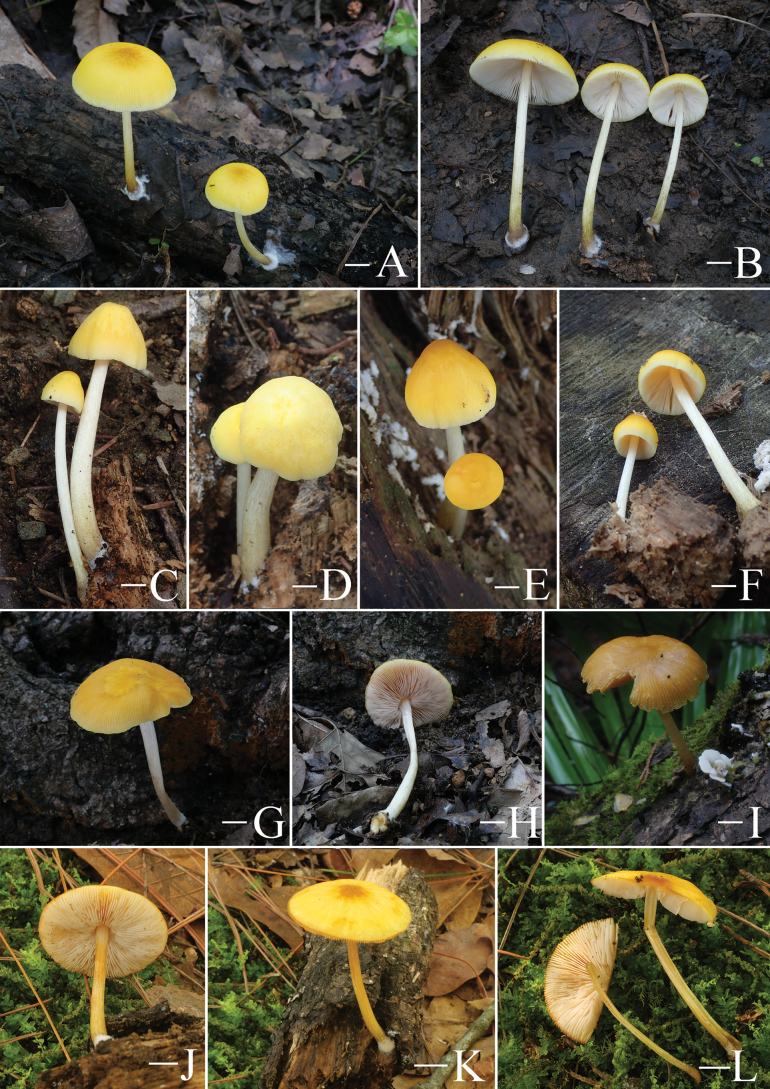
Basidiomata features. A–L. *Pluteusvariabilicolor* (A, B. FJAU66585, C, D. FJAU66584, E, F. FJAU66583, G, H. FJAU66586, I. FJAU66588, J–L. FJAU66623). A–H. Photos by Ya-Jie Liu. I–L. Photos by Li-Bo Wang. Scale bars: 1 cm.

**Figure 27.**
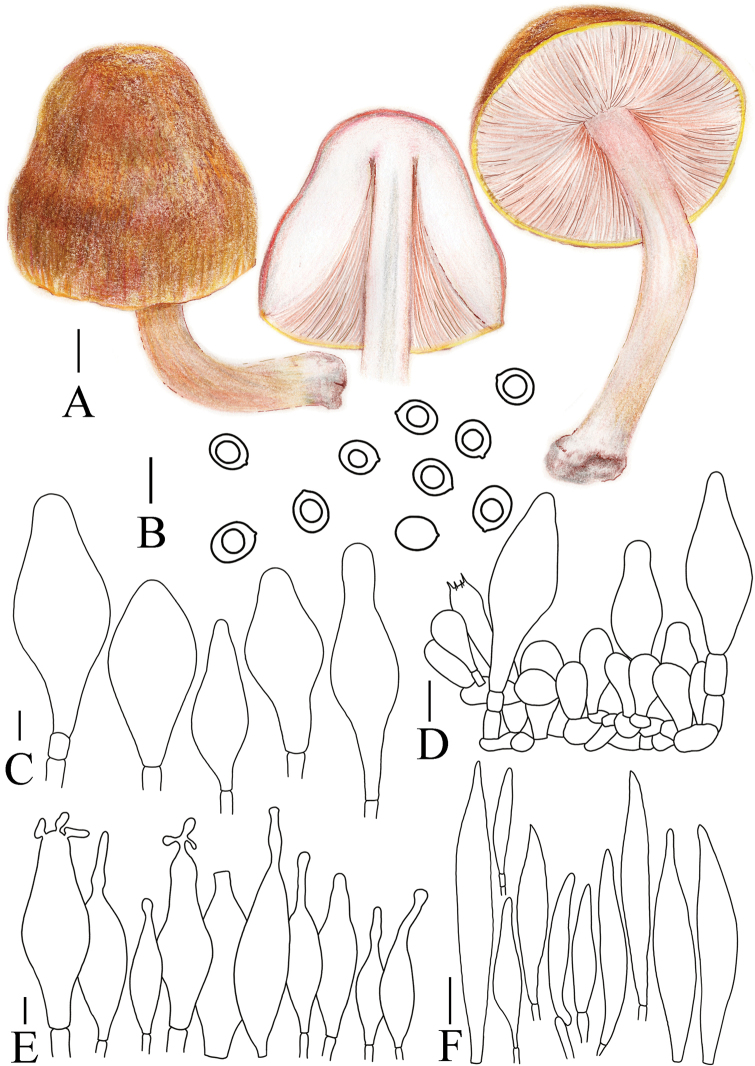
A. Macroscopic characteristics of *Pluteuspiceicola*. B. Basidiospores. C. Pleurocystidia. D. Basidia and Pleurocystidia. E. Cheilocystidia. F. Pileipellis elements. Scale bars: 1 cm (A); 10 µm (B–E); 20 µm (F).

**Figure 28.**
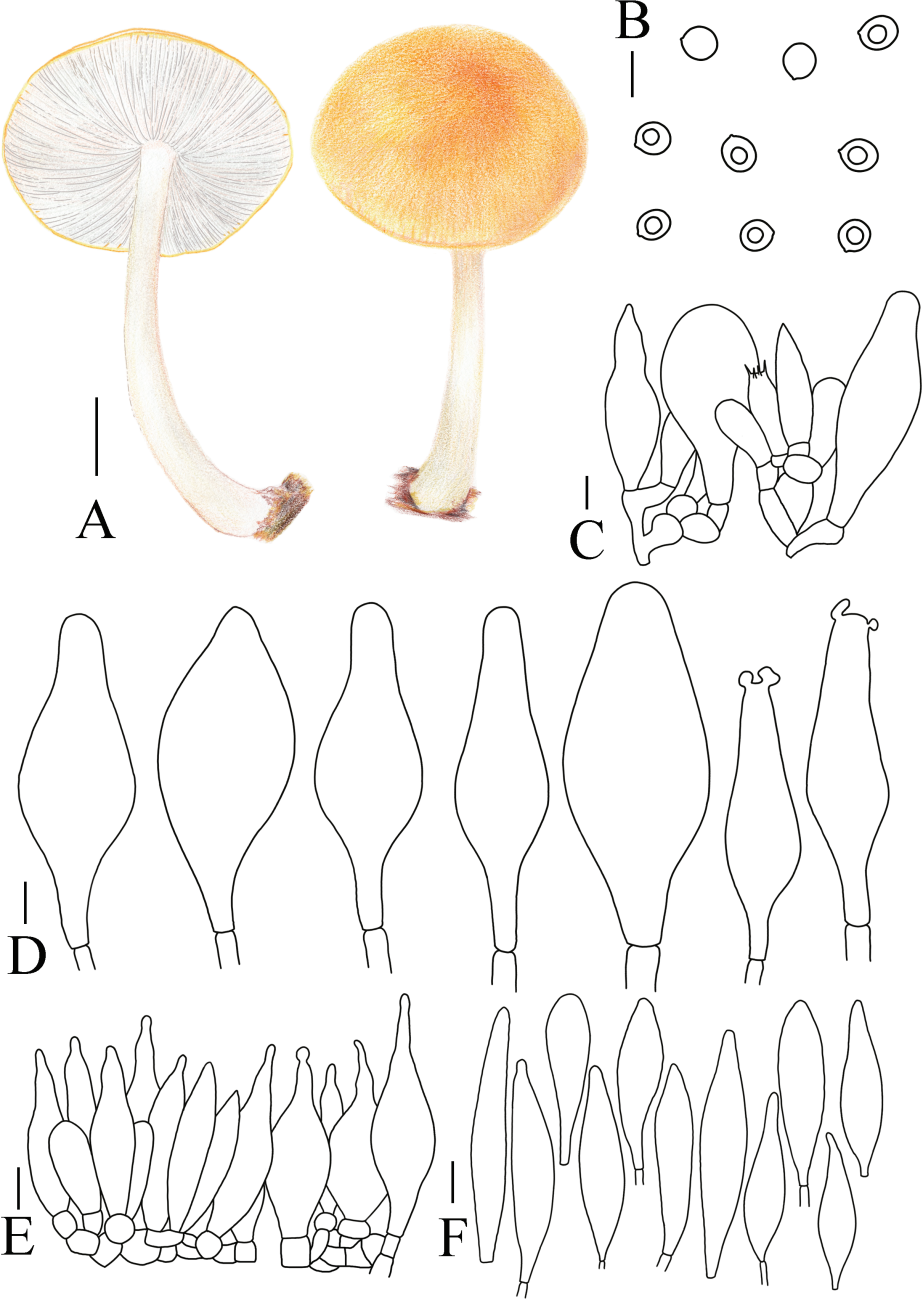
A. Macroscopic characteristics of *Pluteusussuriensis*. B. Basidiospores. C. Basidia and Pleurocystidia. D. Pleurocystidia. E. Cheilocystidia. F. Pileipellis elements. Scale bars: 1 cm (A); 10 µm (B–E); 20 µm (F).

**Figure 29.**
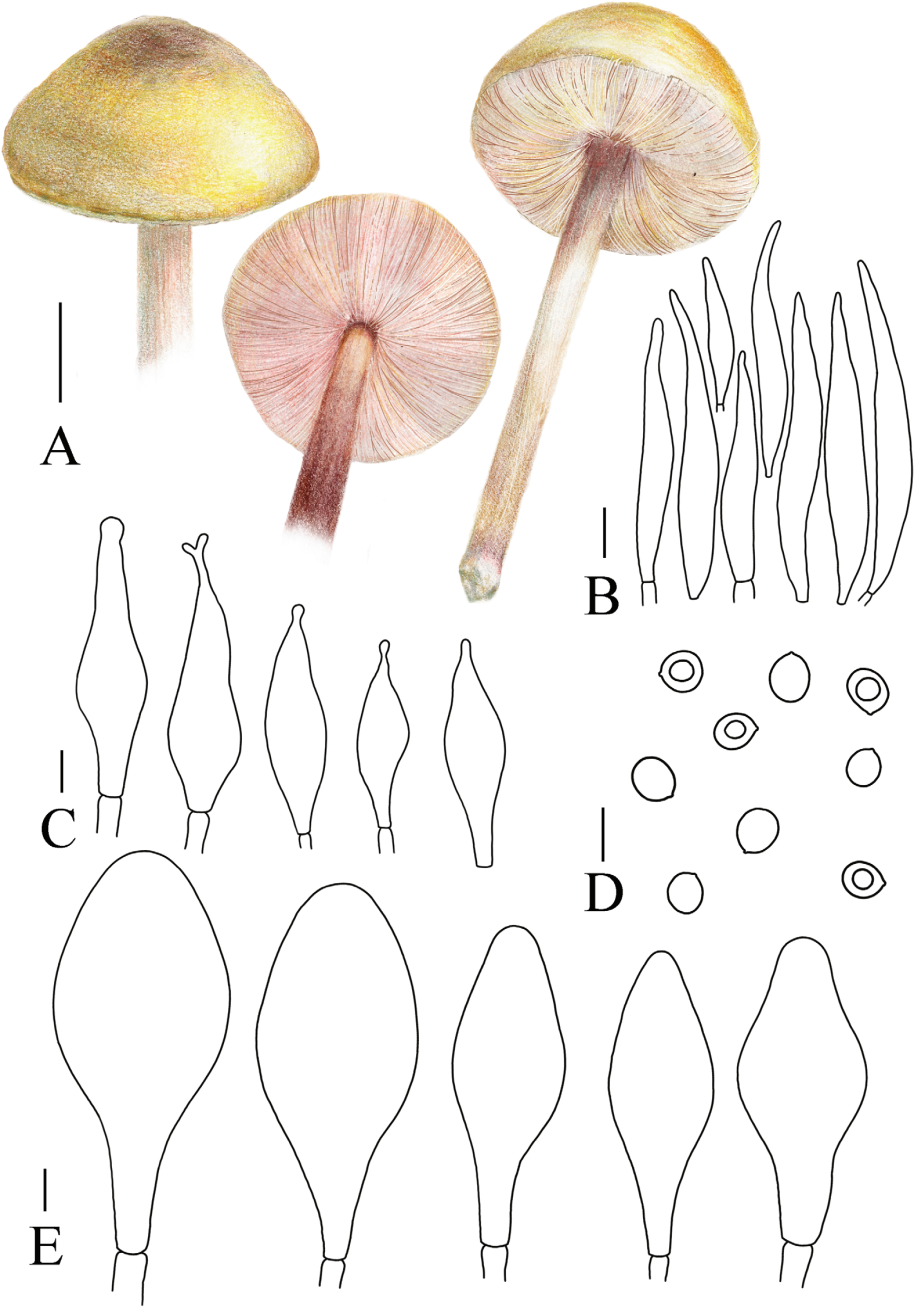
A. Macroscopic characteristics of *Pluteusleoninus*. B. Pileipellis elements. C. Cheilocystidia. D. Basidiospores. E. Pleurocystidia. Scale bars: 1 cm (A); 10 µm (B, C, E); 20 µm (D).

**Figure 30.**
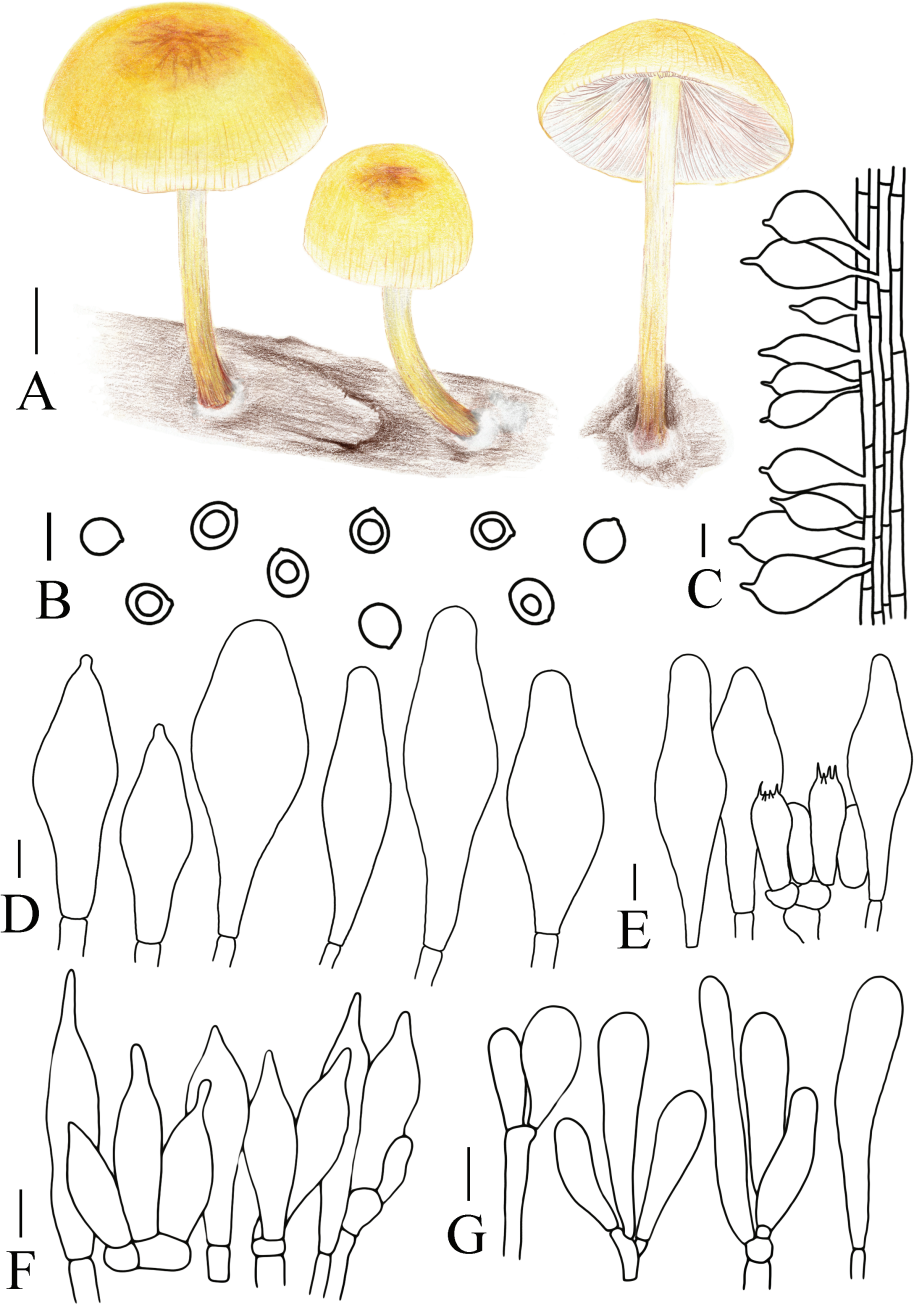
A. Macroscopic characteristics of *Pluteusvariabilicolor*. B. Basidiospores. C. Caulocystidia. D. Pleurocystidia. E. Basidia and Pleurocystidia. F. Cheilocystidia. G. Pileipellis elements. Scale bars: 1 cm (A); 10 µm (C–F); 20 µm (B, G).

##### ﻿ Pluteus piceicola

Taxon classificationFungiAgaricalesPluteaceae

Z.X. QI, B. Zhang & Y. Li sp. nov.

7D4C12CF-839A-5D77-9D0C-02CF8DB52A36

MycoBank No: 853185

Figs 23A–K , 24A–M , 27

###### Etymology.

The species epithet “piceicola” (Latin) refers to its habitat on *Piceaschrenkiana*.

###### Diagnosis.

Distinguished from *P.leoninus* by its pileus with a brown pruinose on the surface and the presence of spiral longitudinal striate of the stipe. On the phylogenetic tree, there are separate branches and its ITS sequences (genetic distance = 0.021, SE = 0.006) and *tef1* sequences (genetic distance = 0.090, SE = 0.015). It grows preferentially in coniferous forests (*Picea*) on decaying wood branches and is distributed in East Asia (China).

###### Holotype.

CHINA • Xinjiang Uygur Autonomous Region, Ili Kazakh Autonomous Prefecture, Tekes County, Jongkushtai village, 42°97'26.51"N, 82°12'77.25"E, alt. 2242 m, 3 September 2023, Z.X. Qi, FJAU66574 (ITS: PP516599, LSU: PP516651, *tef1*: PP551589) (Collection no.: Qi 2849).

###### Description.

Basidiomata medium to large-sized. Pileus 39–71 mm diam; campanulate to hemispherical when young, surface yellow-brown (10.0YR 8/20), with yellow-brown crumbly scales, central scales crowded; gradually plano-convex to convex at maturity, central obtuse umbo, earthy yellow to orange-yellow (10.0YR 8/10–10.0YR 7/14), surface brown pruinose, margin yellow (10.0YR 8/10). Context dirt white to yellowish (10.0YR 8/2–10.0YR 8/4), odorless, 5–8 mm thick. Lamellae initially dirty white (10.0YR 8/4), turning pink at maturity (2.5Y 8/12), free, crowded, thick, unequal, slightly ventricose, 6–7 mm wide, edges even. Stipe 47–71 × 8–13 mm, cylindrical, hollow, slightly thicker at the base, fibrous, some surfaces with brown longitudinal striate (7.5YR 7/14). Odorless. Spore prints pink.

Basidiospores [200, 30, 17] (–6.5)7.0–8.0(–8.5) × 5.5–6.0(–6.5) μm, avL × avW = 7.4–7.6 × 5.6–6.0 µm, Q = 1.16–1.54 μm, avQ = 1.20–1.25 μm, ellipsoid to broadly ellipsoid, slightly pinkish, smooth, thin-walled. Basidia 25–32 × 7–11 μm, clavate, thin-walled, 4-sterigmate, and hyaline. Pleurocystidia 55–102 × 22–36 μm, numerous, scattered, fusiform to subfusiform, or clavate, apically mostly obtusely rounded, with or without 1–2 digitiform excrescences at apex, thin-walled, smooth, hyaline. Lamellar edge sterile. Cheilocystidia 41–79 × 18–29 μm, abundant, clustered, subfusiform to fusiform, mostly apically mucronate to rostrate, or diverticulate, 10–23 μm long, a few obtusely rounded, thin-walled, hyaline. Pileipellis a trichoderm with radial hyphae, terminal elements 62–171 × 17–32 μm, cylindrical or fusiform elements, thick-walled, and slightly tan intracellular pigment. Stipitipellis a cutis, hyphae 5–13 µm diam, cylindrical, hyaline, thin-walled. Caulocystidia absent. Clamp connections absent in all tissues.

###### Habitat.

Solitary or scattered on decaying dead wood in spruce forests (*P.schrenkiana*).

###### World distribution.

China.

###### China distribution.

Xinjiang Uygur Autonomous Region.

###### Other specimens examined.

CHINA • Xinjiang Uygur Autonomous Region, Ili Kazakh Autonomous Prefecture, Tekes County, Jongkushtai village. Scattered on rotting wood in spruce forests (*P.schrenkiana*); 42°17'62.61"N, 82°51'98.22"E, alt. 2236 m, 19 July 2023, Z.X. Qi, FJAU66569 (Collection no.: Qi 498) (ITS: PP516594, LSU: PP516646, *tef1*: PP551584). CHINA • Xinjiang Uygur Autonomous Region, Ili Kazakh Autonomous Prefecture, Tekes County, Jongkushtai village; Solitary on rotting wood in spruce forests (*P.schrenkiana*); 42°19'62.68"N, 82°51'91.82"E, alt. 2128 m, 23 July 2023, Z.X. Qi, FJAU66570 (Collection no.: Qi 632) (ITS: PP516595, LSU: PP516647, *tef1*: PP551585). CHINA • Xinjiang Uygur Autonomous Region, Ili Kazakh Autonomous Prefecture, Tekes County, Jongkushtai village; Scattered on rotting wood in spruce forests (*P.schrenkiana*); 42°15'68.67"N, 82°79'93.88"E, alt. 2224 m, 2 August 2023, Z.X. Qi, FJAU66571 (Collection no.: Qi 840) (ITS: PP516596, LSU: PP516648, *tef1*: PP551586). CHINA • Xinjiang Uygur Autonomous Region, Ili Kazakh Autonomous Prefecture, Tekes County, Jongkushtai village; Solitary on rotting wood in spruce forests (*P.schrenkiana*); 42°15'11.73"N, 82°59'52.78"E, alt. 2318 m, 15 August 2023, Z.X. Qi, FJAU66572 (Collection no.: Qi 2125) (ITS: PP516597, LSU: PP516649, *tef1*: PP551587). CHINA • Xinjiang Uygur Autonomous Region, Ili Kazakh Autonomous Prefecture, Tekes County, Jongkushtai village; Solitary on rotting wood in spruce forests (*P.schrenkiana*), 42°19'18.93"N, 82°58'22.18"E, alt. 2241 m, 27 August 2023, Z.X. Qi, FJAU66573 (Collection no.: Qi 2584) (ITS: PP516598, LSU: PP516650, *tef1*: PP551588). CHINA • Xinjiang Uygur Autonomous Region, Ili Kazakh Autonomous Prefecture, Tekes County, Jongkushtai village; Solitary on rotting wood in spruce forests (*P.schrenkiana*); 42°15'28.45"N, 82°55'22.78"E, alt. 2169 m, 5 September 2023, Z.X. Qi, FJAU66575 (Collection no.: Qi 2925) (ITS: PP516600, LSU: PP516652, *tef1*: PP551590). CHINA • Xinjiang Uygur Autonomous Region, Ili Kazakh Autonomous Prefecture, Tekes County, Jongkushtai village; Solitary on rotting wood in spruce forests (*P.schrenkiana*); 42°15'27.07"N, 82°55'22.66"E, alt. 2487 m, 15 June 2024, Z.X. Qi, FJAU66601 (Collection no.: Qi 3179) (ITS: PQ810770, LSU: PQ810747, *tef1*: PQ811053). CHINA • Xinjiang Uygur Autonomous Region, Ili Kazakh Autonomous Prefecture, Tekes County, Jongkushtai village; Solitary on rotting wood in spruce forests (*P.schrenkiana*); 42°15'28.32"N, 82°55'21.28"E, alt. 2441 m, 23 June 2024, Z.X. Qi, FJAU66602 (Collection no.: Qi 3321) (ITS: PQ810771, LSU: PQ810748, *tef1*: PQ811054). CHINA • Xinjiang Uygur Autonomous Region, Ili Kazakh Autonomous Prefecture, Tekes County, Jongkushtai village; Solitary on rotting wood in spruce forests (*P.schrenkiana*); 42°15'27.29"N, 82°55'22.80"E, alt. 2159 m, 27 June 2024, Z.X. Qi, FJAU66603 (Collection no.: Qi 3383) (ITS: PQ810772, LSU: PQ810749, *tef1*: PQ811055). CHINA • Xinjiang Uygur Autonomous Region, Ili Kazakh Autonomous Prefecture, Tekes County, Jongkushtai village; Solitary on rotting wood in spruce forests (*P.schrenkiana*); 42°15'28.77"N, 82°55'20.35"E, alt. 2432 m, 27 June 2024, Z.X. Qi, FJAU66604 (Collection no.: Qi 3387) (ITS: PQ810773, LSU: PQ810750, *tef1*: PQ811056). CHINA • Xinjiang Uygur Autonomous Region, Ili Kazakh Autonomous Prefecture, Tekes County, Jongkushtai village; Solitary on rotting wood in spruce forests (*P.schrenkiana*); 42°15'27.77"N, 82°55'22.75"E, alt. 2333 m, 10 July 2024, Z.X. Qi, FJAU66605 (Collection no.: Qi 3631) (ITS: PQ810774, LSU: PQ810751, *tef1*: PQ811057). CHINA • Xinjiang Uygur Autonomous Region, Ili Kazakh Autonomous Prefecture, Tekes County, Jongkushtai village; Solitary on rotting wood in spruce forests (*P.schrenkiana*); 42°15'28.50"N, 82°55'23.60"E, alt. 2511 m, 11 July 2024, Z.X. Qi, FJAU66606 (Collection no.: Qi 3668) (ITS: PQ810775, LSU: PQ810752, *tef1*: PQ811058). CHINA • Xinjiang Uygur Autonomous Region, Ili Kazakh Autonomous Prefecture, Tekes County, Jongkushtai village; Solitary on rotting wood in spruce forests (*P.schrenkiana*); 42°15'27.32"N, 82°55'22.66"E, alt. 2493 m, 20 July 2024, Z.X. Qi, FJAU66607 (Collection no.: Qi 3961) (ITS: PQ810776, LSU: PQ810753, *tef1*: PQ811059). CHINA • Xinjiang Uygur Autonomous Region, Ili Kazakh Autonomous Prefecture, Tekes County, Jongkushtai village; Solitary on rotting wood in spruce forests (*P.schrenkiana*); 42°15'28.47"N, 82°55'22.55"E, alt. 2182 m, 2 August 2024, Z.X. Qi, FJAU66608 (Collection no.: Qi 4133) (ITS: PQ810777, LSU: PQ810754, *tef1*: PQ811060). CHINA • Xinjiang Uygur Autonomous Region, Ili Kazakh Autonomous Prefecture, Tekes County, Jongkushtai village; Solitary on rotting wood in spruce forests (*P.schrenkiana*); 42°15'28.71"N, 82°55'22.44"E, alt. 2362 m, 30 August 2024, Z.X. Qi, FJAU66609 (Collection no.: Qi 4849) (ITS: PQ810778, LSU: PQ810755, *tef1*: PQ811061). CHINA • Xinjiang Uygur Autonomous Region, Ili Kazakh Autonomous Prefecture, Tekes County, Jongkushtai village; Solitary on rotting wood in spruce forests (*P.schrenkiana*); 42°15'28.20"N, 82°55'22.49"E, alt. 2251 m, 30 August 2024, Z.X. Qi, FJAU66610 (Collection no.: Qi 4856) (ITS: PQ810779, LSU: PQ810756, *tef1*: PQ811062).

###### Notes.

*Pluteuspiceicola* is characterized by its orange-yellow pileus with brown pruinose surface and distinctive spiral longitudinal stripes on the stipe. This species is associated with decaying wood in coniferous forests, specifically *Piceaschrenkiana*, and is distributed in East Asia (China).

Morphologically, *P.piceicola* may be confused with other *Pluteus* species exhibiting yellow to bright orange-yellow pileus. *P.piceicola* is distinguished from *P.leoninus* by its brown pruinose pileus surface, substrate preference for decaying woody branches in coniferous forests. In contrast, *P.leoninus* inhabits well-decayed wood of angiosperms (e.g., *Betula*, *Carpinus*, *Fagus*, *Quercus*), is occasionally terrestrial under hardwoods, and rarely occurs on conifer wood (*Abies*) (Kaygusuz et al. 2019; Justo et al. 2025). *P.piceicola* differs from *P.ussuriensis* primarily by its rougher, brown pruinose pileus surface, compared to the smoother pileus of the latter. This morphological distinction is supported by genetic distances of 0.034 (SE = 0.008) for ITS and 0.095 (SE = 0.016) for *tef1* (Justo et al. 2025).

*P.piceicola* can be separated from *P.admirabilis* and *P.chrysophaeus* by its predominantly white stipe versus the yellow to yellowish stipe of the latter taxa, as well as by pileipellis structure: trichoderm in *P.piceicola* versus hymeniderm in the latter species (Takehashi and Kasuya 2007; Menolli and Capelari 2010).

*P.piceicola* shares similarities with *P.favrei*, both exhibiting yellow to yellow-brown pileus, cheilocystidia with irregular or branched rostra, and a preference for decaying wood in *Picea* forests. However, *P.piceicola* produces smaller basidiospores (avL × avW = 7.4–7.6 × 5.6–6.0 µm) and lacks caulocystidia, while *P.favrei* forms larger basidiospores (avL × avW = 7.4–7.8 × 6.0–6.7 µm) and possesses caulocystidia (observed in the type collection). These taxa are further differentiated by genetic distances of 0.023 (SE = 0.006) for ITS and 0.078 (SE = 0.014) for *tef1* (Justo et al. 2025).

Phylogenetically, *P.piceicola* is firmly positioned within the /*leoninus* clade with strong statistical support (MLB = 100, BPP = 1, Fig. 4), forming a distinct branch with distant affinities to morphologically similar species such as *P.favrei*, *P.ussuriensis*, and *P.leoninus*.

##### ﻿ Pluteus ussuriensis

Taxon classificationFungiAgaricalesPluteaceae

E.F. Malysheva, in Justo, Malysheva, Bulyonkova, Muñoz, Ferisin, Dovana, Kaygusuz, Saar, Antonín, Vellinga, Lebeuf, Minnis, Grootmyers, Kalichman, Parker, Miller, Russell, Berbee, Pacholek, Ceska & Pradeep, Mycologia: 20 (2025)

22D64D10-5374-5A64-88B0-8770A4A2E274

Figs 25A–E , 28

###### Description.

Basidiomata medium-sized. Pileus 28–35 mm diam; campanulate when young, surface orange-red (7.5YR 8/16); plano-convex or convex when mature, bright yellow (2.5Y 8/12), slightly brownish (10.0YR 7/16); smooth, margin slightly translucently striate, with straight margin. Context dirty white (10.0YR 9/4), odorless, 3–5 mm thick. Lamellae initially dirty white (10.0YR 9/4), turning pink at maturity (10.0YR 8/8), free, crowded, thick, unequal, slightly ventricose, 6–7 mm wide. Stipe 68–72 × 8–10 mm, cylindrical, hollow, partly spirally curled, slightly thicker at the base, fibrous, white to brown at the base (10.0YR 9/10). Odorless. Spore prints pink.

Basidiospores [210, 7, 4] 6.0–6.5(–7.0) × 5.0–5.5(–6.0) μm, avL × avW = 6.3–6.5 × 5.2–5.4 µm, Q = 1.09–1.40 μm, avQ = 1.18–1.24 μm, broadly ellipsoid to ovoid, slightly pinkish, smooth, thin-walled. Basidia 25–32 × 7–11 μm, clavate, thin-walled, 4-sterigmate, and hyaline in KOH. Pleurocystidia 55–102 × 22–36 μm, numerous, scattered, fusiform to subfusiform, obtusely rounded apically, tapering basally, commonly provided with 1–2 digitiform excrescences at apex, thin-walled, smooth, hyaline. Lamellar edge sterile. Cheilocystidia 41–79 × 18–29 μm, abundant, clustered, subfusiform to fusiform, mostly apically mucronate to rostrate, 8–20 μm long, thin-walled, hyaline. Lamellar trama divergent. Pileipellis a trichoderm with radial hyphae, with terminal elements 62–143 × 14–29 μm, hyphae at the center extending outwards, with cylindrical or fusiform elements, thick-walled, with slightly tan intracellular pigment. Stipitipellis a cutis, hyphae 6–11 µm diam, cylindrical, hyaline, thin-walled. Caulocystidia absent. Clamp connections absent in all tissues.

###### Habitat.

Solitary on rotting wood in broad-leaved or mixed forests.

###### World distribution.

Russian Far East (Justo et al. 2025), China.

###### China distribution.

Jilin Province.

###### Additional specimens examined.

CHINA • Jilin Province, Cold Jungle National Nature Reserve; 5 August 2021, G. Rao, FJAU66576 (Collection no.: Rao 1287) (ITS: PP516606, LSU: PP516656, *tef1*: PP551592). CHINA • Jilin Province, Cold Jungle National Nature Reserve. Solitary on decaying wood in broad-leaved forests; 7 August 2021, G. Rao, FJAU66577 (Collection no.: Rao 1297) (ITS: PP516608, LSU: PP516657, *tef1*: PP551593). CHINA • Jilin Province, Hà Nội Việt Nam Conservation Station Front Hill; Scattered on rotting wood in mixed *Q.mongolica* and *U.pumila* forests; 42°15'27.75"N, 126°15'28.78"E, alt. 756 m, 18 July 2021, M.H. Liu, FJAU66578 (Collection no.: Lmh 045) (ITS: PP516607, LSU: PP516658, *tef1*: PP551594). CHINA • Jilin Province, Hà Nội Việt Nam Conservation Station Front Hill; Scattered on rotting wood in mixed *Q.mongolica* and *U.pumila* forests; 42°15'20.77"N, 126°10'78.88"E, alt. 825 m, 8 August 2021, M.H. Liu, FJAU66579 (Collection no.: Lmh 102) (ITS: PP516609, LSU: PP516659, *tef1*: PP551595).

###### Notes.

*Pluteusussuriensis* is characterized by its bright yellow and smooth pileus, white stipe that expanded at the base, and cheilocystidia that are rostrate at the top and measure 8–20 μm long.

Justo described *P.ussuriensis* in 2025, and we compared all the characters and found that morphologically (pileus, stipe, lamellae, basidiospores, pleurocystidia, pileipellis) are very similar, its sequence bases are the same, and the substrate ecology is on decaying wood in broad-leaved forests, and the distribution is in the Changbaishan Mountains. Meanwhile, we also observed some differences, such as the apical projection being longer in our collections of cheilocystidia, and the projection described by Justo et al. (2025) being shorter or more pointed; no caulocystidia were observed in our collections, and a few caulocystidia were observed by Justo et al. (2025); and there are 4 *tef1* base differences. We consider the similarities and differences together, and here identify the Chinese specimen as *P.ussuriensis*, a newly recorded species from China. However, the differences all belong to be part of the intraspecific variation, as Justo et al. (2025) describe the species in the /*leoninus* clade, and cheilocystidia vary considerably from specimen to specimen of the same species.

Phylogenetic analyses indicate that the four Chinese specimens cluster in the same branch as Justo et al. (2025)*P.ussuriensis* and are strongly supported. (MLB = 100, BPP = 1, Fig. 4). *P.ussuriensis* and *P.roseipes* are considered sister groups, and their distinction can be based on genetic distance differences and geographic distribution. Their ITS genetic distance is 0.012 (SE = 0.005), *tef1* genetic distance is 0.075 (SE = 0.014); *P.ussuriensis* is distributed in East Asia, and *P.roseipes* is distributed in the Czech Republic, Denmark, Estonia, France, Italy, Slovenia, Spain, and Turkey (Justo et al. 2011a, b; Pradeep et al. 2012; Lezzi et al. 2014; Menolli et al. 2015a; Ferisin and Dovana 2018; Kaygusuz et al. 2019; Justo et al. 2025).

##### ﻿ Pluteus leoninus

Taxon classificationFungiAgaricalesPluteaceae

(Schaeff.) P. Kumm., Führ. Pilzk. (Zerbst) (1871: 98)

75A12E12-838A-5BDF-BB69-E35B894B16B3

Figs 25F–H , 29

###### Description.

Basidiomata medium-sized. Pileus 20–31 mm diam; convex to plano-convex, often with a low, broad umbo; egg-yellow (5.0Y 9/12), black-brown in the center (2.5YR 5/8), with white pruinose on the surface, margin usually translucently striate. Lamellae flesh pink (2.5YR 8/6), free, slightly crowded, thick, unequal, slightly ventricose, 3–5 mm wide. Stipe 37–48 × 4–7 mm, cylindrical, hollow, partly spirally twisted, base slightly thick, fibrous, surface smooth. Odorless. Spore prints pink.

Basidiospores [150, 5, 3] 6.0–7.0(–7.5) × 5.0–6.0(–6.5) μm, avL × avW = 6.5–7.0 × 5.5–5.8 µm, Q = 1.15–1.40 μm, avQ = 1.18–1.26 μm, subglobose to broadly ellipsoid, ellipsoid or ovoid, slightly pinkish, smooth, thin-walled. Basidia 24–29 × 7–10 μm, clavate, thin-walled, 4-sterigmate, hyaline. Pleurocystidia 50–86 × 20–26 μm, numerous, scattered, fusiform to utriform, apically obtusely rounded, commonly provided with 1–4 digitiform excrescences at apex, slightly thick-walled, smooth, hyaline. Lamellar edge sterile. Cheilocystidia 35–72 × 13–25 μm, similar in form to pleurocystidia, numerous, narrowly fusiform to utriform, with 1–2 digitate or mucronate apically, thin-walled, hyaline. Pileipellis a trichoderm with radial hyphae, with terminal elements 43–157 × 13–31 μm, hyphae at the center extending outwards, with cylindrical or fusiform elements, thick-walled, with slightly yellowish intracellular pigment. Stipitipellis a cutis, hyphae 4–11 µm diam, cylindrical, hyaline, non-incrusted, non-gelatinous, and thin-walled. Caulocystidia absent. Clamp connections absent in all tissues.

###### Habitat.

Solitary to scattered on decaying wood (*Quercus*, *Picea*) in broad-leaved or mixed forests.

###### World distribution.

Europe (Rea 1922; Vellinga and Schreurs 1985; Orton 1986; Vellinga 1990; Breitenbach and Kränzlin 1995; Krieglsteiner 2003; Boccardo et al. 2008; Heilmann-Clausen 2008), Asia (Takehashi and Kasuya 2007), North America (Murrill 1917; Kauffman 1918; Singer 1956) and North Africa (Malençon and Bertault 1970).

###### China distribution.

It is distributed in all provinces of China (Xu 2016).

###### Additional specimens examined.

CHINA • Heilongjiang Province, Heihe Stewardship Station; On rotting wood in mixed solitary forests; 29 July 2021, Z.X. Qi, FJAU66580 (Collection no.: Qi 522) (ITS: PP516612, LSU: PP516662, *tef1*: PP551596). CHINA • Heilongjiang Province, Shuanghe National Nature Reserve; Scattered on decaying wood in mixed forests; 13 July 2019, D.Z. Guo, FJAU66581 (Collection no.: Guo 385) (ITS: PP516611, LSU: PP516661, *tef1*: PP551597). CHINA • Heilongjiang Province, Shuanghe National Nature Reserve; Scattered on decaying wood in mixed forests; 19 July 2019, D.Z. Guo, FJAU66582 (Collection no.: Guo 070) (ITS: PP516610, LSU: PP516660, *tef1*: PP551598).

###### Notes.

The primary distinguishing characteristics of *Pluteusleoninus* include a bright yellow to orange-yellow pileus that is rugose in the middle, a slightly yellowish or yellowish-white stipe, most subglobose to broadly ellipsoid, a few globose or ellipsoid.

*P.leoninus* displays a range of pileus colors, including yellowish brown, yellowish orange, yellow, or lemon yellow in young and mature specimens, which may lead to confusion with sect. Hispidodermaor evensect.Celluloderma, including *P.roseipes*, *P.fenzlii*, *P.chrysaegis*, *P.chrysophlebius*, and *P.luteomarginatus*. *P.leoninus* can be differentiated from the pinkish to pale reddish pileus of *P.roseipes* by its yellow or yellowish-brown pileus (Ludwig 2007). *P.leoninus* can be distinguished from *P.fenzlii* by its stipe lacking an annulus, a less squamulose pileus, and by examining the structure and dimensions of both the pleurocystidia and the pileipellis (Malysheva et al. 2007). *P.chrysaegis* differs from *P.leoninus* by possessing short pileipellis elements extending up to 40 μm (Pradeep et al. 2012; Lezzi et al. 2014). *P.chrysophlebius* is characterized by a shorter stipe and pileus, with an unveined bright yellow pileus lacking brownish shading (Vellinga 1990). On the other hand, *P.luteomarginatus* features a pileus with brownish shading, particularly prominent at the center and with a bright yellow margin, along with more subglobose basidiospores (7.5 × 6 μm) (Takehashi and Kasuya 2007).

The phylogenetic tree results were consistent with the morphological results. Specimens from China in *P.leoninus* clustered in the same branch as Russia, France, USA, and Turkey with high support (MLB = 100, BPP = 0.99, Fig. 4).

##### ﻿ Pluteus variabilicolor

Taxon classificationFungiAgaricalesPluteaceae

Babos, Annls Hist.–Nat. Mus. Natn. Hung. (1978: 93)

736E6338-A1BE-5868-A96C-AF3A6F8665C3

Figs 26A–L , 30

###### Description.

Basidiomata small to medium-sized. Pileus 21–42 mm diam; campanulate to hemispherical when young, egg-yellow (5.0Y 9/12); gradually plano-convex at maturity, bright yellow (5.0Y 9/18), brown in the middle (7.5YR 7/18); the white margin, with striate from the middle to the margin, straight. Context yellowish (2.5Y 8/18), odorless, 2–4 mm thick. Lamellae initially white, becoming pink to flesh-pink at maturity (2.5Y 9/12–2.5Y 8/12), free, crowded, thick, unequal, 3–5 mm wide. Stipe 37–75 × 6–11 mm, cylindrical to compressed, hollow, white, fibrous, base light brown (2.5Y 8/16), slightly thick to bulbous, surface relatively smooth. Odorless. Spore prints pink.

Basidiospores [200, 12, 7] 5.0–5.5(–6.0) × 4.5–5.0(–5.5) μm, avL × avW = 5.2–5.5 × 4.8–5.0 µm, Q = 1.00–1.33 μm, avQ = 1.10–1.15 μm, spherical to subglobose, slightly pinkish, smooth, thin-walled. Basidia 24–30 × 7–10 μm, clavate, thin-walled, 4-sterigmate, hyaline. Pleurocystidia 55–87 × 12–24 μm, scattered, fusiform, lageniform, or utriform, some with or without 1–2 digitiform excrescences at apex, thin-walled, smooth, hyaline. Lamellar edge sterile. Cheilocystidia 31–68 × 9–16 μm, numerous, clustered, subfusiform to fusiform, apically diverticulate or shortly rostrate, thin-walled, hyaline. Pileipellis a hymeniderm formed of variable and often mixed elements, from short and rounded to clavate or cylindrical terminal elements, 62–101 × 22–36 μm, with yellow intracellular pigment, with thin and smooth walls. Stipitipellis a cutis, hyphae 5–12 µm diam, cylindrical, hyaline, thin-walled. Caulocystidia 53–88 × 11–22 μm, mostly grouped, fusiform, lageniform to broadly lageniform, apical with short rostrate, thin-walled, smooth, hyaline. Clamp connections absent in all tissues.

###### Habitat.

Solitary to scattered on rotting wood in broad-leaved or mixed forests.

###### World distribution.

Hungary (Babos 1978), Europe from Austria (Lohmeyer et al. 1994), Italy (Lanconelli et al. 1998; Migliozzi 2011; Lezzi et al. 2014), Romania (Béres 2012), Russia (as *P.castri*) (Justo et al. 2011a), Slovenia (Jogan et al. 2012), Moldova (Lezzi et al. 2014), Japan (as *P.castri*) (Justo et al. 2011a), Germany (Ludwig 2007), Turkey (Kaygusuz et al. 2019), China (as *P.luteus*) (Ševčíková and Dima 2021).

###### China distribution.

Henan Province, Sichuan Province, Jiangsu Province.

###### Additional specimens examined.

CHINA • Henan Province, Uyang Minzhuang Forestry; 32°52'44.77"N, 113°36'34.87"E; Scattered in mixed forests dominated by *Quercusserrata*; 25 July 2023, Y.J. Liu, FJAU66583 (Collection no.: Liu 797) (ITS: PP516613, LSU: PP516663, *tef1*: PP551599). CHINA • Henan Province, Uyang County Yihezhai Forest Area; 32°52'42.46"N, 113°36'54.17"E; Scattered on dead wood stumps in Sargasso pine forests (*Pinusmassoniana*); 1 August 2023, Y.J. Liu, FJAU66584 (Collection no.: Liu 858) (ITS: PP516614, LSU: PP516664, *tef1*: PP551600). CHINA • Henan Province, Uyang Minzhuang Forestry; 32°52'84.97"N, 113°36'39.77"E; Scattered on dead wood stumps in Sargasso pine forests (*P.massoniana*); 2 August 2023, Y.J. Liu, FJAU66585 (Collection no.: Liu 881) (ITS: PP516615, LSU: PP516665, *tef1*: PP551601). CHINA • Henan Province, Uyang Minzhuang Forestry; 32°52'89.37"N, 113°36'38.27"E; Scattered on dead wood stumps in Sargasso pine forests (*P.massoniana*); 2 August 2023, Y.J. Liu, FJAU66586 (Collection no.: Liu 889) (ITS: PP516616, LSU: PP516666, *tef1*: PP551602). CHINA • Henan Province, Uyang County Tongshan Lake Forest National Park; 32°52'79.87"N, 113°36'58.37"E; Scattered on dead wood stumps in Sargasso pine forests (*P.massoniana*); 12 August 2023, Y.J. Liu, FJAU66587 (Collection no.: Liu 982) (ITS: PP516617, LSU: PP516667, *tef1*: PP551603). CHINA • Sichuan Province, Tongjiang County, Chenhe Township; 32°12'24.57"N, 107°12'53.83"E; 2 August 2023, L. B. Wang, FJAU66588 (Collection no.: Wang 1106) (ITS: PP516618, LSU: PP516668, *tef1*: PP551604). CHINA • Sichuan Province, Tongjiang County, Chenhe Township; 32°12'24.55"N, 107°12'53.25"E; 2 August 2024, L. B. Wang, FJAU66623 (Collection no.: Wang 1674) (ITS: PQ810763, LSU: PQ810740, *tef1*: PQ811063).

###### Notes.

*Pluteusvariabilicolor* is characterized by the lively yellowish color of the pileus, the dark velvety stipe when young, the presence of caulocystidia and a pileipellis variable elements (Babos 1978; Kaygusuz et al. 2019).

Macroscopically, *P.variabilicolor* is very similar to *P.leoninus*, but the two taxa are distinguished by the structure of the pileipellis and the presence or absence of caulocystidia. The pileipellis structure of *P.variabilicolor* is hymeniderm, consisting of elongated cylindrical hyphae and rounded terminal elements that are clavate in shape, whereas the pileipellis structure of *P.leoninus* is trichohymeniderm, consisting of uniformly narrow fusiform elements. In addition, *P.variabilicolor* has caulocystidia, whereas *P.leoninus* does not (Takehashi and Kasuya 2007; Kaygusuz et al. 2019). Similarly, the pileus of both *P.variabilicolor* and *P.chrysaegis* was yellow or yellowish with veined surfaces, and the general structure of the pileipellis and the presence of caulocystidia were both observed (Pradeep et al. 2012; Lezzi et al. 2014; Kaygusuz et al. 2019). However, we can distinguish between the two by the presence of cheilocystidia, which are mucronate and thin-walled in *P.variabilicolor*, and non-mucronate and slightly thick-walled in *P.chrysaegis* (Babos 1978; Pradeep et al. 2012; Lezzi et al. 2014; Hosen et al. 2018; Kaygusuz et al. 2019).

The phylogenetic tree results based on the ITS+*tef1* dataset were consistent with the morphological results. Specimens from China in *P.variabilicolor* clustered in the same branch as Japan, Russia, Turkey, China and Hungary with high support (MLB = 100, BPP = 1, Fig. 4).

### ﻿Key to the reported species of Pluteussect.Hispidoderma in China

**Table:** 

1	Caulocystidia present	**2**
–	Caulocystidia absent	**18**
2	Cheilocystidia top with small horns or mucronate	**3**
–	Cheilocystidia top without small horns or mucronate	**11**
3	Pileus yellow or bright yellow colors	**4**
–	Pileus without yellow or bright yellow colors	**6**
4	Cheilocystidia thick-walled	***P.chrysaegis***
–	Cheilocystidia thin-walled	**5**
5	Pileus yellow to ochre, mostly clavate or fusiform pleurocystidia and cheilocystidia often with an obtuse apex or rostrum	***P.variabilicolor***
–	Pileus bright yellow, pleurocystidia fusiform to subfusiform with 1–2 digitiform excrescences, cheilocystidia with rostrate	***P.ussuriensis***
6	Lamellae sparse	***P.spaniophyllus***
–	Lamellae not sparse	**7**
7	Pileus surface with distinctive vein-like streaks	**8**
–	Pileus surface without distinctive vein-like streaks	**10**
8	Basidiomata overall reddish-brown	***P.umbrosus***
–	Basidiomata overall not reddish-brown	**9**
9	Pileus surface with brown triangular conical scales, Stipe white	***P.umbrosoides***
–	Pileus surface with tan vein-like projections, Stipe brown	***P.costatus***
10	Pleurocystidia without transverse septum	***P.jilinensis***
–	Pleurocystidia with transverse septum	***P.velutinus***
11	Pileus surface with brown glandular dots	**12**
–	Pileus surface without brown glandular dots	**13**
12	Pileus not exceeding the margin of lamellae	***P.granularis***
–	Pileus exceeding the margin of lamellae	***P.baishanzuensis***
13	Pileus surface with scales	**14**
–	Pileus surface without scales	**15**
14	Pileus surface densely white ciliated, smooth when wet	***P.albivillus***
–	Pileus earthy yellow with a central cocked brown scale	***P.hinnuleus***
15	Pleurocystidia apically mucronate or rostrate	***P.tenuipileus***
–	Pleurocystidia apically not mucronate or rostrate	**16**
16	Basidiospores broadly ellipsoid to ellipsoid	***P.ultraputripiceae***
–	Basidiospores not broadly ellipsoid to ellipsoid	**17**
17	Pileus charred yellow to light yellow, pleurocystidia fusiform to broadly subfusiform	**P.aff.semibulbosus**
–	Pileus brownish-brown, pleurocystidia fusiform or narrowly utriform	***P.longistriatus***
18	Basidiospores small (avL < 5.5, avW < 4	**17**
–	Basidiospores large (avL ≥ 5.5, avW ≥ 4	**20**
19	Pileus surface shortly tomentose, or tufted, stipe with brown tufted tomentose	***P.fenghuangensis***
–	Basidiomata small, pileus dark gray to slightly yellowish, cheilocystidia sub clavate or long fusiform, thick-walled	***P.microsporus***
20	Pileus with yellow, orange or reddish colors	**21**
–	Pileus without yellow, orange or reddish colors	**23**
21	With cheilocystidia	**22**
–	Without cheilocystidia	***P.rimosus***
22	Pileus with a brown pruinose on the surface	***P.piceicola***
–	Pileus bright yellow to orange-yellow, middle brown or wrinkled, stipe slightly yellowish to yellowish-white, basidiospores subglobose to broadly ellipsoid	***P.leoninus***
23	Pileus surface non-rough	***P.albidus***
–	Pileus surface rough	**24**
24	Pileus yellowish brown to reddish brown, with distinctive spots on the surface	***P.pantherinus***
–	Pileus alutaceous to fuliginous, with a velvety brown to blackish brown stipe and stipe context	***P.plautus***

## ﻿Discussion

### ﻿Phylogenetic taxonomy of sect.Hispidoderma species

The phylogenetic analyses of Pluteussect.Hispidoderma reveals a clear subdivision into three distinct evolutionary lineages: the /*plautus* clade, the /*leoninus* clade, and the /*umbrosus* clade, and one lineage: the /*pantherinus* lineage. Our study, examining 18 species within this section, provides critical insights into the taxonomic relationships and evolutionary history of these fungi.

Both molecular and morphological evidence supports this tripartite division. The /*plautus* clade is characterized by variable pileus coloration and basidiospores that are generally subglobose to ellipsoid. Species in the /*leoninus* clade typically display vibrant yellow to orange pigmentation in both pileus and stipe features. Meanwhile, the /*umbrosus* clade is distinguished by granular to squamulose pileus surfaces and dark pigmentation at the lamellae edges.

While the overall branching pattern of our phylogeny is well-supported, certain positions within the topology remain unresolved. For instance, as noted in Justo et al. (2025), the phylogenetic position of *P.aureus* remains ambiguous. Similarly, in our current analysis, *P.semibulbosus* and *P.plautus* appear unresolved, suggesting the need for additional molecular markers or expanded taxon sampling to fully clarify relationships within these problematic lineages.

Regarding the /*pantherinus* lineage, previous studies have positioned it as a sister group to the /*leoninus* clade. However, based on our morphological analyses and phylogenetic construction, we propose its repositioning as a sister group to the /umbrosus clade.

In our phylogenetic analyses, the /*pantherinus* lineage received strong support (MLB = 87, BPP = 0.91, Fig. 1; MLB = 100, BPP = 1, Fig. 3), which is generally higher than the support values reported by Desjardin and Perry (2018), Malysheva et al. (2023), and Justo et al. (2025) for certain species within this lineage.

Morphological evidence further substantiates this repositioning. For instance, *P.ornatus* is characterized by its densely squamulose and dark brown pileus, lamellae with brown edges, features that closely resemble *P.umbrosus* and *P.umbrosoides* (Malysheva et al. 2023). *P.saisamorniae* exhibits its squamules and grayish brown to light brown pileus, grayish orange stipe covered by brown minutely dotted patterns, and lamellae with light brown edges, sharing similarities with *P.umbrosus*, *P.umbrosoides*, and *P.granularis* (Wannathes et al. 2022). Meanwhile, *P.conizatus* is distinguished by a surface distinctly rugose or rugose-venose around the center, with age breaking up into distinct, often pulverulent, squamules, at least around the center; older specimens becoming predominantly pale yellow, pale yellow-brown, or pale gray-brown, darker at the center. Lamellae with even or flocculose edges. These characteristics align closely with *P.pantherinus* (Justo et al. 2025).

Collectively, the /*pantherinus* lineage and /*umbrosus* clade share multiple morphological features: brownish tones in the pileus and stipe, squamulose pileus surface, and pigmented lamellar edges (in most cases). In contrast, the /*leoninus* clade typically displays yellowish tones in both pileus and stipe. Based on these phylogenetic and morphological lines of evidence, we conclude that positioning the /*pantherinus* lineage as a sister group to the /*umbrosus* clade is more appropriate.

### ﻿Evolutionary relationships between species and hosts of sect.Hispidoderma

Pluteussect.Hispidoderma species can rot directly on top of trunks, stumps, branches, or buried limbs of living or dead trees or grow above ground. While examining the trees identified as the main hosts (Fig. 1), we discovered that Angiosperm hosted 33 species (78%), while Gymnosperm hosted 16 species (38%). When Angiosperm was the host, *Fagus*, *Populus*, *Quercus*, or *Acer* were dominant, and when Gymnosperm was the host, *Pinus*, *Picea*, and *Platycladus* were dominant. Some species can rot in both Angiosperms and Gymnosperms, such as *P.granularis*. Thus, we believe that species of sect.Hispidoderma has different preferences for different trees regarding basidiomata growth, with Angiosperm trees being more favorable for basidiomata development. This may be related to the wide distribution of angiosperms worldwide (Qian et al. 2024). We indicate that the species differentiation of sect.Hispidoderma is closely linked to the substrate plants. For example, *Pleurotusostreatus* (oyster mushroom) demonstrates a clear distribution pattern that follows its preferred deciduous hosts, particularly *Fagus* and *Quercus* species across Europe and North America (Zervakis et al. 2004). Molecular studies by Vilgalys et al. (1993) revealed that *Pleurotus* populations have developed genetic adaptations specific to their local host trees, showing how fungal evolution is influenced by host specialization. Similarly, Li et al. (2020) found that a combination of environmental changes and substrate transitions contributed to the diversification of *Pleurotus* species.

Some species, including *P.fenghuangensis*, *P.plautus*, and *P.fibrillosus*, can grow on the ground, similar to *P.ephebeus* and *P.aletaiensis* reported by Qi et al. (2022). In conclusion, our findings highlight the intimate evolutionary relationship between Pluteussect.Hispidoderma species and their host trees, particularly their preference for Angiosperm substrates. Future research should combine genomic analyses, enzyme characterization, and co-phylogenetic studies to unravel the mechanisms driving these host-fungus associations. Understanding these relationships will not only illuminate fungal diversification processes but also contribute to broader ecological theories on species coexistence in forest ecosystems.

### ﻿Integration of morphology, phylogeny, substrate, and distribution in the classification of Pluteussect.Hispidoderma

In the taxonomic framework of Pluteussect.Hispidoderma, defining new species requires a more holistic approach that transcends the traditional reliance on stable morphological distinctions or phylogenetic positioning alone.

A comprehensive taxonomic assessment should integrate multiple lines of evidence: genetic distance measurements, substrate preferences, and geographical distribution patterns. Recent studies by Kaygusuz et al. (2021), Zhu and Bau (2024), Wang et al. (2024), and Justo et al. (2025) have demonstrated that genetic distances should not be interpreted as standalone evidence but rather as integral components within a polyphasic approach. These researchers found that while no universal genetic distance threshold defines species boundaries across all fungi, comparative studies across multiple genera including *Pluteus*, *Entoloma*, and *Cortinarius* have confirmed that ITS sequence divergence values of 2–5% often indicate species-level differentiation when corroborated by other evidence. Their analyses within a clade framework have determined which morphological characteristics remain stable, what degree of interspecific variation is taxonomically meaningful, and how substrate affinity and distribution patterns contribute to species boundaries. This body of research has consistently shown that intraspecific ITS variation typically remains below 1.5% in well-studied fungal species, while interspecific distances generally exceed this threshold—particularly when supported by morphological and ecological distinctions. However, taxonomic complexity appears at both extremes. At one end, several cosmopolitan species exhibit remarkably high intraspecific genetic diversity that challenges simple distance-based delimitation. Examples include *P.cervinus* (Eurasia, eastern and western North America), *P.hongoi* (Eurasia, eastern North America), *P.petasatus* (Eurasia, eastern and western North America), and *P.leucoborealis* (Eurasia, eastern and western North America) (Justo et al. 2025), where geographic isolation and regional adaptation have created substantial genetic heterogeneity within what are considered single biological species. At the other extreme, cases with minimal genetic distances require particularly robust morphological, ecological, or geographic evidence. As demonstrated by Justo et al. (2025) in the delineation of closely related species (*P.favrei*, *P.insularis*, *P.hesperius*), even with ITS divergence below typical thresholds, consistent morphological distinctions, substrate specificities, and biogeographic patterns may collectively justify species-level recognition.

By synthesizing these multiple dimensions of evidence—phylogenetic distinctiveness, significant genetic distances, consistent morphological differences, ecological preferences, and biogeographic patterns—taxonomists can establish robust taxonomic frameworks that better reflect the evolutionary history and ecological adaptation within fungal groups. This comprehensive approach allows for more confident taxonomic conclusions even in the absence of universal thresholds for species delimitation across fungal groups.

## ﻿Conclusion

Our study of Pluteussect.Hispidoderma employed an integrative approach combining morphological and molecular data to explore the section’s phylogenetic structure. Analysis of 319 sequences, including 32 type specimens, identified three main evolutionary clades (/*plautus* clade, /*leoninus* clade, /*umbrosus* clade) and one lineage (/*pantherinus* lineage). Through this investigation, we documented 18 species within the section, contributing to the current taxonomic understanding of this fungal group. The methodological approach used in this study combines multiple lines of evidence for species recognition, potentially offering a useful framework for similar taxonomic studies. While our findings help clarify some aspects of the taxonomic relationships within sect.Hispidoderma, further research will be needed to fully understand the evolutionary history and ecological dynamics of this diverse fungal section.

## Supplementary Material

XML Treatment for Pluteus tenuipileus

XML Treatment for Pluteus spaniophyllus

XML Treatment for Pluteus jilinensis

XML Treatment for Pluteus aff. semibulbosus

XML Treatment for Pluteus ultraputripiceae

XML Treatment for Pluteus hinnuleus

XML Treatment for Pluteus baishanzuensis

XML Treatment for Pluteus albivillus

XML Treatment for Pluteus velutinus

XML Treatment for Pluteus longistriatus

XML Treatment for Pluteus costatus

XML Treatment for Pluteus umbrosus

XML Treatment for Pluteus umbrosoides

XML Treatment for Pluteus granularis

XML Treatment for Pluteus piceicola

XML Treatment for Pluteus ussuriensis

XML Treatment for Pluteus leoninus

XML Treatment for Pluteus variabilicolor

## ﻿Additional information

### Use of AI

No use of AI was reported.

### Author ORCIDs

Zheng-Xiang Qi https://orcid.org/0000-0002-0037-9407

Li-Bo Wang https://orcid.org/0009-0004-4012-1648

Ke-Qing Qian https://orcid.org/0000-0002-4627-5240

Li-Li Shi https://orcid.org/0009-0007-1839-7375

Jia-Jun Hu https://orcid.org/0000-0002-7562-7612

Yong-Lan Tuo https://orcid.org/0000-0001-6019-1038

Gu Rao https://orcid.org/0000-0001-6199-9004

Muharagi Samwel Jacob https://orcid.org/0009-0002-1860-3603

Rui-Peng Liu https://orcid.org/0009-0005-0620-3655

Ming-Hao Liu https://orcid.org/0009-0003-5904-1210

Bo Zhang https://orcid.org/0000-0001-9508-8188

Xiao Li https://orcid.org/0000-0002-1230-6467

### Data availability

All sequence data generated for this study can be accessed via GenBank: https://www.ncbi.nlm.nih.gov/genbank/. All alignments for phylogenetic analyses were deposited in figshare: https://figshare.com/articles/dataset/dx_doi_org_10_6084_m9_figshare_25556958/25556958.

## Supplementary materials

Supplementary material 1 Supplementary tables S1, S2 and figures S1–S6 Data type docx Explanation note **table S1**: Genetic distance estimation of the selected sequences of Pluteussect.Hispidoderma species by Kimura 2-parameter model in ITS; **table S2**: Genetic distance estimation of the selected sequences of Pluteussect.Hispidoderma species by Kimura 2-parameter model in *tef1*; **figure S1**: Phylogenetic tree of the /*plautus* clade of the sect.Hispidoderma. The best tree from the ML and BI analysis of the ITS dataset; **figure S2**: Phylogenetic tree of the /*plautus* clade of the sect.Hispidoderma. The best tree from the ML and BI analysis of the *tef1* dataset; **figure S3**: Phylogenetic tree of the /*umbrosus* clade and /*pantherinus* lineage of the sect.Hispidoderma. The best tree from the ML and BI analysis of the ITS dataset; **figure S4**: Phylogenetic tree of the /*umbrosus* clade and /*pantherinus* lineage of the sect.Hispidoderma. The best tree from the ML and BI analysis of the *tef1* dataset; **figure S5**: Phylogenetic tree of the /*leoninus* clade of the sect.Hispidoderma. The best tree from the ML and BI analysis of the ITS dataset; **figure S6**: Phylogenetic tree of the /*leoninus* clade of the sect.Hispidoderma. The best tree from the ML and BI analysis of the tef1 dataset. https://binary.pensoft.net/file/1383999This dataset is made available under the Open Database License (http://opendatacommons.org/licenses/odbl/1.0/). The Open Database License (ODbL) is a license agreement intended to allow users to freely share, modify, and use this Dataset while maintaining this same freedom for others, provided that the original source and author(s) are credited.Zheng-Xiang Qi, Li-Bo Wang, Ke-Qing Qian, Li-Li Shi, Jia-Jun Hu, Yong-Lan Tuo, Gu Rao, Muharagi Samwel Jacob, Rui-Peng Liu, Ming-Hao Liu, Di-Zhe Guo, Ya-Jie Liu, Bo Zhang, Xiao Li, Yu Li
